# Ion-pairing assemblies of π-extended anion-responsive organoplatinum complexes

**DOI:** 10.1080/14686996.2024.2313958

**Published:** 2024-02-06

**Authors:** Yohei Haketa, Yu Murakami, Hiromitsu Maeda

**Affiliations:** Department of Applied Chemistry, College of Life Sciences, Ritsumeikan University, Kusatsu, Japan

**Keywords:** Pt^II^ complexes, anion-responsive π-electronic systems, ion-pairing assemblies

## Abstract

Pt^II^ complexes of π-extended dipyrrolyldiketones were synthesized as anion-responsive π-electronic molecules. The dipyrrolyldiketone Pt^II^ complexes exhibited red-shifted absorption and photoluminescence properties. In the solid state, [1 + 1]-type anion complexes formed charge-by-charge ion-pairing assemblies when combined with countercations. Detailed theoretical studies of the packing structures revealed favorable interactions between the planar anion complexes and π-electronic cations.

## IMPACT STATEMENT

Pt^II^ complexes of π-extended dipyrrolyldiketones, introducing arylethynyl substituents, in the form of anion complexes exhibited the formation of charge-by-charge assemblies with π-electronic cations via ^*i*^π–^*i*^π interactions.

## GRAPHIC ABSTRACT

**Figure:**
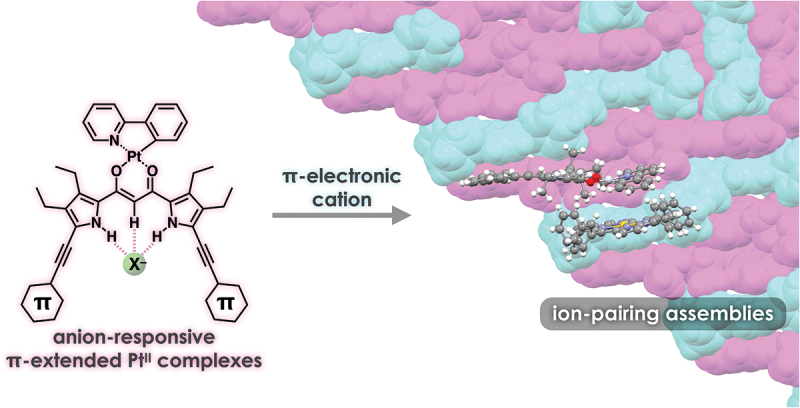

## 1. Introduction

**Figure 1.**
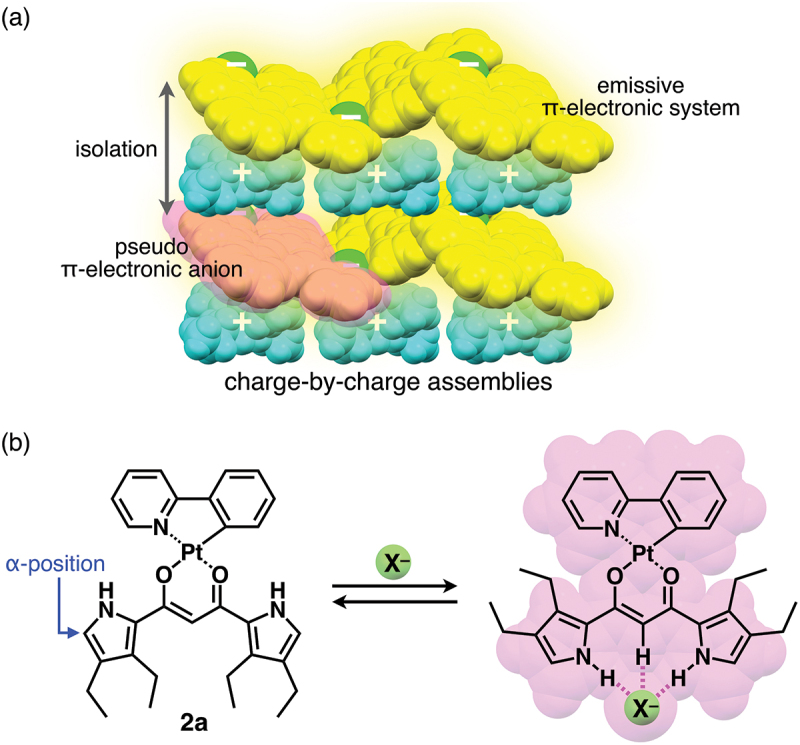
(a) Conceptual diagram for emissive charge-by-charge assembly comprising emissive π-electronic anion and bulky cation and (b) [1 + 1]-type anion-binding mode of dipyrrolyldiketone Pt^II^ complex **2a**.

π-Electronic systems exhibit fascinating electronic and electrooptical properties based on the tunable electronic states that affect their absorption and luminescence properties. The assembly of π-electronic systems in an ordered arrangement results in a variety of supramolecular nanoarchitectonics [1,2] to show unique electrooptical properties that cannot be observed in single molecules [3–7]. In particular, the solid-state photophysical properties of π-electronic systems depend on the stacking arrangement of their components. Among the various solid-state luminescent materials, organoplatinum(II) complexes exhibit various photoluminescence properties such as intense phosphorescence from triplet excited states [8–13]. Assemblies with an ordered arrangement of organoplatinum(II) complexes are suitable for the applications in light-emitting devices. Recent studies on the micro- and nanocrystals of organoplatinum(II) complexes have also exhibited triplet energy transfer and phosphorescence anisotropy amplification [14,15]. π-Extended ligands in organoplatinum(II) complexes modulate the photophysical properties of single molecules as well as those in assembled states. Stacking of organoplatinum(II) complexes in the assembled state often interferes with photoluminescence [16]. Strategies for maintaining photoluminescence by isolating π-electronic molecules in the solid state have been reported [17,18]. The introduction of bulky substituents to the peripheral ligands prevents the Pt^II^ complexes from stacking. Furthermore, ion-pairing assemblies [19] of charged emissive species and appropriate counterions can yield photoluminescent materials (Figure 1(a)) [20,21]. For example, solid-state ion-pairing assemblies of non-emissive receptor–anion complexes and emissive countercations exhibit enhanced emission [22,23]. Controlling the ordered arrangement of emissive π-electronic molecules in the solid state is important for the fabrication of photoluminescent materials.

Among the various phosphorescent organoplatinum(II) complexes (e.g. **2a**, Figure 1(b)), dipyrrolyldiketone Pt^II^ complexes bearing arylpyridine ligands exhibit efficient anion-binding behavior via hydrogen bonding at the pyrrole NH and bridging CH (Figure 1(b)) [24]. Modification of arylpyridine ligands results in red-shifted phosphorescence properties [25]. The square planar dipyrrolyldiketone Pt^II^ complexes exhibit non-emissive solid states owing to the efficient π–π stacking. In contrast, ion-pairing assemblies of the anion complexes and counter alkylammonium cations show enhanced phosphorescence behavior owing to their charge-by-charge assembly mode (Figure 1(a)). The emissive anion complexes are spatially isolated by counter alkylammonium cations, resulting in enhanced phosphorescence derived from the monomeric anion complexes. The solid-state phosphorescence properties, such as emission wavelength, can be tuned by the π-extension of ligands. Thus far, modifications at the pyrrole α-positions of dipyrrolyldiketone Pt^II^ complexes have been limited to the substitution with 2,6-dimethylphenyl moieties. In this study, Pt^II^ complexes of π-extended dipyrrolyldiketones were synthesized to evaluate ion-pairing assemblies of Pt^II^ complexes in their anion-binding forms with π-electronic cations.

## 2. Experimental section

### 2.1. Synthesis and characterization

#### 2.1.1. General procedures

Starting materials were purchased from FUJIFILM Wako Pure Chemical Corp., Nacalai Tesque Inc., Tokyo Chemical Industry Co., Ltd., Sigma-Aldrich Co., and Tanaka Kikinzoku Kogyo K.K. and were used without further purification unless otherwise stated. Nuclear magnetic resonance (NMR) spectra used in the characterization of products were recorded on a JEOL ECA-600 600 MHz spectrometer. All NMR spectra were referenced to solvent. UV-visible absorption spectra were recorded on a Hitachi U-3500 spectrometer. Matrix-assisted laser desorption ionization time-of-flight mass spectrometry (MALDI-TOF-MS) was recorded on a Shimadzu Axima-CFRplus. TLC analyses were carried out on aluminum sheets coated with silica gel 60 (Merck 5554). Column chromatography was performed on Wakogel C-300.

#### 2.1.2. 1,3-Bis(3,4-diethyl-5-phenylethynylpyrrol-2-yl)-1,3-propanedione, 1b´

In a round-bottomed flask, according to the procedure that has been modified from the previous one [26], BF_2_ complex of 1,3-bis(3,4-diethyl-5-phenylethynylpyrrol-2-yl)-1,3-propanedione [27,28] **1b** (240 mg, 0.427 mmol) and LiOH·H_2_O (1.31 g, 31.2 mmol) were dissolved in 1,4-dioxane (90 mL) and water (90 mL) under N_2_ atmosphere. To the solution was added acetic acid (AcOH) (66 μL, 1.17 mmol). The reaction mixture was stirred at 90°C for 2.5 h. The solution was acidified dropwise with 1 M HCl aq at pH 7. After the removal of the solvent under vacuum, the residue was then partitioned between water and CH_2_Cl_2_. The organic phase was dried over anhydrous Na_2_SO_4_, and was evaporated under vacuum. The residue was then purified by chromatography over silica gel column (Wakogel C-300; eluent: CHCl_3_) and was recrystallized from CH_2_Cl_2_/*n*-hexane to give **1b´** (182 mg, 0.354 mmol, 83%) as a yellow solid. *R*_*f*_ = 0.75 (CH_2_Cl_2_). ^1^H NMR (600 MHz, DMSO-*d*_6_, 20°C; diketone **1b´** was obtained as a mixture of keto and enol tautomers in the ratio of 0.59:1): *δ* (ppm) keto from 12.06 (br, 2 H, NH), 7.53 (d, *J* = 7.2 Hz, 4 H, Ar-H), 7.43 (t, *J* = 6.9 Hz, 6 H, Ar-H), 4.33 (s, 2 H, bridging CH_2_), 2.69 (q, *J* = 7.8 Hz, 4 H, CH_2_), 2.57–2.54 (m, 4 H, CH_2_), 1.18–1.13 (m, 12 H, CH_3_); enol from 11.74 (br, 2 H, NH), 7.53 (d, *J* = 7.2 Hz, 4 H, Ar-H), 7.43 (t, *J* = 6.9 Hz, 6 H, Ar-H), 6.49 (s, 1 H, bridging CH), 2.77 (q, *J* = 7.8 Hz, 4 H, CH_2_), 2.57–2.54 (m, 4 H, CH_2_), 1.18–1.13 (m, 12 H, CH_3_). ^13^C{^1^H} NMR (151 MHz, DMSO-*d*_6_, 20°C): *δ* (ppm) 184.56, 176.04, 132.22, 132.09, 131.47, 131.06, 131.10, 130.84, 128.98, 128.96, 128.92, 128.52, 125.29, 122.26, 122.10, 115.05, 114.75, 94.43, 93.68, 92.29, 81.60, 81.40, 50.85, 18.08, 18.02, 17.49,17.30, 15.92, 15.75, 15.73, 15.38. MALDI-TOF-MS (% intensity): *m*/*z*: 513.3 (100), 514.3 (80), 515.3 (20). Calcd for C_35_H_33_N_2_O_2_ ([M – H]^–^): 513.25.

#### 2.1.3. 1,3-Bis(3,4-diethyl-5-(4-fluorophenyl)ethynylpyrrol-2-yl)-1,3-propanedione, 1c´

In round-bottomed flask, according to the procedure that has been modified from the previous one [26], BF_2_ complex of 1,3-bis(3,4-diethyl-5-(4-fluorophenyl)ethynylpyrrol-2-yl)-1,3-propanedione [27,28] **1c** (7.4 mg, 0.012 mmol) and LiOH.H_2_O (20.1 mg, 0.479 mmol) were dissolved in 1,4-dioxane (30 mL) and water (3 mL) under N_2_ atmosphere. To the solution was added acetic acid (3.0 μL, 0.053 mmol). The reaction mixture was stirred at 90°C for 12 h. The solution was acidified dropwise with 1 M HCl aq at pH 7. After the removal of the solvent under vacuum, the residue was then partitioned between water and CH_2_Cl_2_. The organic phase was dried over anhydrous Na_2_SO_4_, and was evaporated under vacuum. The residue was then purified by chromatography over silica gel column (Wakogel C-300; eluent: CHCl_3_) and was recrystallized from CH_2_Cl_2_/*n*-hexane to give **1c´** (5.94 mg, 0.011 mmol, 87%) as a yellow solid. *R*_*f*_ = 0.80 (CH_2_Cl_2_). ^1^H NMR (600 MHz, DMSO-*d*_6_, 20°C; diketone **1c´** was obtained as a mixture of keto and enol tautomers in the ratio of 0.51:1): *δ* (ppm) keto from 12.05 (br, 2 H, NH), 7.58–7.56 (m, 4 H, Ar-H), 7.29 (t, *J* = 8.4 Hz, 4 H, Ar-H), 4.31 (s, 2 H, bridging CH_2_), 2.68 (q, *J* = 7.8 Hz, 4 H, CH_2_), 2.55–2.51 (m, 4 H, CH_2_), 1.16–1.11 (m, 12 H, CH_3_); enol from 11.74 (br, 2 H, NH), 7.58–7.56 (m, 4 H, Ar-H), 7.29 (t, *J* = 8.4 Hz, 4 H, Ar-H), 6.46 (s, 1 H, bridging CH), 2.76 (q, *J* = 7.8 Hz, 4 H, CH_2_), 2.55–2.51 (m, 4 H, CH_2_), 1.16–1.11 (m, 12 H, CH_3_). ^13^C{^1^H} NMR (151 MHz, DMSO-*d*_6_ 20°C): *δ* (ppm) 184.43, 175.96, 162.85, 162.81, 161.21, 161.16, 133.38, 133.32, 133.31, 133.25, 132.12, 131.99, 131.39, 130.73, 128.49, 125.25, 118.70, 118.68, 118.55, 118.53, 116.24, 116.21, 116.09, 116.07, 114.85, 114.56, 93.25, 92.50, 92.24, 91.30, 81.09, 70.76, 17.99, 17.91, 14.40, 17.21, 15.79 15.61, 15.29. MALDI-TOF-MS (% intensity): *m*/*z*: 549.24 (100), 550.2 (55), 551.2 (15). Calcd for C_35_H_31_F_2_N_2_O_2_ ([M – H]^–^): 549.24. This compound was further characterized by single-crystal X-ray analysis.

#### 2.1.4. (1,3-Bis(3,4-diethyl-5-phenylethynylpyrrol-2-yl)-1,3-propanedionato-κ^2^O,O´)[2-(2-pyridinyl-κN)phenyl-κC]platinum, 2b

According to the literature procedure [24,25], in a dried round-bottomed flask, [(PtMe_2_)_2_(µ-SMe_2_)_2_] [29] (50 mg, 0.086 mmol) was dissolved in tetrahydrofuran (THF) (1.5 mL) under N_2_ atmosphere. To the solution was added 2-phenylpyridine (ppy) (25 μL, 0.16 mmol). The resulting mixture was stirred at room temperature (r.t.) for 1 h, and trifluoromethanesulfonic acid (TfOH) (15 μL, 0.17 mmol) was added dropwise. The reaction mixture was stirred for 1 h, and then a solution of **1b´** (41.2 mg, 0.080 mmol) and K_2_CO_3_ (50.6 mg, 0.361 mmol) in THF (5 mL) was added. The mixture was stirred for 3 h. After the removal of the solvent under vacuum, the residue was purified with column chromatography over silica gel (Wakogel C-300; eluent: CH_2_Cl_2_/*n*-hexane = 3/2) to give **2b** (12.8 mg, 14.8 µmol, 15%) as an orange solid. Silica gel column chromatography and recrystallization processes were conducted under dark condition by covering with aluminum foil. *R*_*f*_ = 0.67 (CH_2_Cl_2_/*n*-hexane = 3/2 (v/v)). m.p.: 146°C. ^1^H NMR (600 MHz, DMSO-*d*_6_, 20°C): *δ* (ppm) 11.58 (br, 1 H, NH), 11.45 (br, 1 H, NH), 9.04 (d, *J* = 5.4 Hz, 1 H, Ar-H), 8.07 (t, *J* = 7.5 Hz, 1 H, Ar-H), 8.02 (d, *J* = 7.8 Hz, 1 H, Ar-H), 7.68 (d, *J* = 7.8 Hz, 1 H, Ar-H), 7.59 (d, *J* = 7.2 Hz, 1 H, Ar-H), 7.55–7.53 (m, 4 H, Ar-H), 7.45–7.40 (m, 7 H, Ar-H), 7.13 (t, *J* = 7.2 Hz, 1 H, Ar-H), 7.08 (t, *J* = 6.6 Hz, 1 H, Ar-H), 6.31 (s, 1 H, bridging CH), 2.94–2.86 (m, 4 H, CH_2_), 2.60–2.58 (m, 4 H, CH_2_), 1.26–1.18 (m, 12 H, CH_3_). UV/vis (CH_2_Cl_2_, λ_max_[nm] (ε, 10^4^ M^−1^cm^−1^)): 462 (6.6). MALDI-TOF-MS (% intensity): *m*/*z*: 860.3 (70), 861.4 (100), 862.3 (80). Calcd for C_46_H_40_N_3_O_2_Pt ([M – H]^–^): 860.28. This compound was further characterized as the Cl^–^ complex (ion pairs) by single-crystal X-ray analysis.

#### 2.1.5. (1,3-Bis(3,4-diethyl-5-(4-fluorophenyl)ethynylpyrrol-2-yl)-1,3-propanedionato-κ^2^O,O´)[2-(2-pyridinyl-κN)phenyl-κC]platinum, 2c

According to the literature procedure [24,25], in a dried round-bottomed flask, [(PtMe_2_)_2_(µ-SMe_2_)_2_] [29] (39.2 mg, 0.0679 mmol) was dissolved in THF (2 mL) under N_2_ atmosphere. To the solution was added ppy (21.1 μL, 0.136 mmol). The resulting mixture was stirred at r.t. for 1 h, and TfOH (15 μL, 0.14 mmol) was added dropwise. The reaction mixture was stirred for 1 h, and then a solution of **1c´** (74.8 mg, 0.136 mmol) and NaOH (5.40 mg, 0.135 mmol) in THF (3.5 mL) and MeOH (0.5 mL) was added. The mixture was stirred for 14 h. After the removal of the solvent under vacuum, the residue was purified with column chromatography over silica gel (Wakogel C-300; eluent: CH_2_Cl_2_/*n*-hexane = 3/2) to give **2c** (38.6 mg, 43.0 µmol, 32%) as an orange solid. Silica gel column chromatography and recrystallization processes were conducted under dark condition by covering with aluminum foil. *R*_*f*_ = 0.72 (CH_2_Cl_2_/*n*-hexane = 3/2 (v/v)). Decomposed at > 220°C without melting. ^1^H NMR (600 MHz, CDCl_3_, 20°C): *δ* (ppm) 9.14 (br, 1 H, NH), 9.01 (d, *J* = 6.0 Hz, 1 H, Ar-H), 8.93 (br, 1 H, NH), 7.86 (t, *J* = 8.4 Hz, 1 H, Ar-H), 7.66 (d, *J* = 7.8 Hz, 1 H), 7.62 (d, *J* = 7.8 Hz, 1 H, Ar-H), 7.54–7.49 (m, 5 H, Ar-H), 7.30 (t, *J* = 7.2 Hz, 1 H, Ar-H), 7.21 (t, *J* = 6.9 Hz, 1 H, Ar-H), 7.15 (t, *J* = 6.9 Hz, 1 H, Ar-H), 7.08 (t, *J* = 7.8 Hz, 4 H, Ar-H), 6.35 (s, 1 H, bridging CH), 2.86–2.80 (m, 4 H, CH_2_), 2.66–2.62 (m, 4 H, CH_2_), 1.31–1.23 (m, 12 H, CH_3_). UV/vis (CH_2_Cl_2_, λ_max_[nm] (ε, 10^4^ M^−1^cm^−1^)): 460 (7.9). MALDI-TOF-MS (% intensity): *m*/*z*: 896.2 (64), 897.3 (100), 898.2 (94). Calcd for C_46_H_38_F_2_N_3_O_2_Pt ([M – H]^–^): 897.26. This compound was further characterized as the Cl^–^ complex (ion pair) by single-crystal X-ray analysis.

### 2.2. Method for single-crystal X-ray analysis

Crystallographic data are summarized in Table 1. A single crystal of **1c´** was obtained by vapor diffusion of *n*-hexane into a CH_2_Cl_2_ solution. The data crystal was a yellow block of approximate dimensions 0.135 mm × 0.135 mm × 0.088 mm. A single crystal of **2b**·Cl^–^-TBA^+^ (TBA^+^ = tetrabutylammonium) was obtained by vapor diffusion of *n*-hexane into an acetone solution of the mixture of **2b** and TBACl in the 1:1 ratio. The data crystal was a yellow needle of approximate dimensions 0.10 mm × 0.05 mm × 0.01 mm. The data crystal was an orange needle of approximate dimensions 0.182 mm × 0.021 mm × 0.014 mm. A single crystal of **2c**·Cl^–^-TBA^+^ was obtained by vapor diffusion of *n*-octane into a CHCl_3_ solution of the mixture of **2c** and TBACl in the 1:1 ratio. A single crystal of **2b**·Cl^–^-TPPAu^+^ was obtained by vapor diffusion of *n*-hexane into a THF solution of the mixture of **2b** and tetraphenylporphyrin Au^III^ complex as a Cl^–^ salt (TPPAuCl) [30] in the 1:1 ratio. The data crystal was a yellow needle of approximate dimensions 0.10 mm × 0.05 mm × 0.02 mm. A single crystal of **2c**·Cl^–^-TPPAu^+^ was obtained by vapor diffusion of *n*-hexane into an acetone solution of the mixture of **2c** and TPPAuCl [30] in the 1:1 ratio. The data crystal was an orange needle of approximate dimensions 0.30 mm × 0.30 mm × 0.05 mm. The data of **1c´** and **2b**·Cl^–^-TPPAu were collected at 90 K on a DECTRIS PILATUS3 CdTe 1 M diffractometer with Si (311) monochromated synchrotron radiation (λ = 0.4134 Å) at BL02B1 (SPring-8) [31], and those of **2b**·Cl^–^-TBA^+^, **2c**·Cl^–^-TBA^+^, and **2c**·Cl^–^-TPPAu^+^ were collected at 90, 100, and 90 K, respectively, on a DECTRIS EIGER X 1 M diffractometer with Si (111) monochromated synchrotron radiation (λ = 0.80977, 0.81070, and 0.81250 Å, respectively) at BL40XU (SPring-8) [32,33]. All the structures were solved by dual-space method. The structures were refined by a full-matrix least-squares method by using a SHELXL 2014 [34] (Yadokari-XG) [35,36]. In each structure, the non-hydrogen atoms were refined anisotropically. CIF files (CCDC-2326910–2326914) can be obtained free of charge from the Cambridge Crystallographic Data Centre via www.ccdc.cam.ac.uk/data_request/cif.

**Table 1.** Crystallographic details for **1c´**, **2b**·Cl^–^-TBA^+^, **2c**·Cl^–^-TBA^+^, **2b**·Cl^–^-TPPAu^+^, and **2c**·Cl^–^-TPPAu^+^.

	1c´	2b·Cl^–^-TBA^+^	2c·Cl^–^-TBA^+^	2b·Cl^–^-TPPAu^+^	2c·Cl^–^-TPPAu^+^
formula	C_35_H_32_F_2_N_2_O_2_	C_46_H_41_N_3_O_2_PtCl· C_16_H_36_N	C_46_H_39_N_3_O_2_F_2_PtCl· C_16_H_36_N	C_46_H_41_N_3_O_2_PtCl· C_44_H_28_AuN_4_	C_46_H_39_F_2_N_3_O_2_PtCl· C_44_H_28_AuN_4_·0.5C_3_H_6_O
fw	550.62	1140.81	1176.80	1708.02	1773.05
crystal size, mm	0.135 × 0.135 × 0.088	0.10 × 0.05 × 0.01	0.10 × 0.05 × 0.02	0.182 × 0.021 × 0.014	0.30 × 0.30 × 0.05
crystal system	triclinic	triclinic	triclinic	monoclinic	triclinic
space group	*P*-1 (no. 2)	*P*-1 (no. 2)	*P*-1 (no. 2)	*P*2_1_/c (no. 14)	*P*-1 (no. 2)
*a*, Å	13.939(13)	8.3650(12)	8.6106(4)	21.253(11)	12.9662(9)
*b*, Å	15.653(15)	15.407(3)	15.5274(8)	13.828(7)	13.3402(9)
*c*, Å	22.30(2)	43.844(5)	21.8913(9)	26.237(13)	22.4086(18)
*α*, °	91.48(2)	81.485(8)	103.845(4)	90	105.8934(15)
*β*, °	90.724(14)	85.275(9)	93.341(4)	108.162(8)	105.4630(15)
*γ*, °	114.070(11)	86.419(13)	97.512(4)	90	94.294(2)
*V*, Å^3^	4440(7)	5562.1(14)	2805.2(2)	7326(6)	3546.9(4)
*ρ*_calcd_, gcm^−3^	1.235	1.362	1.393	1.549	1.660
*Z*	6	4	2	4	2
*T*, K	90(2)	90(2)	100(2)	90(2)	90(2)
*μ*, mm^−1^	0.036^*a*^	3.623^*a*^	3.633^*a*^	0.989^a^	5.744^a^
no. of reflns	91446	52768	33930	199572	38823
no. of unique reflns	19154	20112	12424	16266	12978
variables	1209	1253	535	917	958
*λ*, Å	0.4134^*a*^	0.80977^*a*^	0.81250^*a*^	0.4134^a^	0.81070^a^
*R*_1_ (*I* > 2*σ*(*I*))	0.0954	0.1126	0.1351	0.0506	0.0483
*wR*_2_ (*I* > 2*σ*(*I*))	0.2691	0.2614	0.3408	0.1088	0.1522
*GOF*	0.992	1.048	1.045	1.149	1.205

^*a*^Synchrotron radiation.

### 2.3. Computational method

Density functional theory (DFT) calculations of the geometrical optimizations were carried out using the *Gaussian 16* program [37].

### 2.4. Method for emission spectra, quantum yields, and emission lifetimes

Emission spectra and quantum yields were recorded on a Hitachi F-4500 fluorescence spectrometer and a Hamamatsu Quantum Yields Measurements System for Organic LED Materials C9920-02, respectively. Emission lifetimes were measured using a C7990S system (Hamamatsu Photonics) equipped with a 403-nm excitation laser, producing 62-ps pulses with a repetition rate of 100 kHz.

## 3. Results and discussions

### 3.1. Synthesis and characterization

**Figure 2.**
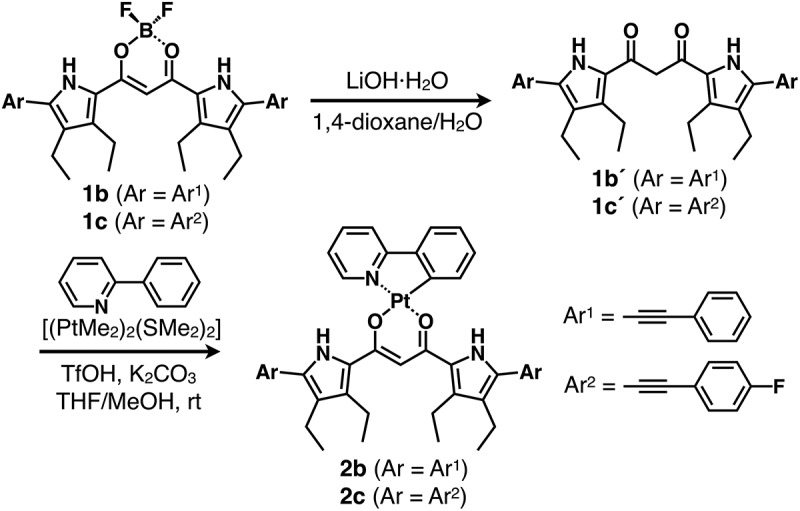
Synthesis of π-extended dipyrrolyldiketone Pt^II^ complexes **2b,c**.

**Figure 3.**
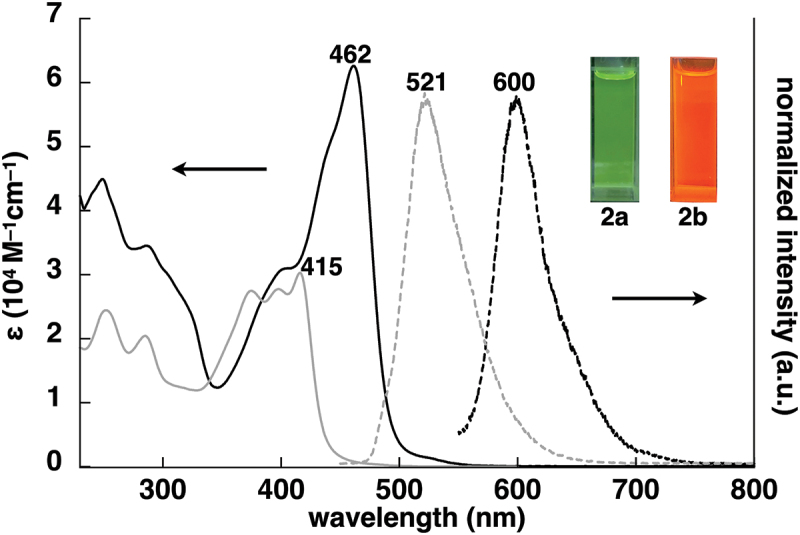
UV/vis absorption spectra (solid lines, CH_2_Cl_2_) and normalized emission spectra (dashed lines, deoxygenated CH_2_Cl_2_) with excitations at 415 and 462 nm for **2a** (gray) and **2b** (black), respectively (inset: photographs of **2a,b** under UV_365_ (0.02 mM)).

Dipyrrolyldiketone Pt^II^ complex **2a** was synthesized via Pt^II^ complexation of dipyrrolyldiketone **1a´** as a precursor to dipyrrolyldiketone BF_2_ complex **1a**. Arylethynyl-substituted dipyrrolyldiketones as key building blocks for the Pt^II^ complexes were prepared by removing a BF_2_ unit from the corresponding BF_2_ complexes using a modified method [26]. Treatment of α-arylethynyl dipyrrolyldiketone BF_2_ complexes **1b,c** [27,28] with LiOH·H_2_O in 1,4-dioxane/water followed by the addition of AcOH afforded dipyrrolyldiketones **1b´,c´** with yields of 83% and 87%, respectively. Pt^II^ complexes **2b,c** were prepared with yields of 15% and 32%, respectively, by treating **1b´,c´** with a mixture of arylpyridine and [(PtMe_2_)_2_(SMe_2_)_2_] [29] at r.t. in the presence of TfOH and K_2_CO_3_ (Figure 2) [38]. The obtained Pt^II^ complexes **2b,c** were characterized by ^1^H NMR and MALDI-TOF-MS. The complexes **2b,c** exhibited red-shifted absorption maxima (λ_max_) at 462 and 460 nm, respectively, in CH_2_Cl_2_ compared to unsubstituted **2a** (416 nm) (Figure 3 and S5) [24]. Time-dependent (TD)-DFT calculations [37] at PCM-B3LYP/6–31+G(d,p) with LanL2DZ for Pt (CH_2_Cl_2_) for the optimized structures revealed that the main absorption bands were mainly attributed to the highest occupied molecular orbital (HOMO)-to-lowest unoccupied molecular orbital (LUMO) transitions, with small contributions from the ligand-to-ligand charge transfer (LLCT) from the dipyrrolyldiketone unit to the arylpyridine ligand and the ligand-to-metal charge transfer (LMCT) (Figures S23 and S24). Furthermore, in deoxygenated CH_2_Cl_2_, **2b,c** exhibited red-shifted phosphorescence emissions at 600 and 598 nm, respectively, which were red-shifted by ~80 nm compared to that of **2a**, with quantum yields of 0.11 and 0.14, respectively (Figure S32). The emission lifetimes for **2b,c** were 17.3 and 8.0 μs, respectively (Figure S34), suggesting similar magnitude for previously reported dipyrrolyldiketone Pt^II^ complexes including **2a** [24,25]. Although dipyrrolyldiketone Pt^II^ complexes in the absence of pyrrole α-substituents, including **2a**, are stable under ambient conditions, π-extended Pt^II^ complexes **2b,c** showed degradation over 10 h when kept in solution, converting to the corresponding dipyrrolyldiketones **1b´,c´** by Pt^II^ demetallation. Therefore, a detailed evaluation of the solution-state electronic properties over a prolonged time could not be conducted.

### 3.2. Anion-binding behaviors

Despite slow degradation, the anion-binding behavior of the dipyrrolyldiketone Pt^II^ complexes in solution was preliminarily elucidated by anion-titration experiments via ^1^H NMR. Upon the addition of 1.3 equivalent of TBACl to **2b** in CD_2_Cl_2_ (1.0 mM) at −50°C, the ^1^H NMR signals of the pyrrole NH and bridging CH at 9.29/9.23 and 6.38 ppm were shifted downfield to 12.62 and 6.61 ppm, respectively, suggesting the formation of [1 + 1]-type Cl^–^ complexes as also observed for the dipyrrolyldiketone Pt^II^ complex **2a** (Figure S31) [24,25]. It should be noted that the [2 + 1]-type anion-binding mode, seen in arylethynyl-substituted BF_2_ complexes including **1b** [27,28], was not observed. This can be attributed to the smaller anion-binding cavity for **2b**·Cl^–^ than **1b**·Cl^–^, as suggested by theoretically optimized structures [37]. The bridging ∠C–C–C angle in the diketone unit of the optimized **2b**·Cl^–^ was 128.1°, which is larger by 9.1° than that of **1b**·Cl^–^, suggesting that the larger Pt^II^ induced the larger ∠ C–C–C angle and resulting smaller anion-binding cavity (Figure S19). The formation of the [1 + 1]-type Cl^–^ binding mode was also evaluated using UV/vis absorption spectral changes upon the addition of TBACl in CH_2_Cl_2_ (2.0 × 10^−5^ M) (Figure S30). The absorbance at the λ_max_ of 462 and 460 nm for **2a,b**, respectively, decreased upon the addition of TBACl, suggesting the inversion of arylethynyl-substituted pyrrole units upon Cl^–^ binding. A significant decrease in the λ_max_ absorbances was observed in the corresponding BF_2_ complexes **1b,c** [27,39].

### 3.3. Solid-state ion-pairing structures

**Figure 4.**
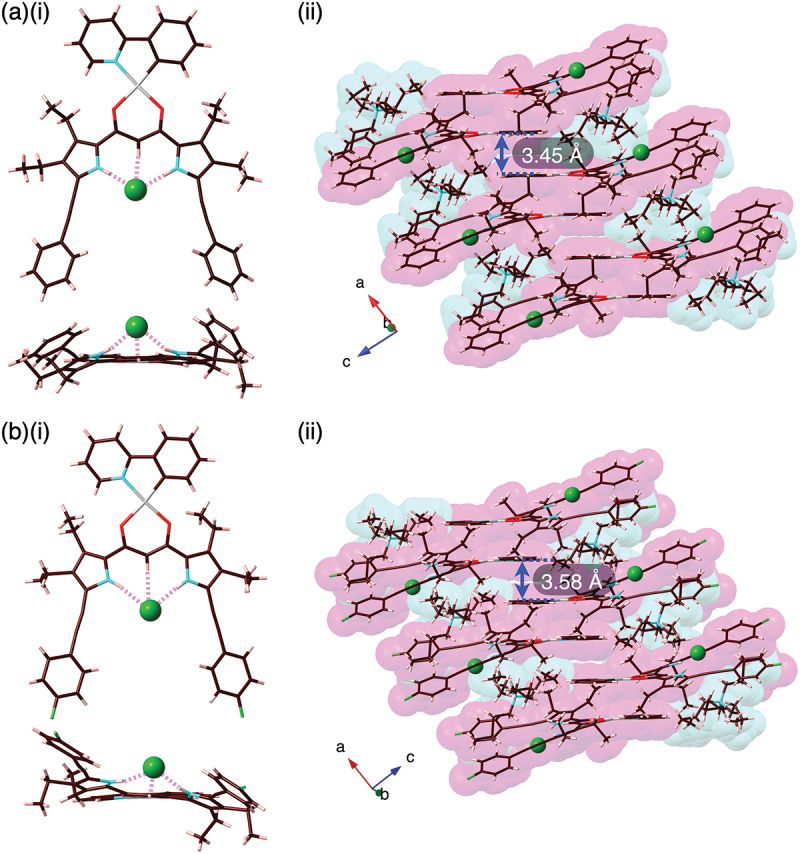
Single-crystal X-ray analysis of (a) **2b**·Cl^–^-TBA^+^ and (b) **2c**·Cl^–^-TBA^+^ ((i) top and side views and (ii) packing diagrams). Atom color code in Figure 4 and the following figures: brown, pink, blue, red, yellow green, green (spherical), and gray refer to carbon, hydrogen, nitrogen, oxygen, fluorine, chlorine, and platinum, respectively.

Solid-state ion-pairing assemblies of receptor–anion complexes and countercations were revealed by X-ray analysis of single crystals. The single crystals of **2b**·Cl^–^-TBA^+^ and **2c**·Cl^–^-TBA^+^ were prepared by vapor diffusion of acetone/*n*-hexane and CHCl_3_/*n*-octane, respectively, for the mixed solutions of the Pt^II^ complexes and TBACl. Single-crystal X-ray analysis of **2b**·Cl^–^-TBA^+^ and **2c**·Cl^–^-TBA^+^ revealed the [1 + 1]-type Cl^–^-binding mode using pyrrole N–H···Cl^–^ and bridging C–H···Cl^–^ hydrogen-bonding interactions with the N/C(–H)···Cl^–^ distances of 3.18/3.19 and 3.60 Å (for an independent structure) and 3.28/3.17 and 3.60 Å, respectively (Figure 4, S11, and S12). The planar Cl^–^ complexes **2b**·Cl^–^ and **2c**·Cl^–^, showing mean-plane deviations of 0.37/0.49 (two independent structures) and 0.35 Å, respectively, for the planes consisting of arylethynyl-substituted dipyrrolyldiketones and Cl^–^, were alternately arranged with counter TBA^+^, forming charge-by-charge columnar structures. The proximally located Pt^II^···Pt^II^ distance in **2b**·Cl^–^-TBA^+^ and **2c**·Cl^–^-TBA^+^ were 6.73 and 5.78 Å, respectively, indicating the absence of favorable Pt^II^···Pt^II^ interactions. In both cases, TBA^+^ was positioned in close proximity to the dipyrrolyldiketone–Cl^–^ complex unit. Consequently, the phenylpyridine unit of the Pt^II^ complex was partially stacked with the distances measuring 3.45 and 3.58 Å, respectively. The nearly flat geometries of the square planar Pt^II^ complexes were also indicated by τ_4_ values [40] of 0.08/0.09 and 0.09 for **2b**·Cl^–^ and **2c**·Cl^–^, respectively.

**Figure 5.**
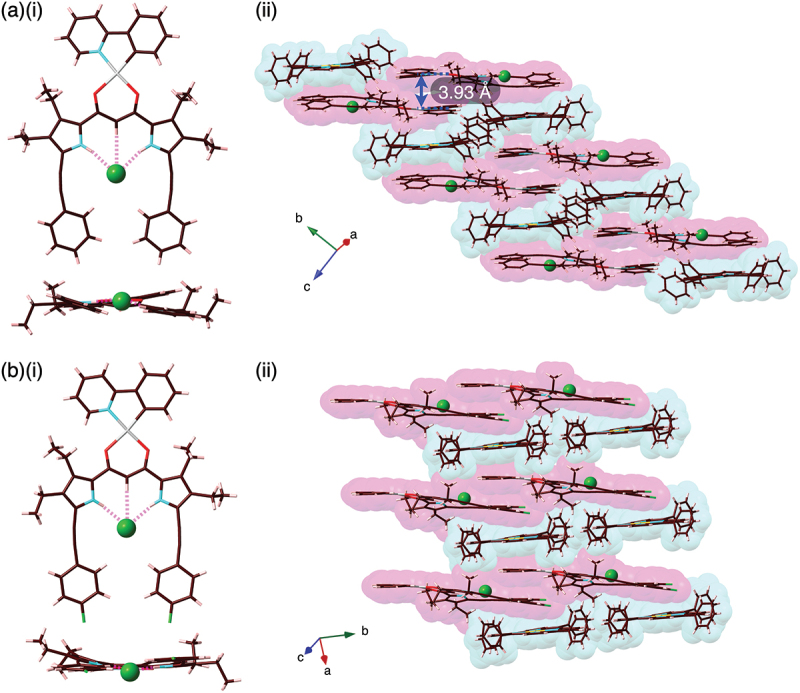
Single-crystal X-ray analysis of (a) **2b**·Cl^–^-TPPAu^+^ and (b) **2c**·Cl^–^-TPPAu^+^ ((i) top and side views and (ii) packing diagrams).

**Figure 6.**
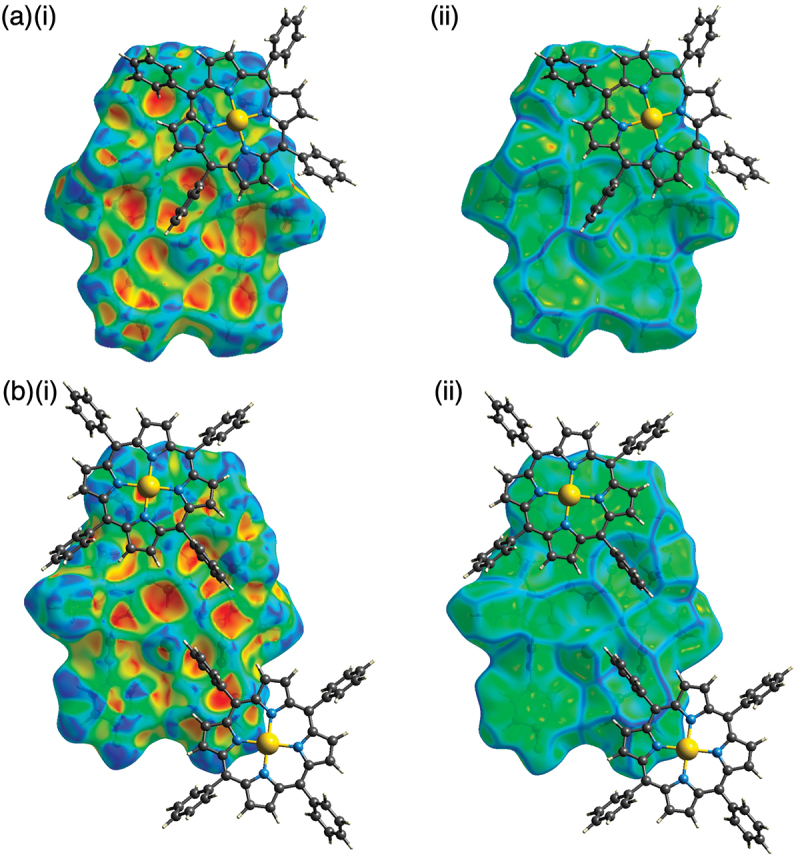
Hirshfeld surface analysis of (a) **2b**·Cl^–^-TPPAu^+^ and (b) **2c**·Cl^–^-TPPAu^+^ mapped over (i) shape-index and (ii) curvedness properties for selected stacked structures.

Ion-pairing assemblies with π-electronic cations have also been investigated using single-crystal X-ray analysis. Single crystals of **2b**·Cl^–^-TPPAu^+^ and **2c**·Cl^–^-TPPAu^+^ (TPPAu^+^: *meso*-tetraphenylporphyrin Au^III^ complex [30]) were prepared by vapor diffusion of THF/*n*-hexane and acetone/*n*-hexane, respectively. TPPAu^+^ has been used as a planar π-electronic cation for ion-pairing assemblies with anion-responsive π-electronic molecules in their anion complex forms [19,24,41]. In the solid state, **2b**·Cl^–^ and **2c**·Cl^–^ formed planar [1 + 1]-type Cl^–^-binding structures with hydrogen bonding with the pyrrole-N(–H)···Cl^–^ and bridging-C(–H)···Cl^–^ distances of 3.19/3.18 and 3.68 Å and 3.09/3.12 and 3.60 Å, respectively (Figure 5, S13, and S14). Mean-plane deviations for **2b**·Cl^–^ and **2c**·Cl^–^ are 0.20 and 0.28 Å, respectively, which are smaller than those in the ion-pairing assemblies with TBA^+^. The smaller mean-plane deviations are attributed to the stacking of the planar anion complexes and TPPAu^+^. In fact, the distances between the mean planes of the Cl^–^ complexes and TPPAu^+^ are 3.91 and 3.61 Å, respectively. Planar units of the phenylpyridine–Pt^II^ units are also stacked with TPPAu^+^ with the stacking distances of 3.65 and 3.26 Å, respectively, forming charge-by-charge stacking columnar structures. It should be noticed that the planes comprising the phenylpyridine and dipyrrolyldiketone–Cl^–^ complex units were independently stacked with the proximally located TPPAu^+^. The Hirshfeld surface analysis [42] of the stacking structures of phenylpyridine and TPPAu^+^ showed red and blue triangles arranged in bow-tie shapes on the shape-index surface and a flat region on the curvedness, suggesting characteristic mapping patterns for π–π stacking structures (Figure 6, S17, and S18).

**Figure 7.**
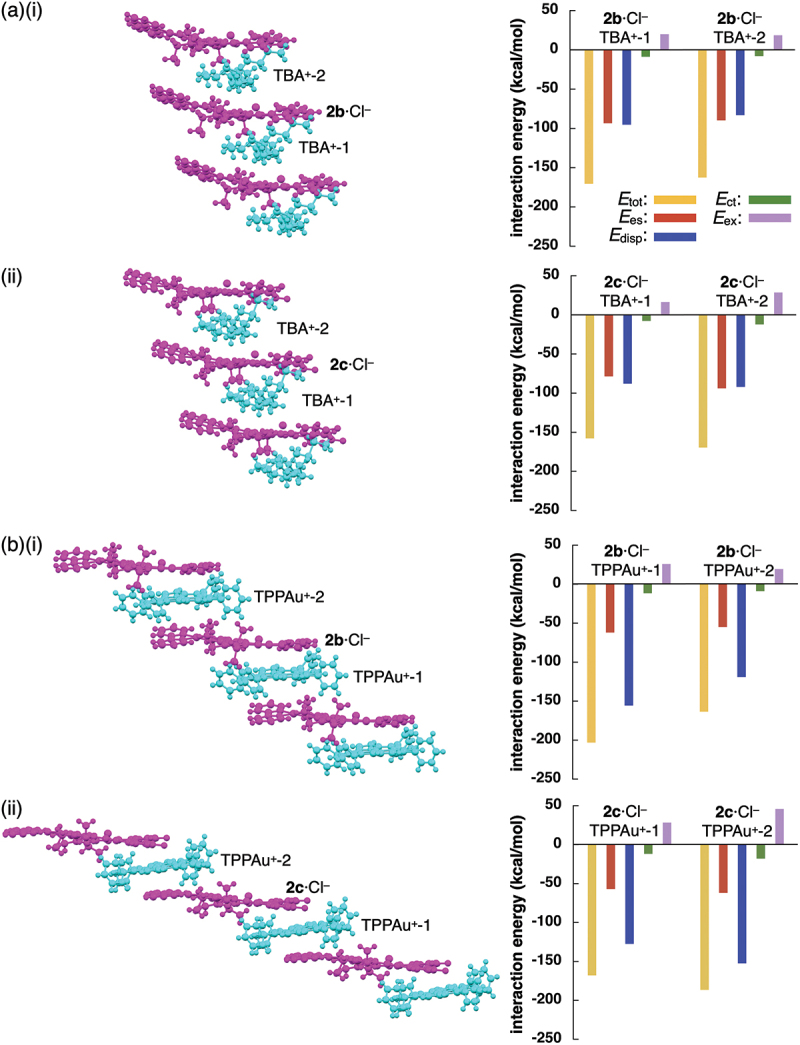
Energy decomposition analysis (EDA) of single-crystal X-ray structures: (a)(i) **2b**·Cl^–^-TBA^+^ and (ii) **2c**·Cl^–^-TPPAu^+^ and (b)(i) **2b**·Cl^–^ TPPAu and (ii) **2c**·Cl^–^-TPPAu^+^ (left: packing structures and right: interaction energies for proximally located ion pairs). The anion and cation parts are represented in magenta and cyan colors, respectively.

The charge-by-charge assemblies of planar [1 + 1]-type anion complexes as pseudo-π-electronic anions and countercations were further analyzed to determine the interaction energies between the components. Energy decomposition analysis (EDA) based on an FMO2-MP2 [43–47] using mixed basis sets including NOSeC-V-TZP for Pt and NOSeC-V-DZP for the other atoms for stacked structure of **2b**·Cl^–^ and TPPAu^+^ suggested total interaction energy (*E*_tot_) of −203.1 kcal/mol with contributions of electrostatic (*E*_es_) and dispersion (*E*_disp_) interaction energies of −61.9 and −155.6 kcal/mol (for the larger *E*_tot_ ion pair), respectively, whereas the *E*_tot_, *E*_es_, and *E*_disp_ of the proximally located **2b**·Cl^–^ and TBA^+^ were −170.5, −86.3, and −95.2 kcal/mol, respectively (Figure 7(a)). The larger absolute value of *E*_tot_ for **2b**·Cl^–^-TPPAu^+^ than **2b**·Cl^–^-TBA^+^ is mainly attributed to the larger *E*_disp_ value, suggesting the occurrence of effective ^*i*^π–^*i*^π interactions between stacked **2b**·Cl^–^ and TPPAu^+^ [48]. Similarly, the larger *E*_disp_ for **2c**·Cl^–^-TPPAu^+^ than **2c**·Cl^–^-TBA^+^ resulted in the larger *E*_tot_ value (Figure 7(b)). In contrast to dipyrrolyldiketone boron complexes [49], the charge-by-charge assemblies of anion-responsive π-electronic systems with π-electronic cations through ^*i*^π–^*i*^π interactions were limited to the Pt^II^ complexes [24,25]. π-Extension of the dipyrrolyldiketone Pt^II^ complexes resulted in the formation of larger π-electronic anions, which can be used as the versatile building units in ion-pairing assemblies.

## 4. Conclusions

Arylethynyl-substituted dipyrrolyldiketone Pt^II^ complexes, as anion-responsive π-electronic systems, exhibit red-shifted absorption and photoluminescence properties. Single-crystal X-ray analysis revealed charge-by-charge assemblies of anion complexes and countercations. In particular, ion-pairing assemblies with π-electronic cation form effective stacked structures via ^*i*^π–^*i*^π interactions. The stacking of phenylpyridine as a π-electronic ligand for Pt^II^ complexes is also important in ion-pairing assemblies. Further modifications through π-extension and the incorporation of chiral units would lead to the development of phosphorescent ion-pairing materials with chiroptical properties.

## Supplementary Material

Supplemental Material

Supplemental Material

Supplemental Material
