# Supplementary material for: Ion-pairing assemblies of π-extended anion-responsive organoplatinum complexes
Source: Sci Technol Adv Mater. 2024 Feb 6;25(1):2313958. doi: 10.1080/14686996.2024.2313958 (PMC10898271; doi:10.1080/14686996.2024.2313958)

checkCIF/PLATON report

Structure factors have been supplied for datablock(s) ymu-20-1

THIS REPORT IS FOR GUIDANCE ONLY. IF USED AS PART OF A REVIEW PROCEDURE FOR PUBLICATION, IT SHOULD NOT REPLACE THE EXPERTISE OF AN EXPERIENCED CRYSTALLOGRAPHIC REFEREE.

No syntax errors found. CIF dictionary Interpreting this report

Datablock: ymu-20-1

```
Bond precision:   C-C = 0.0037 A          Wavelength=0.41340

Cell:           a=13.939(13)     b=15.653(15)      c=22.30(2)
                alpha=91.48(2)   beta=90.724(14)   gamma=114.070(11)
Temperature:    100 K
```

	Calculated	Reported
Volume	4440(7)	4440(7)
Space group	P -1	P -1
Hall group	-P 1	-P 1
Moiety formula	C35 H32 F2 N2 O2	C35 H32 F2 N2 O2
Sum formula	C35 H32 F2 N2 O2	C35 H32 F2 N2 O2
Mr	550.63	550.62
Dx,g cm-3	1.236	1.235
Z	6	6
Mu (mm-1)	0.036	0.036
F000	1740.0	1740.0
F000'	1739.80	
h,k,lmax	17,20,28	17,19,28
Nref	19451	19154
Tmin,Tmax	0.995,0.997	0.543,1.000
Tmin'	0.995	

```
Correction method= # Reported T Limits: Tmin=0.543 Tmax=1.000
AbsCorr = MULTI-SCAN

Data completeness= 0.985           Theta(max)= 15.330

R(reflections)= 0.0954( 13564)                  wR2(reflections)=
                                                0.2923( 19154)
S = 0.992                  Npar= 1209
```

The following ALERTS were generated. Each ALERT has the format
test-name_ALERT_alert-type_alert-level.
Click on the hyperlinks for more details of the test.

Alert level B

```
DIFMN02_ALERT_2_B  The minimum difference density is < -0.1*ZMAX*1.00
            _refine_diff_density_min given =     -1.120
            Test value =     -0.900
PLAT098_ALERT_2_B Large Reported Min.  (Negative) Residual Density      -1.12 eA-3
PLAT230_ALERT_2_B Hirshfeld Test Diff for    F4       --C024     .       24.2 s.u.
PLAT230_ALERT_2_B Hirshfeld Test Diff for    F6       --C032     .       19.0 s.u.
PLAT230_ALERT_2_B Hirshfeld Test Diff for    F3       --C02K     .       22.2 s.u.
PLAT230_ALERT_2_B Hirshfeld Test Diff for    F8       --C031     .       14.4 s.u.
PLAT230_ALERT_2_B Hirshfeld Test Diff for    F5       --C02H     .       21.7 s.u.
PLAT230_ALERT_2_B Hirshfeld Test Diff for    F7       --C02T     .       18.4 s.u.
PLAT242_ALERT_2_B Low    'MainMol' Ueq as Compared to Neighbors of       C024 Check
PLAT242_ALERT_2_B Low    'MainMol' Ueq as Compared to Neighbors of       C032 Check
PLAT242_ALERT_2_B Low    'MainMol' Ueq as Compared to Neighbors of       C02K Check
PLAT242_ALERT_2_B Low    'MainMol' Ueq as Compared to Neighbors of       C031 Check
PLAT242_ALERT_2_B Low    'MainMol' Ueq as Compared to Neighbors of       C02H Check
PLAT242_ALERT_2_B Low    'MainMol' Ueq as Compared to Neighbors of       C02T Check
PLAT355_ALERT_3_B Long   O-H (X0.82,N0.98A)  O005     - H107     .       1.10 Ang.
```

Alert level C

```
DIFMN03_ALERT_1_C  The minimum difference density is < -0.1*ZMAX*0.75
            The relevant atom site should be identified.
RINTA01_ALERT_3_C  The value of Rint is greater than 0.12
            Rint given   0.170
PLAT020_ALERT_3_C The Value of Rint is Greater Than 0.12 .........      0.170 Report
PLAT084_ALERT_3_C High wR2 Value (i.e. > 0.25) ...................       0.29 Report
PLAT355_ALERT_3_C Long   O-H (X0.82,N0.98A)  O003     - H109     .       1.07 Ang.
PLAT355_ALERT_3_C Long   O-H (X0.82,N0.98A)  O006     - H108     .       1.06 Ang.
PLAT410_ALERT_2_C Short Intra H...H Contact  H7       ..H12      .       1.99 Ang.
                                             x,y,z  =      1_555 Check
PLAT420_ALERT_2_C D-H Bond Without Acceptor  N008     --H2       .     Please Check
PLAT420_ALERT_2_C D-H Bond Without Acceptor  N00B     --H5       .     Please Check
PLAT420_ALERT_2_C D-H Bond Without Acceptor  N00C     --H6       .     Please Check
PLAT790_ALERT_4_C Centre of Gravity not Within Unit Cell: Resd.  #          1 Note
              C35 H32 F2 N2 O2
PLAT906_ALERT_3_C Large K Value in the Analysis of Variance ......      4.733 Check
PLAT911_ALERT_3_C Missing FCF Refl Between Thmin & STh/L=    0.600          8 Report
PLAT918_ALERT_3_C Reflection(s) with I(obs) much Smaller I(calc) .          6 Check
```

Alert level G

```
ABSMU01_ALERT_1_G  Calculation of _exptl_absorpt_correction_mu
                not performed for this radiation type.
PLAT007_ALERT_5_G Number of Unrefined Donor-H Atoms ..............          7 Report
PLAT092_ALERT_4_G Check: Wavelength Given is not Cu,Ga,Mo,Ag,In Ka    0.41340 Ang.
PLAT230_ALERT_2_G Hirshfeld Test Diff for    C01S     --C020     .        6.3 s.u.
PLAT301_ALERT_3_G Main Residue  Disorder ..............(Resd  1  )        20% Note
PLAT309_ALERT_2_G Single Bonded Oxygen (C-O > 1.3 Ang) ..........       O004 Check
PLAT309_ALERT_2_G Single Bonded Oxygen (C-O > 1.3 Ang) ..........         O7 Check
```

```
PLAT309_ALERT_2_G Single Bonded Oxygen (C-O > 1.3 Ang) ..........       O002 Check
PLAT333_ALERT_2_G Large Aver C6-Ring C-C Dist C00S     -C027     .       1.42 Ang.
PLAT333_ALERT_2_G Large Aver C6-Ring C-C Dist C01S     -C02O     .       1.42 Ang.
PLAT333_ALERT_2_G Large Aver C6-Ring C-C Dist C017     -C02F     .       1.42 Ang.
PLAT333_ALERT_2_G Large Aver C6-Ring C-C Dist C025     -C02Y     .       1.42 Ang.
PLAT333_ALERT_2_G Large Aver C6-Ring C-C Dist C011     -C01U     .       1.42 Ang.
PLAT333_ALERT_2_G Large Aver C6-Ring C-C Dist C01D     -C02G     .       1.42 Ang.
PLAT371_ALERT_2_G Long   C(sp2)-C(sp1) Bond  C00R     - C44      .       1.45 Ang.
PLAT371_ALERT_2_G Long   C(sp2)-C(sp1) Bond  C00S     - C45      .       1.46 Ang.
PLAT371_ALERT_2_G Long   C(sp2)-C(sp1) Bond  C01S     - C020     .       1.46 Ang.
PLAT371_ALERT_2_G Long   C(sp2)-C(sp1) Bond  C012     - C41      .       1.46 Ang.
PLAT371_ALERT_2_G Long   C(sp2)-C(sp1) Bond  C013     - C40      .       1.45 Ang.
PLAT371_ALERT_2_G Long   C(sp2)-C(sp1) Bond  C017     - C01H     .       1.47 Ang.
PLAT371_ALERT_2_G Long   C(sp2)-C(sp1) Bond  C023     - C025     .       1.47 Ang.
PLAT371_ALERT_2_G Long   C(sp2)-C(sp1) Bond  C011     - C01C     .       1.46 Ang.
PLAT371_ALERT_2_G Long   C(sp2)-C(sp1) Bond  C01A     - C42      .       1.45 Ang.
PLAT371_ALERT_2_G Long   C(sp2)-C(sp1) Bond  C01D     - C01R     .       1.47 Ang.
PLAT371_ALERT_2_G Long   C(sp2)-C(sp1) Bond  C01E     - C43      .       1.45 Ang.
PLAT410_ALERT_2_G Short Intra H...H Contact  H8       ..H87      .       2.12 Ang.
                                                      x,y,z  =     1_555 Check
PLAT410_ALERT_2_G Short Intra H...H Contact  H8       ..H104     .       2.05 Ang.
                                                      x,y,z  =     1_555 Check
PLAT432_ALERT_2_G Short Inter X...Y Contact  F6       ..C49      .       2.90 Ang.
                                                    x,y,1+z  =     1_556 Check
PLAT434_ALERT_2_G Short Inter HL..HL Contact F4       ..F5       .       2.68 Ang.
                                                      x,y,z  =     1_555 Check
PLAT720_ALERT_4_G Number of Unusual/Non-Standard Labels .........        109 Note
PLAT883_ALERT_1_G No Info/Value for _atom_sites_solution_primary .     Please Do !
PLAT910_ALERT_3_G Missing # of FCF Reflection(s) Below Theta(Min).         1 Note
PLAT912_ALERT_4_G Missing # of FCF Reflections Above STh/L=  0.600       287 Note
PLAT941_ALERT_3_G Average HKL Measurement Multiplicity ..........        4.8 Low
PLAT978_ALERT_2_G Number C-C Bonds with Positive Residual Density.         1 Info
```

```
   0 ALERT level A = Most likely a serious problem - resolve or explain
  15 ALERT level B = A potentially serious problem, consider carefully
  14 ALERT level C = Check. Ensure it is not caused by an omission or oversight
  35 ALERT level G = General information/check it is not something unexpected

   3 ALERT type 1 CIF construction/syntax error, inconsistent or missing data
  44 ALERT type 2 Indicator that the structure model may be wrong or deficient
  12 ALERT type 3 Indicator that the structure quality may be low
   4 ALERT type 4 Improvement, methodology, query or suggestion
   1 ALERT type 5 Informative message, check
```

Validation response form

Please find below a validation response form (VRF) that can be filled in and pasted into your CIF.

```
# start Validation Reply Form
_vrf_DIFMN02_ymu-20-1
;
PROBLEM: The minimum difference density is < -0.1*ZMAX*1.00
RESPONSE: ...
;
_vrf_DIFMN03_ymu-20-1
```

```
;
PROBLEM: The minimum difference density is < -0.1*ZMAX*0.75
RESPONSE: ...
;
_vrf_RINTA01_ymu-20-1
;
PROBLEM: The value of Rint is greater than 0.12
RESPONSE: ...
;
_vrf_PLAT098_ymu-20-1
;
PROBLEM: Large Reported Min.  (Negative) Residual Density      -1.12 eA-3
RESPONSE: ...
;
_vrf_PLAT230_ymu-20-1
;
PROBLEM: Hirshfeld Test Diff for    F4       --C024     .       24.2 s.u.
RESPONSE: ...
;
_vrf_PLAT242_ymu-20-1
;
PROBLEM: Low    'MainMol' Ueq as Compared to Neighbors of       C024 Check
RESPONSE: ...
;
_vrf_PLAT355_ymu-20-1
;
PROBLEM: Long   O-H (X0.82,N0.98A)  O005     - H107     .       1.10 Ang.
RESPONSE: ...
;
_vrf_PLAT020_ymu-20-1
;
PROBLEM: The Value of Rint is Greater Than 0.12 .........      0.170 Report
RESPONSE: ...
;
_vrf_PLAT084_ymu-20-1
;
PROBLEM: High wR2 Value (i.e. > 0.25) ..................       0.29 Report
RESPONSE: ...
;
_vrf_PLAT410_ymu-20-1
;
PROBLEM: Short Intra H...H Contact  H7       ..H12      .       1.99 Ang.
RESPONSE: ...
;
_vrf_PLAT420_ymu-20-1
;
PROBLEM: D-H Bond Without Acceptor  N008     --H2       .     Please Check
RESPONSE: ...
;
_vrf_PLAT790_ymu-20-1
;
PROBLEM: Centre of Gravity not Within Unit Cell: Resd.  #          1 Note
RESPONSE: ...
;
_vrf_PLAT906_ymu-20-1
;
PROBLEM: Large K Value in the Analysis of Variance ......      4.733 Check
```

```
RESPONSE: ...
;
_vrf_PLAT911_ymu-20-1
;
PROBLEM: Missing FCF Refl Between Thmin & STh/L=    0.600          8 Report
RESPONSE: ...
;
_vrf_PLAT918_ymu-20-1
;
PROBLEM: Reflection(s) with I(obs) much Smaller I(calc) .          6 Check
RESPONSE: ...
;
# end Validation Reply Form
```

It is advisable to attempt to resolve as many as possible of the alerts in all categories. Often the minor alerts point to easily fixed oversights, errors and omissions in your CIF or refinement strategy, so attention to these fine details can be worthwhile. In order to resolve some of the more serious problems it may be necessary to carry out additional measurements or structure refinements. However, the purpose of your study may justify the reported deviations and the more serious of these should normally be commented upon in the discussion or experimental section of a paper or in the "special_details" fields of the CIF. checkCIF was carefully designed to identify outliers and unusual parameters, but every test has its limitations and alerts that are not important in a particular case may appear. Conversely, the absence of alerts does not guarantee there are no aspects of the results needing attention. It is up to the individual to critically assess their own results and, if necessary, seek expert advice.

Publication of your CIF in IUCr journals

A basic structural check has been run on your CIF. These basic checks will be run on all CIFs submitted for publication in IUCr journals (*Acta Crystallographica*, *Journal of Applied Crystallography*, *Journal of Synchrotron Radiation*); however, if you intend to submit to *Acta Crystallographica Section C* or *E* or *IUCrData*, you should make sure that full publication checks are run on the final version of your CIF prior to submission.

Publication of your CIF in other journals

Please refer to the *Notes for Authors* of the relevant journal for any special instructions relating to CIF submission.

PLATON version of 12/09/2022; check.def file version of 09/08/2022

Datablock ymu-20-1 - ellipsoid plot

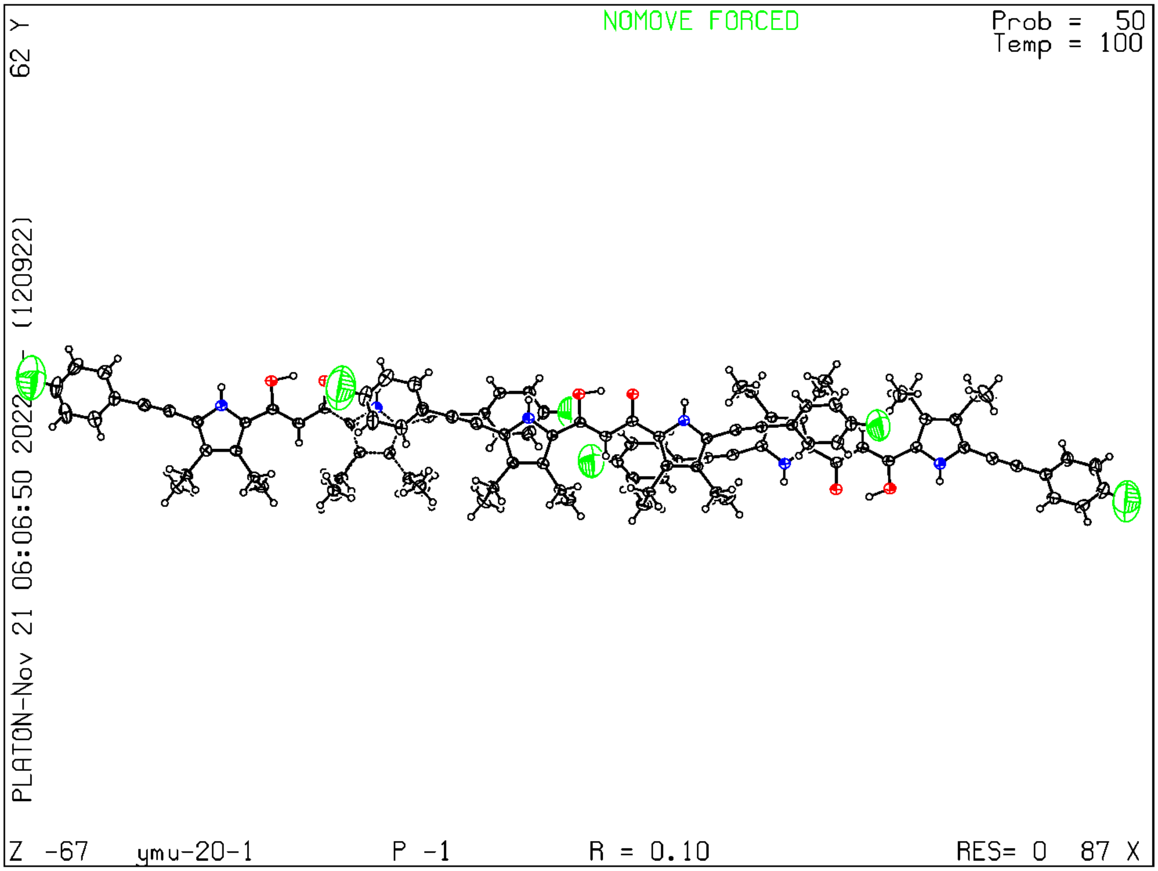

checkCIF/PLATON report

Structure factors have been supplied for datablock(s) ymu-23-2

THIS REPORT IS FOR GUIDANCE ONLY. IF USED AS PART OF A REVIEW PROCEDURE FOR PUBLICATION, IT SHOULD NOT REPLACE THE EXPERTISE OF AN EXPERIENCED CRYSTALLOGRAPHIC REFEREE.

No syntax errors found. CIF dictionary Interpreting this report

Datablock: ymu-23-2

Bond precision: C-C = 0.0285 A Wavelength=0.80977

Cell:	a=8.3650(12)	b=15.407(3)	c=43.844(5)
	alpha=81.485(8)	beta=85.275(9)	gamma=86.419(13)
Temperature:	90 K		

	Calculated	Reported
Volume	5562.1(15)	5562.1(14)
Space group	P -1	P -1
Hall group	-P 1	-P 1
Moiety formula	C46 H41 N3 O2 Pt, C16 H36 N, Cl	C46 H41 N3 O2 Pt, C16 H36 N, Cl
Sum formula	C62 H77 Cl N4 O2 Pt	C62 H77 Cl N4 O2 Pt
Mr	1140.81	1140.81
Dx,g cm-3	1.362	1.362
Z	4	4
Mu (mm-1)	3.623	3.623
F000	2352.0	2352.0
F000’	2340.83	
h,k,lmax	10,18,52	10,18,52
Nref	20356	20112
Tmin,Tmax	0.805,0.964	0.614,1.000
Tmin’	0.696	

Correction method= # Reported T Limits: Tmin=0.614 Tmax=1.000
AbsCorr = MULTI-SCAN

Data completeness= 0.988 Theta(max)= 29.197

R(reflections)= 0.1126(10514) wR2(reflections)= 0.3065(20112)

S = 1.048 Npar= 1253

The following ALERTS were generated. Each ALERT has the format
test-name_ALERT_alert-type_alert-level.
Click on the hyperlinks for more details of the test.

Alert level B

```
PLAT234_ALERT_4_B Large Hirshfeld Difference C22      --C23      .        0.26 Ang.
PLAT234_ALERT_4_B Large Hirshfeld Difference C99      --C100     .        0.28 Ang.
PLAT241_ALERT_2_B High   'MainMol' Ueq as Compared to Neighbors of          C2 Check
PLAT241_ALERT_2_B High   'MainMol' Ueq as Compared to Neighbors of         C73 Check
PLAT342_ALERT_3_B Low Bond Precision on  C-C Bonds ...............    0.02854 Ang.
PLAT971_ALERT_2_B Check Calcd Resid. Dens.  1.11Ang From Pt1              3.01 eA-3
PLAT971_ALERT_2_B Check Calcd Resid. Dens.  1.27Ang From Pt2              2.95 eA-3
```

Alert level C

```
SHFSU01_ALERT_2_C  The absolute value of parameter shift to su ratio > 0.05
            Absolute value of the parameter shift to su ratio given   0.061
            Additional refinement cycles may be required.
PLAT080_ALERT_2_C Maximum Shift/Error ............................        0.06 Why ?
PLAT082_ALERT_2_C High R1 Value ..................................        0.11 Report
PLAT084_ALERT_3_C High wR2 Value (i.e. > 0.25) ...................        0.31 Report
PLAT094_ALERT_2_C Ratio of Maximum / Minimum Residual Density ....        2.03 Report
PLAT213_ALERT_2_C Atom C37             has ADP max/min Ratio .....         3.9 oblate
PLAT234_ALERT_4_C Large Hirshfeld Difference O1       --C19      .        0.20 Ang.
PLAT234_ALERT_4_C Large Hirshfeld Difference N2       --C31      .        0.17 Ang.
PLAT234_ALERT_4_C Large Hirshfeld Difference N3       --C24      .        0.20 Ang.
PLAT234_ALERT_4_C Large Hirshfeld Difference C6       --C7       .        0.24 Ang.
PLAT234_ALERT_4_C Large Hirshfeld Difference C8       --C9       .        0.24 Ang.
PLAT234_ALERT_4_C Large Hirshfeld Difference C17      --C18      .        0.21 Ang.
PLAT234_ALERT_4_C Large Hirshfeld Difference C19      --C31      .        0.19 Ang.
PLAT234_ALERT_4_C Large Hirshfeld Difference C24      --C25      .        0.24 Ang.
PLAT234_ALERT_4_C Large Hirshfeld Difference C28      --C29      .        0.19 Ang.
PLAT234_ALERT_4_C Large Hirshfeld Difference C29      --C30      .        0.23 Ang.
PLAT234_ALERT_4_C Large Hirshfeld Difference C41      --C46      .        0.19 Ang.
PLAT234_ALERT_4_C Large Hirshfeld Difference C45      --C46      .        0.22 Ang.
PLAT234_ALERT_4_C Large Hirshfeld Difference N6       --C66      .        0.22 Ang.
PLAT234_ALERT_4_C Large Hirshfeld Difference C63      --C64      .        0.19 Ang.
PLAT234_ALERT_4_C Large Hirshfeld Difference C71      --C76      .        0.24 Ang.
PLAT234_ALERT_4_C Large Hirshfeld Difference C77      --C78      .        0.24 Ang.
PLAT234_ALERT_4_C Large Hirshfeld Difference N7       --C93      .        0.20 Ang.
PLAT234_ALERT_4_C Large Hirshfeld Difference C98      --C99      .        0.22 Ang.
PLAT234_ALERT_4_C Large Hirshfeld Difference C102     --C103     .        0.22 Ang.
PLAT241_ALERT_2_C High   'MainMol' Ueq as Compared to Neighbors of         C23 Check
PLAT241_ALERT_2_C High   'MainMol' Ueq as Compared to Neighbors of         C50 Check
PLAT241_ALERT_2_C High   'MainMol' Ueq as Compared to Neighbors of         C67 Check
PLAT241_ALERT_2_C High   'MainMol' Ueq as Compared to Neighbors of         C90 Check
PLAT241_ALERT_2_C High   'MainMol' Ueq as Compared to Neighbors of         C95 Check
PLAT241_ALERT_2_C High   'MainMol' Ueq as Compared to Neighbors of         C99 Check
PLAT241_ALERT_2_C High   'MainMol' Ueq as Compared to Neighbors of        C103 Check
PLAT241_ALERT_2_C High   'MainMol' Ueq as Compared to Neighbors of        C119 Check
PLAT241_ALERT_2_C High   'MainMol' Ueq as Compared to Neighbors of        C121 Check
PLAT242_ALERT_2_C Low    'MainMol' Ueq as Compared to Neighbors of          C1 Check
PLAT242_ALERT_2_C Low    'MainMol' Ueq as Compared to Neighbors of          C3 Check
PLAT242_ALERT_2_C Low    'MainMol' Ueq as Compared to Neighbors of         C13 Check
PLAT242_ALERT_2_C Low    'MainMol' Ueq as Compared to Neighbors of         C41 Check
```

```
PLAT242_ALERT_2_C Low    'MainMol' Ueq as Compared to Neighbors of        C52 Check
PLAT242_ALERT_2_C Low    'MainMol' Ueq as Compared to Neighbors of        C68 Check
PLAT242_ALERT_2_C Low    'MainMol' Ueq as Compared to Neighbors of        C72 Check
PLAT242_ALERT_2_C Low    'MainMol' Ueq as Compared to Neighbors of        C94 Check
PLAT242_ALERT_2_C Low    'MainMol' Ueq as Compared to Neighbors of         N8 Check
PLAT242_ALERT_2_C Low    'MainMol' Ueq as Compared to Neighbors of       C118 Check
PLAT250_ALERT_2_C Large U3/U1 Ratio for Average U(i,j) Tensor ....        2.1 Note
PLAT260_ALERT_2_C Large Average Ueq of Residue Including       Pt2      0.118 Check
PLAT260_ALERT_2_C Large Average Ueq of Residue Including        N7      0.116 Check
PLAT260_ALERT_2_C Large Average Ueq of Residue Including        N8      0.113 Check
PLAT414_ALERT_2_C Short Intra D-H..H-X       H5A      ..H64      .       1.99 Ang.
                                                  x,y,z  =      1_555 Check
PLAT906_ALERT_3_C Large K Value in the Analysis of Variance ......     29.438 Check
PLAT906_ALERT_3_C Large K Value in the Analysis of Variance ......      2.221 Check
PLAT906_ALERT_3_C Large K Value in the Analysis of Variance ......      6.709 Check
PLAT906_ALERT_3_C Large K Value in the Analysis of Variance ......      3.048 Check
PLAT906_ALERT_3_C Large K Value in the Analysis of Variance ......      2.039 Check
PLAT911_ALERT_3_C Missing FCF Refl Between Thmin & STh/L=    0.600        175 Report
PLAT973_ALERT_2_C Check Calcd Positive Resid. Density on       Pt1       1.24 eA-3
PLAT975_ALERT_2_C Check Calcd Resid. Dens.  1.09Ang From O4      .       0.78 eA-3
```

Alert level G

```
ABSMU01_ALERT_1_G  Calculation of _exptl_absorpt_correction_mu
                not performed for this radiation type.
PLAT003_ALERT_2_G Number of Uiso or Uij Restrained non-H Atoms ...         12 Report
PLAT007_ALERT_5_G Number of Unrefined Donor-H Atoms .............          4 Report
PLAT083_ALERT_2_G SHELXL Second Parameter in WGHT  Unusually Large      46.30 Why ?
PLAT092_ALERT_4_G Check: Wavelength Given is not Cu,Ga,Mo,Ag,In Ka    0.80977 Ang.
PLAT178_ALERT_4_G The CIF-Embedded .res File Contains SIMU Records          7 Report
PLAT232_ALERT_2_G Hirshfeld Test Diff (M-X)  Pt1      --O2       .        8.0 s.u.
PLAT232_ALERT_2_G Hirshfeld Test Diff (M-X)  Pt1      --C30      .        7.7 s.u.
PLAT371_ALERT_2_G Long   C(sp2)-C(sp1) Bond  C6       - C7       .       1.44 Ang.
PLAT371_ALERT_2_G Long   C(sp2)-C(sp1) Bond  C8       - C9       .       1.42 Ang.
PLAT371_ALERT_2_G Long   C(sp2)-C(sp1) Bond  C38      - C39      .       1.42 Ang.
PLAT371_ALERT_2_G Long   C(sp2)-C(sp1) Bond  C40      - C41      .       1.44 Ang.
PLAT371_ALERT_2_G Long   C(sp2)-C(sp1) Bond  C54      - C55      .       1.46 Ang.
PLAT371_ALERT_2_G Long   C(sp2)-C(sp1) Bond  C84      - C85      .       1.41 Ang.
PLAT371_ALERT_2_G Long   C(sp2)-C(sp1) Bond  C86      - C87      .       1.43 Ang.
PLAT794_ALERT_5_G Tentative Bond Valency for Pt1       (II)      .       2.28 Info
PLAT794_ALERT_5_G Tentative Bond Valency for Pt2       (II)      .       2.36 Info
PLAT860_ALERT_3_G Number of Least-Squares Restraints ............         42 Note
PLAT883_ALERT_1_G No Info/Value for _atom_sites_solution_primary .     Please Do !
PLAT910_ALERT_3_G Missing # of FCF Reflection(s) Below Theta(Min).          2 Note
PLAT912_ALERT_4_G Missing # of FCF Reflections Above STh/L=  0.600         68 Note
PLAT913_ALERT_3_G Missing # of Very Strong Reflections in FCF ....          1 Note
PLAT933_ALERT_2_G Number of HKL-OMIT Records in Embedded .res File          8 Note
PLAT941_ALERT_3_G Average HKL Measurement Multiplicity ...........        2.6 Low
PLAT965_ALERT_2_G The SHELXL WEIGHT Optimisation has not Converged     Please Check
PLAT978_ALERT_2_G Number C-C Bonds with Positive Residual Density.          0 Info
```

---

0 **ALERT level A** = Most likely a serious problem - resolve or explain
7 **ALERT level B** = A potentially serious problem, consider carefully
57 **ALERT level C** = Check. Ensure it is not caused by an omission or oversight
26 **ALERT level G** = General information/check it is not something unexpected

```
  2 ALERT type 1 CIF construction/syntax error, inconsistent or missing data
 49 ALERT type 2 Indicator that the structure model may be wrong or deficient
 12 ALERT type 3 Indicator that the structure quality may be low
 24 ALERT type 4 Improvement, methodology, query or suggestion
  3 ALERT type 5 Informative message, check
```

Validation response form

Please find below a validation response form (VRF) that can be filled in and pasted into your CIF.

```
# start Validation Reply Form
_vrf_SHFSU01_ymu-23-2
;
PROBLEM: The absolute value of parameter shift to su ratio > 0.05
RESPONSE: ...
;
_vrf_PLAT234_ymu-23-2
;
PROBLEM: Large Hirshfeld Difference C22      --C23      .       0.26 Ang.
RESPONSE: ...
;
_vrf_PLAT241_ymu-23-2
;
PROBLEM: High   'MainMol' Ueq as Compared to Neighbors of         C2 Check
RESPONSE: ...
;
_vrf_PLAT342_ymu-23-2
;
PROBLEM: Low Bond Precision on  C-C Bonds ..............    0.02854 Ang.
RESPONSE: ...
;
_vrf_PLAT971_ymu-23-2
;
PROBLEM: Check Calcd Resid. Dens.  1.11Ang From Pt1             3.01 eA-3
RESPONSE: ...
;
_vrf_PLAT080_ymu-23-2
;
PROBLEM: Maximum Shift/Error ...........................       0.06 Why ?
RESPONSE: ...
;
_vrf_PLAT082_ymu-23-2
;
PROBLEM: High R1 Value .................................       0.11 Report
RESPONSE: ...
;
_vrf_PLAT084_ymu-23-2
;
PROBLEM: High wR2 Value (i.e. > 0.25) ..................       0.31 Report
RESPONSE: ...
;
_vrf_PLAT094_ymu-23-2
;
PROBLEM: Ratio of Maximum / Minimum Residual Density ....       2.03 Report
RESPONSE: ...
```

```
;
_vrf_PLAT213_ymu-23-2
;
PROBLEM: Atom C37             has ADP max/min Ratio .....        3.9 oblate
RESPONSE: ...
;
_vrf_PLAT242_ymu-23-2
;
PROBLEM: Low    'MainMol' Ueq as Compared to Neighbors of         C1 Check
RESPONSE: ...
;
_vrf_PLAT250_ymu-23-2
;
PROBLEM: Large U3/U1 Ratio for Average U(i,j) Tensor ....        2.1 Note
RESPONSE: ...
;
_vrf_PLAT260_ymu-23-2
;
PROBLEM: Large Average Ueq of Residue Including       Pt2      0.118 Check
RESPONSE: ...
;
_vrf_PLAT414_ymu-23-2
;
PROBLEM: Short Intra D-H..H-X       H5A      ..H64      .       1.99 Ang.
RESPONSE: ...
;
_vrf_PLAT906_ymu-23-2
;
PROBLEM: Large K Value in the Analysis of Variance ......     29.438 Check
RESPONSE: ...
;
_vrf_PLAT911_ymu-23-2
;
PROBLEM: Missing FCF Refl Between Thmin & STh/L=    0.600        175 Report
RESPONSE: ...
;
_vrf_PLAT973_ymu-23-2
;
PROBLEM: Check Calcd Positive Resid. Density on       Pt1       1.24 eA-3
RESPONSE: ...
;
_vrf_PLAT975_ymu-23-2
;
PROBLEM: Check Calcd Resid. Dens.  1.09Ang From O4      .       0.78 eA-3
RESPONSE: ...
;
# end Validation Reply Form
```

It is advisable to attempt to resolve as many as possible of the alerts in all categories. Often the minor alerts point to easily fixed oversights, errors and omissions in your CIF or refinement strategy, so attention to these fine details can be worthwhile. In order to resolve some of the more serious problems it may be necessary to carry out additional measurements or structure refinements. However, the purpose of your study may justify the reported deviations and the more serious of these should normally be commented upon in the discussion or experimental section of a paper or in the "special_details" fields of the CIF. checkCIF was carefully designed to identify outliers and unusual parameters, but every test has its limitations and alerts that are not important in a particular case may appear. Conversely, the absence of alerts does not guarantee there are no aspects of the results needing attention. It is up to the individual to critically assess their own results and, if necessary, seek expert advice.

Publication of your CIF in IUCr journals

A basic structural check has been run on your CIF. These basic checks will be run on all CIFs submitted for publication in IUCr journals (*Acta Crystallographica*, *Journal of Applied Crystallography*, *Journal of Synchrotron Radiation*); however, if you intend to submit to *Acta Crystallographica Section C* or *E* or *IUCrData*, you should make sure that full publication checks are run on the final version of your CIF prior to submission.

Publication of your CIF in other journals

Please refer to the *Notes for Authors* of the relevant journal for any special instructions relating to CIF submission.

PLATON version of 18/05/2022; check.def file version of 17/05/2022

Datablock ymu-23-2 - ellipsoid plot

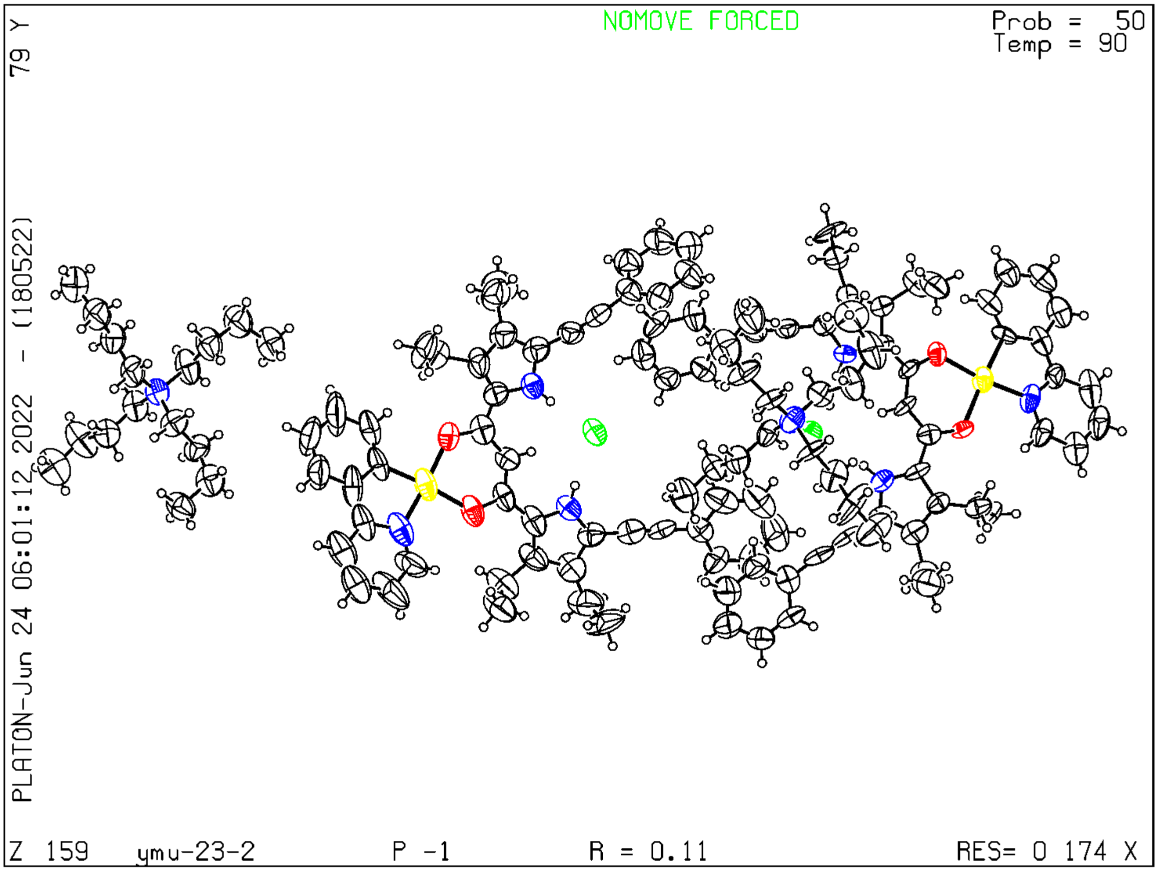

checkCIF/PLATON report

Structure factors have been supplied for datablock(s) 231116_ymu-44-1_a

THIS REPORT IS FOR GUIDANCE ONLY. IF USED AS PART OF A REVIEW PROCEDURE FOR PUBLICATION, IT SHOULD NOT REPLACE THE EXPERTISE OF AN EXPERIENCED CRYSTALLOGRAPHIC REFEREE.

No syntax errors found. CIF dictionary Interpreting this report

Datablock: 231116_ymu-44-1_a

Bond precision: C-C = 0.0346 A Wavelength=0.81250

Cell:	a=8.6106(4)	b=15.5274(8)	c=21.8913(9)
	alpha=103.845(4)	beta=93.341(4)	gamma=97.512(4)
Temperature:	100 K		

	Calculated	Reported
Volume	2805.2(2)	2805.2(2)
Space group	P -1	P -1
Hall group	-P 1	-P 1
Moiety formula	C92 H78 F4 N6 O4 Pt2, 2(C16 H36 N), 2(Cl)	C46 H39 F2 N3 O2 Pt, C16 H36 N, Cl
Sum formula	C124 H150 Cl2 F4 N8 O4 Pt2	C62 H75 Cl F2 N4 O2 Pt
Mr	2353.58	1176.80
Dx,g cm-3	1.393	1.393
Z	1	2
Mu (mm-1)	3.633	3.633
F000	1208.0	1208.0
F000’	1202.38	
h,k,lmax	11,20,29	10,20,29
Nref	14305	12424
Tmin,Tmax	0.804,0.930	0.768,1.000
Tmin’	0.695	

Correction method= # Reported T Limits: Tmin=0.768 Tmax=1.000
AbsCorr = MULTI-SCAN

Data completeness= 0.869 Theta(max)= 33.109

R(reflections)= 0.1351(5645) wR2(reflections)= 0.3974(12424)

S = 1.045 Npar= 535

The following ALERTS were generated. Each ALERT has the format
test-name_ALERT_alert-type_alert-level.
Click on the hyperlinks for more details of the test.

Alert level A

```
PLAT201_ALERT_2_A Isotropic non-H Atoms in Main Residue(s) .......         22 Report
              F2      N6      C12     C31     C41     C42     C43     C44
              C45     C46     C68     C72     C73     C74     C75     C76
              C77     C78     C80     C83     C87     C88
```

Author Response: Some atoms are solved as isotropic state due to disordering of the units.

Alert level B

```
PLAT031_ALERT_4_B Refined Extinction Parameter Within Range of ...      1.000 Sigma
PLAT084_ALERT_3_B High wR2 Value (i.e. > 0.25) ...................       0.40 Report
PLAT220_ALERT_2_B NonSolvent   Resd 1  C   Ueq(max)/Ueq(min) Range       10.0 Ratio
PLAT241_ALERT_2_B High   'MainMol' Ueq as Compared to Neighbors of        C24 Check
PLAT241_ALERT_2_B High   'MainMol' Ueq as Compared to Neighbors of        C91 Check
PLAT242_ALERT_2_B Low    'MainMol' Ueq as Compared to Neighbors of        C92 Check
PLAT342_ALERT_3_B Low Bond Precision on  C-C Bonds ...............    0.03459 Ang.
PLAT412_ALERT_2_B Short Intra XH3 .. XHn      H132      ..H134     .     1.74 Ang.
                                                      x,y,z  =      1_555 Check
PLAT972_ALERT_2_B Check Calcd Resid. Dens.  1.08Ang From Pt1            -2.60 eA-3
```

Alert level C

```
PLAT026_ALERT_3_C Ratio Observed / Unique Reflections (too) Low ..        45% Check
PLAT042_ALERT_1_C Calc. and Reported MoietyFormula Strings  Differ     Please Check
              Calc: C92 H78 F4 N6 O4 Pt2, 2(C16 H36 N), 2(Cl)
              Rep.: C46 H39 F2 N3 O2 Pt, C16 H36 N, Cl
PLAT082_ALERT_2_C High R1 Value .................................        0.14 Report
PLAT202_ALERT_3_C Isotropic non-H Atoms in Anion/Solvent .........          4 Check
              C89     C90     C91     C92
PLAT222_ALERT_3_C NonSolvent Resd 1  H   Uiso(max)/Uiso(min) Range       10.0 Ratio
PLAT234_ALERT_4_C Large Hirshfeld Difference C2       --C3       .       0.16 Ang.
PLAT234_ALERT_4_C Large Hirshfeld Difference C6       --C7       .       0.18 Ang.
PLAT241_ALERT_2_C High   'MainMol' Ueq as Compared to Neighbors of        C10 Check
PLAT241_ALERT_2_C High   'MainMol' Ueq as Compared to Neighbors of        C22 Check
PLAT241_ALERT_2_C High   'MainMol' Ueq as Compared to Neighbors of        C27 Check
PLAT241_ALERT_2_C High   'MainMol' Ueq as Compared to Neighbors of        C28 Check
PLAT241_ALERT_2_C High   'MainMol' Ueq as Compared to Neighbors of        C42 Check
PLAT241_ALERT_2_C High   'MainMol' Ueq as Compared to Neighbors of        C43 Check
PLAT241_ALERT_2_C High   'MainMol' Ueq as Compared to Neighbors of        C51 Check
PLAT242_ALERT_2_C Low    'MainMol' Ueq as Compared to Neighbors of         C3 Check
PLAT242_ALERT_2_C Low    'MainMol' Ueq as Compared to Neighbors of        C13 Check
PLAT242_ALERT_2_C Low    'MainMol' Ueq as Compared to Neighbors of        C17 Check
PLAT242_ALERT_2_C Low    'MainMol' Ueq as Compared to Neighbors of        C21 Check
PLAT242_ALERT_2_C Low    'MainMol' Ueq as Compared to Neighbors of        C25 Check
PLAT242_ALERT_2_C Low    'MainMol' Ueq as Compared to Neighbors of        C89 Check
PLAT245_ALERT_2_C U(iso) H19       Smaller than U(eq) C43       by      0.033 Ang**2
PLAT250_ALERT_2_C Large U3/U1 Ratio for Average U(i,j) Tensor ....        3.3 Note
```

```
PLAT260_ALERT_2_C Large Average Ueq of Residue Including       Pt1      0.156 Check
PLAT260_ALERT_2_C Large Average Ueq of Residue Including        N4      0.231 Check
PLAT260_ALERT_2_C Large Average Ueq of Residue Including       Cl1      0.135 Check
PLAT332_ALERT_2_C Large Phenyl C-C Range     C41       -C46      .        0.19 Ang.
PLAT360_ALERT_2_C Short  C(sp3)-C(sp3) Bond  C89      - C91      .        1.43 Ang.
PLAT361_ALERT_2_C Long   C(sp3)-C(sp3) Bond  C48      - C49      .        1.72 Ang.
PLAT410_ALERT_2_C Short Intra H...H Contact  H46      ..H136     .        1.94 Ang.
                                                      x,y,z  =      1_555 Check
PLAT412_ALERT_2_C Short Intra XH3 .. XHn     H67      ..H126     .        1.81 Ang.
                                                      x,y,z  =      1_555 Check
PLAT413_ALERT_2_C Short Inter XH3 .. XHn     H48      ..H104     .        2.09 Ang.
                                                 -x,1-y,1-z  =      2_566 Check
PLAT906_ALERT_3_C Large K Value in the Analysis of Variance ......     91.367 Check
PLAT906_ALERT_3_C Large K Value in the Analysis of Variance ......      2.825 Check
PLAT906_ALERT_3_C Large K Value in the Analysis of Variance ......     16.892 Check
PLAT906_ALERT_3_C Large K Value in the Analysis of Variance ......      2.512 Check
PLAT906_ALERT_3_C Large K Value in the Analysis of Variance ......      5.707 Check
PLAT906_ALERT_3_C Large K Value in the Analysis of Variance ......      2.718 Check
PLAT911_ALERT_3_C Missing FCF Refl Between Thmin & STh/L=    0.600         24 Report
                0 -2  1,   0  1  1,  -9  3  2,   5-14  3,   1 -7  3, -10  3  3,
               -9  3  3, -10  4  3,   5-17  4, -10  3  4,   1 11 12,   1 12 12,
                1 11 13,   1 12 13,   1 12 14,   5 -1 16,   5 -3 17,   6 -3 18,
               -5  1 18,  -5  1 19,  -5  1 20,   0-14 21,   0-13 21,   0-13 22,
PLAT971_ALERT_2_C Check Calcd Resid. Dens.  1.02Ang From Pt1            1.84 eA-3
PLAT973_ALERT_2_C Check Calcd Positive Resid. Density on       Pt1       1.20 eA-3
PLAT977_ALERT_2_C Check Negative Difference Density on H131      .      -0.45 eA-3
PLAT977_ALERT_2_C Check Negative Difference Density on H132      .      -0.53 eA-3
```

Alert level G

```
ABSMU01_ALERT_1_G  Calculation of _exptl_absorpt_correction_mu
                not performed for this radiation type.
PLAT002_ALERT_2_G Number of Distance or Angle Restraints on AtSite         22 Note
PLAT003_ALERT_2_G Number of Uiso or Uij Restrained non-H Atoms ...         20 Report
PLAT007_ALERT_5_G Number of Unrefined Donor-H Atoms .............           3 Report
              H5    H24   H15
PLAT045_ALERT_1_G Calculated and Reported Z Differ by a Factor ...      0.500 Check
PLAT083_ALERT_2_G SHELXL Second Parameter in WGHT  Unusually Large      19.34 Why ?
PLAT092_ALERT_4_G Check: Wavelength Given is not Cu,Ga,Mo,Ag,In Ka    0.81250 Ang.
PLAT093_ALERT_1_G No s.u.'s on H-positions, Refinement Reported as      mixed Check
PLAT154_ALERT_1_G The s.u.'s on the Cell Angles are Equal ..(Note)      0.004 Degree
PLAT171_ALERT_4_G The CIF-Embedded .res File Contains EADP Records          7 Report
PLAT172_ALERT_4_G The CIF-Embedded .res File Contains DFIX Records         10 Report
PLAT176_ALERT_4_G The CIF-Embedded .res File Contains SADI Records          1 Report
PLAT178_ALERT_4_G The CIF-Embedded .res File Contains SIMU Records          5 Report
PLAT186_ALERT_4_G The CIF-Embedded .res File Contains ISOR Records          1 Report
PLAT188_ALERT_3_G A Non-default SIMU Restraint Value has been used     0.0100 Report
PLAT188_ALERT_3_G A Non-default SIMU Restraint Value has been used     0.0100 Report
PLAT188_ALERT_3_G A Non-default SIMU Restraint Value has been used     0.0100 Report
PLAT188_ALERT_3_G A Non-default SIMU Restraint Value has been used     0.0100 Report
PLAT188_ALERT_3_G A Non-default SIMU Restraint Value has been used     0.0050 Report
PLAT301_ALERT_3_G Main Residue  Disorder .............(Resd  1  )         37% Note
PLAT371_ALERT_2_G Long   C(sp2)-C(sp1) Bond  C8       - C9       .        1.47 Ang.
PLAT410_ALERT_2_G Short Intra H...H Contact  H14      ..H99      .        1.88 Ang.
                                                      x,y,z  =      1_555 Check
PLAT413_ALERT_2_G Short Inter XH3 .. XHn     H132     ..H119     .        1.99 Ang.
                                                   -1+x,y,z  =      1_455 Check
```

```
PLAT414_ALERT_2_G Short Intra D-H..H-X       H6       ..H24      .       2.00 Ang.
                                                  x,y,z  =      1_555 Check
PLAT773_ALERT_2_G Check long C-C Bond in CIF: C48      --C49           1.71 Ang.
PLAT773_ALERT_2_G Check long C-C Bond in CIF: C65      --C66           2.02 Ang.
PLAT773_ALERT_2_G Check long C-C Bond in CIF: C66      --C65           2.02 Ang.
PLAT811_ALERT_5_G No ADDSYM Analysis: Too Many Excluded Atoms ....          ! Info
PLAT860_ALERT_3_G Number of Least-Squares Restraints ............       122 Note
PLAT883_ALERT_1_G No Info/Value for _atom_sites_solution_primary .     Please Do !
PLAT910_ALERT_3_G Missing # of FCF Reflection(s) Below Theta(Min).          1 Note
                0  0  1,
PLAT912_ALERT_4_G Missing # of FCF Reflections Above STh/L=  0.600       1678 Note
PLAT933_ALERT_2_G Number of HKL-OMIT Records in Embedded .res File          3 Note
                0  1  1,   0 -2  1,   1 -7  3,
PLAT941_ALERT_3_G Average HKL Measurement Multiplicity ..........        2.7 Low
PLAT965_ALERT_2_G The SHELXL WEIGHT Optimisation has not Converged     Please Check
PLAT978_ALERT_2_G Number C-C Bonds with Positive Residual Density.          3 Info
```

---

1 **ALERT level A** = Most likely a serious problem - resolve or explain
9 **ALERT level B** = A potentially serious problem, consider carefully
42 **ALERT level C** = Check. Ensure it is not caused by an omission or oversight
36 **ALERT level G** = General information/check it is not something unexpected

6 ALERT type 1 CIF construction/syntax error, inconsistent or missing data
49 ALERT type 2 Indicator that the structure model may be wrong or deficient
21 ALERT type 3 Indicator that the structure quality may be low
10 ALERT type 4 Improvement, methodology, query or suggestion
2 ALERT type 5 Informative message, check

It is advisable to attempt to resolve as many as possible of the alerts in all categories. Often the minor alerts point to easily fixed oversights, errors and omissions in your CIF or refinement strategy, so attention to these fine details can be worthwhile. In order to resolve some of the more serious problems it may be necessary to carry out additional measurements or structure refinements. However, the purpose of your study may justify the reported deviations and the more serious of these should normally be commented upon in the discussion or experimental section of a paper or in the "special_details" fields of the CIF. checkCIF was carefully designed to identify outliers and unusual parameters, but every test has its limitations and alerts that are not important in a particular case may appear. Conversely, the absence of alerts does not guarantee there are no aspects of the results needing attention. It is up to the individual to critically assess their own results and, if necessary, seek expert advice.

Publication of your CIF in IUCr journals

A basic structural check has been run on your CIF. These basic checks will be run on all CIFs submitted for publication in IUCr journals (*Acta Crystallographica*, *Journal of Applied Crystallography*, *Journal of Synchrotron Radiation*); however, if you intend to submit to *Acta Crystallographica Section C* or *E* or *IUCrData*, you should make sure that full publication checks are run on the final version of your CIF prior to submission.

Publication of your CIF in other journals

Please refer to the *Notes for Authors* of the relevant journal for any special instructions relating to CIF submission.

Validation response form

Please find below a validation response form (VRF) that can be filled in and pasted into your CIF.

```
# start Validation Reply Form
_vrf_PLAT031_231116_ymu-44-1_a
;
PROBLEM: Refined Extinction Parameter Within Range of ...      1.000 Sigma
RESPONSE: ...
;
_vrf_PLAT084_231116_ymu-44-1_a
;
PROBLEM: High wR2 Value (i.e. > 0.25) ...................       0.40 Report
RESPONSE: ...
;
_vrf_PLAT220_231116_ymu-44-1_a
;
PROBLEM: NonSolvent   Resd 1  C   Ueq(max)/Ueq(min) Range       10.0 Ratio
RESPONSE: ...
;
_vrf_PLAT241_231116_ymu-44-1_a
;
PROBLEM: High   'MainMol' Ueq as Compared to Neighbors of        C24 Check
RESPONSE: ...
;
_vrf_PLAT242_231116_ymu-44-1_a
```

```
;
PROBLEM: Low    'MainMol' Ueq as Compared to Neighbors of        C92 Check
RESPONSE: ...
;
_vrf_PLAT342_231116_ymu-44-1_a
;
PROBLEM: Low Bond Precision on  C-C Bonds ..............    0.03459 Ang.
RESPONSE: ...
;
_vrf_PLAT412_231116_ymu-44-1_a
;
PROBLEM: Short Intra XH3 .. XHn     H132     ..H134     .       1.74 Ang.
RESPONSE: ...
;
_vrf_PLAT972_231116_ymu-44-1_a
;
PROBLEM: Check Calcd Resid. Dens.  1.08Ang From Pt1            -2.60 eA-3
RESPONSE: ...
;
_vrf_PLAT026_231116_ymu-44-1_a
;
PROBLEM: Ratio Observed / Unique Reflections (too) Low ..        45% Check
RESPONSE: ...
;
_vrf_PLAT042_231116_ymu-44-1_a
;
PROBLEM: Calc. and Reported MoietyFormula Strings  Differ     Please Check
RESPONSE: ...
;
_vrf_PLAT082_231116_ymu-44-1_a
;
PROBLEM: High R1 Value ..................................       0.14 Report
RESPONSE: ...
;
_vrf_PLAT202_231116_ymu-44-1_a
;
PROBLEM: Isotropic non-H Atoms in Anion/Solvent .........          4 Check
RESPONSE: ...
;
_vrf_PLAT222_231116_ymu-44-1_a
;
PROBLEM: NonSolvent Resd 1  H   Uiso(max)/Uiso(min) Range       10.0 Ratio
RESPONSE: ...
;
_vrf_PLAT234_231116_ymu-44-1_a
;
PROBLEM: Large Hirshfeld Difference C2       --C3       .       0.16 Ang.
RESPONSE: ...
;
_vrf_PLAT245_231116_ymu-44-1_a
;
PROBLEM: U(iso) H19       Smaller than U(eq) C43       by      0.033 Ang**2
RESPONSE: ...
;
_vrf_PLAT250_231116_ymu-44-1_a
;
PROBLEM: Large U3/U1 Ratio for Average U(i,j) Tensor ....        3.3 Note
```

```
RESPONSE: ...
;
_vrf_PLAT260_231116_ymu-44-1_a
;
PROBLEM: Large Average Ueq of Residue Including       Pt1      0.156 Check
RESPONSE: ...
;
_vrf_PLAT332_231116_ymu-44-1_a
;
PROBLEM: Large Phenyl C-C Range     C41       -C46      .       0.19 Ang.
RESPONSE: ...
;
_vrf_PLAT360_231116_ymu-44-1_a
;
PROBLEM: Short  C(sp3)-C(sp3) Bond  C89      - C91      .       1.43 Ang.
RESPONSE: ...
;
_vrf_PLAT361_231116_ymu-44-1_a
;
PROBLEM: Long   C(sp3)-C(sp3) Bond  C48      - C49      .       1.72 Ang.
RESPONSE: ...
;
_vrf_PLAT410_231116_ymu-44-1_a
;
PROBLEM: Short Intra H...H Contact  H46      ..H136     .       1.94 Ang.
RESPONSE: ...
;
_vrf_PLAT413_231116_ymu-44-1_a
;
PROBLEM: Short Inter XH3 .. XHn     H48      ..H104     .       2.09 Ang.
RESPONSE: ...
;
_vrf_PLAT906_231116_ymu-44-1_a
;
PROBLEM: Large K Value in the Analysis of Variance ......     91.367 Check
RESPONSE: ...
;
_vrf_PLAT911_231116_ymu-44-1_a
;
PROBLEM: Missing FCF Refl Between Thmin & STh/L=    0.600         24 Report
RESPONSE: ...
;
_vrf_PLAT971_231116_ymu-44-1_a
;
PROBLEM: Check Calcd Resid. Dens.  1.02Ang From Pt1             1.84 eA-3
RESPONSE: ...
;
_vrf_PLAT973_231116_ymu-44-1_a
;
PROBLEM: Check Calcd Positive Resid. Density on       Pt1       1.20 eA-3
RESPONSE: ...
;
_vrf_PLAT977_231116_ymu-44-1_a
;
```

```
PROBLEM: Check Negative Difference Density on H131       .       -0.45 eA-3
RESPONSE: ...
;
# end Validation Reply Form
```

PLATON version of 29/11/2023; check.def file version of 14/09/2023

Datablock 231116_ymu-44-1_a - ellipsoid plot

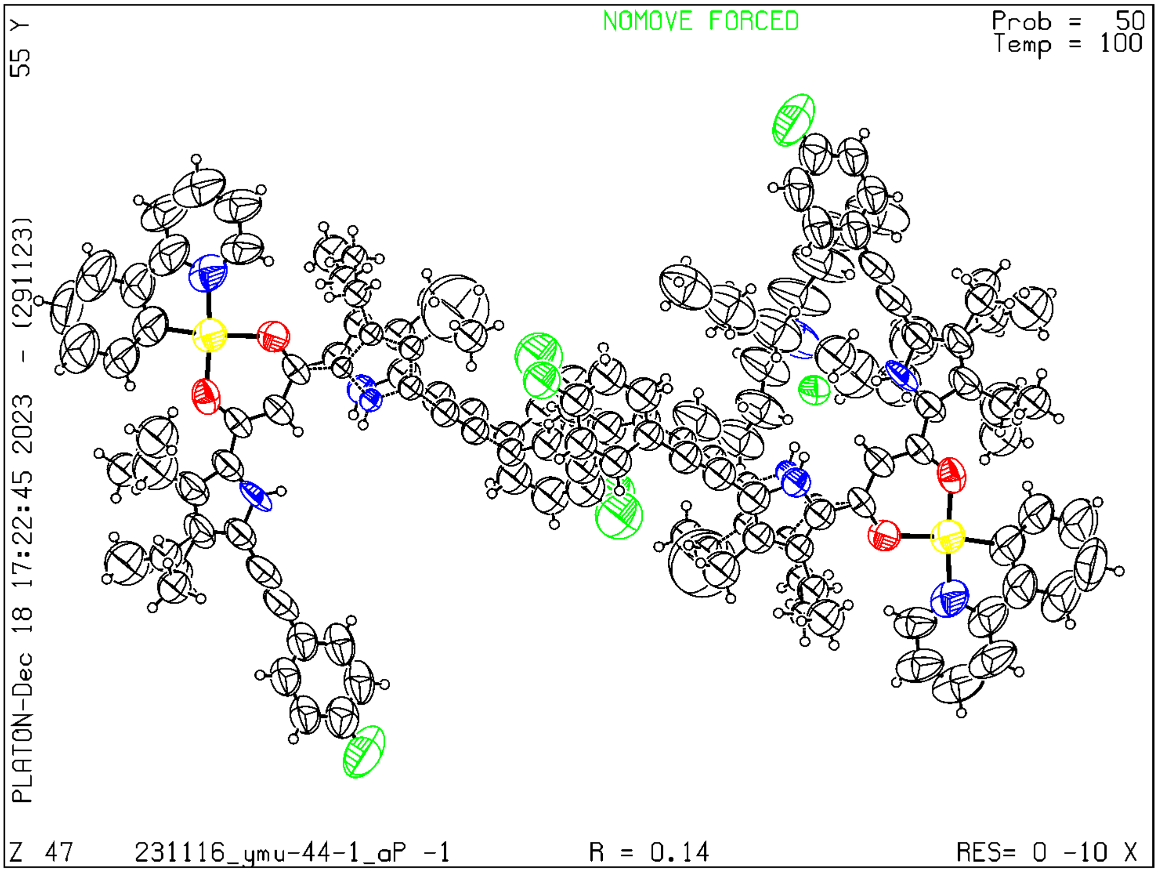

checkCIF/PLATON report

Structure factors have been supplied for datablock(s) ymu-16-4_a

THIS REPORT IS FOR GUIDANCE ONLY. IF USED AS PART OF A REVIEW PROCEDURE FOR PUBLICATION, IT SHOULD NOT REPLACE THE EXPERTISE OF AN EXPERIENCED CRYSTALLOGRAPHIC REFEREE.

No syntax errors found. CIF dictionary Interpreting this report

Datablock: ymu-16-4_a

Bond precision:	C-C = 0.0095 A	Wavelength=0.41340	
Cell:	a=21.253(11)	b=13.828(7)	c=26.237(13)
	alpha=90	beta=108.162(8)	gamma=90
Temperature:	90 K		

	Calculated	Reported
Volume	7327(6)	7326(6)
Space group	P 21/n	P 21/n
Hall group	-P 2yn	-P 2yn
Moiety formula	C46 H41 N3 O2 Pt, C44 H28 Au N4, Cl	C46 H41 N3 O2 Pt, C44 H28 Au N4, Cl
Sum formula	C90 H69 Au Cl N7 O2 Pt	C90 H69 Au Cl N7 O2 Pt
Mr	1708.02	1708.02
Dx,g cm-3	1.548	1.549
Z	4	4
Mu (mm-1)	0.989	0.989
F000	3392.0	3392.0
F000'	3387.72	
h,k,lmax	27,17,34	27,17,33
Nref	16787	16266
Tmin,Tmax	0.975,0.986	0.573,1.000
Tmin'	0.835	

Correction method= # Reported T Limits: Tmin=0.573 Tmax=1.000
AbsCorr = MULTI-SCAN

Data completeness= 0.969 Theta(max)= 15.569

R(reflections)= 0.0506(14576) wR2(reflections)= 0.1134(16266)

S = 1.149 Npar= 917

The following ALERTS were generated. Each ALERT has the format
test-name_ALERT_alert-type_alert-level.
Click on the hyperlinks for more details of the test.

Alert level C

```
PLAT220_ALERT_2_C NonSolvent   Resd 1  C   Ueq(max)/Ueq(min) Range        3.4 Ratio
PLAT222_ALERT_3_C NonSolvent Resd 1  H   Uiso(max)/Uiso(min) Range        4.1 Ratio
PLAT230_ALERT_2_C Hirshfeld Test Diff for    C13       --C14      .       6.1 s.u.
PLAT241_ALERT_2_C High   'MainMol' Ueq as Compared to Neighbors of        C14 Check
PLAT330_ALERT_2_C Large Aver Phenyl C-C Dist C59       --C64      .      1.41 Ang.
PLAT330_ALERT_2_C Large Aver Phenyl C-C Dist C70       --C75      .      1.41 Ang.
PLAT342_ALERT_3_C Low Bond Precision on  C-C Bonds ...............    0.00951 Ang.
PLAT369_ALERT_2_C Long   C(sp2)-C(sp2) Bond  C80       - C81      .      1.53 Ang.
PLAT906_ALERT_3_C Large K Value in the Analysis of Variance ......      7.735 Check
PLAT911_ALERT_3_C Missing FCF Refl Between Thmin & STh/L=    0.600          3 Report
PLAT971_ALERT_2_C Check Calcd Resid. Dens.  1.04Ang From Au1             1.87 eA-3
PLAT971_ALERT_2_C Check Calcd Resid. Dens.  0.95Ang From C14             1.74 eA-3
PLAT971_ALERT_2_C Check Calcd Resid. Dens.  1.08Ang From Au1             1.68 eA-3
PLAT972_ALERT_2_C Check Calcd Resid. Dens.  0.82Ang From Pt1            -1.94 eA-3
```

Alert level G

```
ABSMU01_ALERT_1_G  Calculation of _exptl_absorpt_correction_mu
                not performed for this radiation type.
PLAT002_ALERT_2_G Number of Distance or Angle Restraints on AtSite          3 Note
PLAT007_ALERT_5_G Number of Unrefined Donor-H Atoms ..............          2 Report
PLAT083_ALERT_2_G SHELXL Second Parameter in WGHT  Unusually Large      21.46 Why ?
PLAT092_ALERT_4_G Check: Wavelength Given is not Cu,Ga,Mo,Ag,In Ka    0.41340 Ang.
PLAT171_ALERT_4_G The CIF-Embedded .res File Contains EADP Records          1 Report
PLAT172_ALERT_4_G The CIF-Embedded .res File Contains DFIX Records          1 Report
PLAT371_ALERT_2_G Long   C(sp2)-C(sp1) Bond  C6        - C7       .      1.47 Ang.
PLAT371_ALERT_2_G Long   C(sp2)-C(sp1) Bond  C8        - C9       .      1.45 Ang.
PLAT371_ALERT_2_G Long   C(sp2)-C(sp1) Bond  C27       - C28      .      1.45 Ang.
PLAT371_ALERT_2_G Long   C(sp2)-C(sp1) Bond  C29       - C30      .      1.46 Ang.
PLAT794_ALERT_5_G Tentative Bond Valency for Au1        (III)     .      2.99 Info
PLAT794_ALERT_5_G Tentative Bond Valency for Pt1        (II)      .      1.96 Info
PLAT860_ALERT_3_G Number of Least-Squares Restraints .............          2 Note
PLAT883_ALERT_1_G No Info/Value for _atom_sites_solution_primary .     Please Do !
PLAT912_ALERT_4_G Missing # of FCF Reflections Above STh/L=  0.600        514 Note
PLAT933_ALERT_2_G Number of HKL-OMIT Records in Embedded .res File          4 Note
PLAT965_ALERT_2_G The SHELXL WEIGHT Optimisation has not Converged     Please Check
PLAT978_ALERT_2_G Number C-C Bonds with Positive Residual Density.          1 Info
```

0 **ALERT level A** = Most likely a serious problem - resolve or explain
0 **ALERT level B** = A potentially serious problem, consider carefully
14 **ALERT level C** = Check. Ensure it is not caused by an omission or oversight
19 **ALERT level G** = General information/check it is not something unexpected

2 ALERT type 1 CIF construction/syntax error, inconsistent or missing data
19 ALERT type 2 Indicator that the structure model may be wrong or deficient
5 ALERT type 3 Indicator that the structure quality may be low
4 ALERT type 4 Improvement, methodology, query or suggestion
3 ALERT type 5 Informative message, check

Validation response form

Please find below a validation response form (VRF) that can be filled in and pasted into your CIF.

```
# start Validation Reply Form
_vrf_PLAT220_ymu-16-4_a
;
PROBLEM: NonSolvent   Resd 1  C   Ueq(max)/Ueq(min) Range        3.4 Ratio
RESPONSE: ...
;
_vrf_PLAT222_ymu-16-4_a
;
PROBLEM: NonSolvent Resd 1  H   Uiso(max)/Uiso(min) Range        4.1 Ratio
RESPONSE: ...
;
_vrf_PLAT230_ymu-16-4_a
;
PROBLEM: Hirshfeld Test Diff for    C13      --C14      .        6.1 s.u.
RESPONSE: ...
;
_vrf_PLAT241_ymu-16-4_a
;
PROBLEM: High   'MainMol' Ueq as Compared to Neighbors of        C14 Check
RESPONSE: ...
;
_vrf_PLAT330_ymu-16-4_a
;
PROBLEM: Large Aver Phenyl C-C Dist C59      --C64      .       1.41 Ang.
RESPONSE: ...
;
_vrf_PLAT342_ymu-16-4_a
;
PROBLEM: Low Bond Precision on  C-C Bonds ..............    0.00951 Ang.
RESPONSE: ...
;
_vrf_PLAT369_ymu-16-4_a
;
PROBLEM: Long   C(sp2)-C(sp2) Bond  C80      - C81      .       1.53 Ang.
RESPONSE: ...
;
_vrf_PLAT906_ymu-16-4_a
;
PROBLEM: Large K Value in the Analysis of Variance ......      7.735 Check
RESPONSE: ...
;
_vrf_PLAT911_ymu-16-4_a
;
PROBLEM: Missing FCF Refl Between Thmin & STh/L=    0.600          3 Report
RESPONSE: ...
;
_vrf_PLAT971_ymu-16-4_a
;
PROBLEM: Check Calcd Resid. Dens.  1.04Ang From Au1             1.87 eA-3
RESPONSE: ...
;
_vrf_PLAT972_ymu-16-4_a
;
```

```
PROBLEM: Check Calcd Resid. Dens.  0.82Ang From Pt1           -1.94 eA-3
RESPONSE: ...
;
# end Validation Reply Form
```

It is advisable to attempt to resolve as many as possible of the alerts in all categories. Often the minor alerts point to easily fixed oversights, errors and omissions in your CIF or refinement strategy, so attention to these fine details can be worthwhile. In order to resolve some of the more serious problems it may be necessary to carry out additional measurements or structure refinements. However, the purpose of your study may justify the reported deviations and the more serious of these should normally be commented upon in the discussion or experimental section of a paper or in the "special_details" fields of the CIF. checkCIF was carefully designed to identify outliers and unusual parameters, but every test has its limitations and alerts that are not important in a particular case may appear. Conversely, the absence of alerts does not guarantee there are no aspects of the results needing attention. It is up to the individual to critically assess their own results and, if necessary, seek expert advice.

Publication of your CIF in IUCr journals

A basic structural check has been run on your CIF. These basic checks will be run on all CIFs submitted for publication in IUCr journals (*Acta Crystallographica*, *Journal of Applied Crystallography*, *Journal of Synchrotron Radiation*); however, if you intend to submit to *Acta Crystallographica Section C* or *E* or *IUCrData*, you should make sure that full publication checks are run on the final version of your CIF prior to submission.

Publication of your CIF in other journals

Please refer to the *Notes for Authors* of the relevant journal for any special instructions relating to CIF submission.

PLATON version of 19/02/2022; check.def file version of 19/02/2022

Datablock ymu-16-4_a - ellipsoid plot

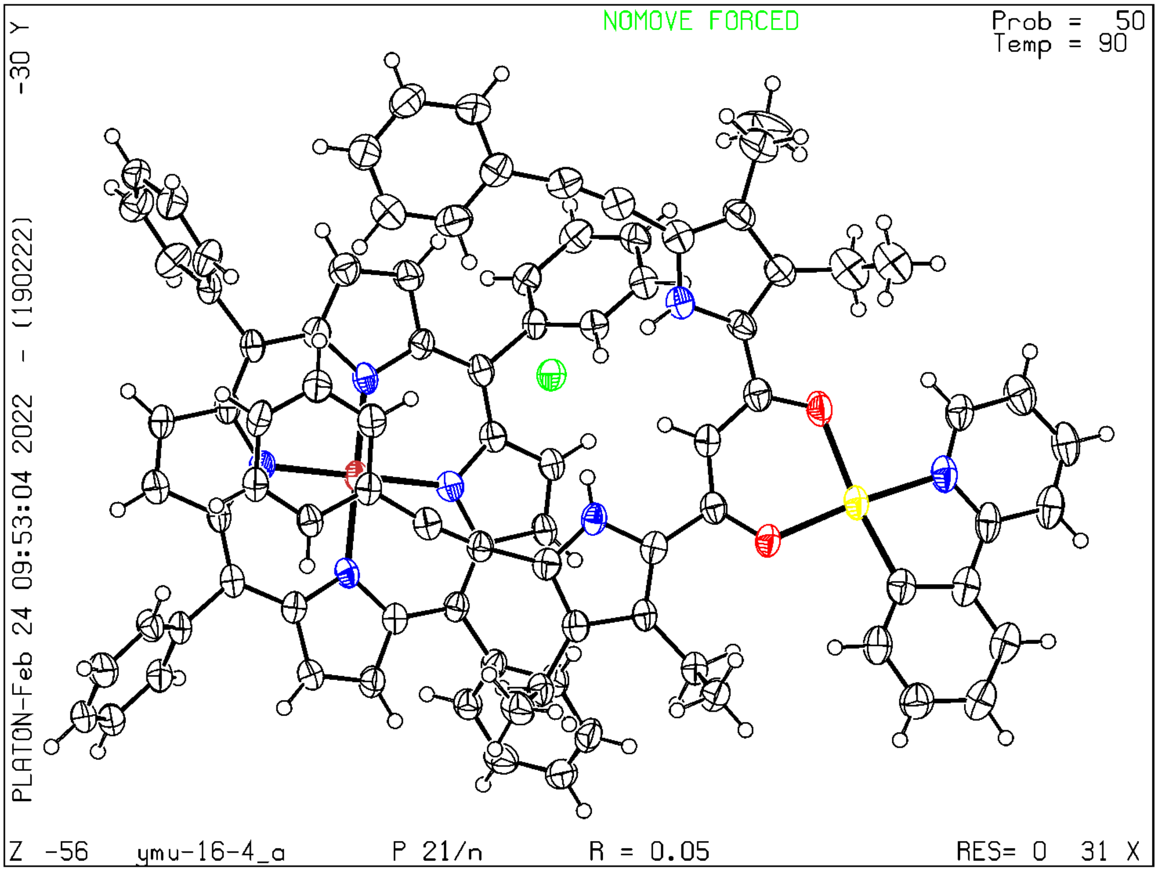

checkCIF/PLATON report

Structure factors have been supplied for datablock(s) ymu-37-1

THIS REPORT IS FOR GUIDANCE ONLY. IF USED AS PART OF A REVIEW PROCEDURE FOR PUBLICATION, IT SHOULD NOT REPLACE THE EXPERTISE OF AN EXPERIENCED CRYSTALLOGRAPHIC REFEREE.

No syntax errors found. CIF dictionary Interpreting this report

Datablock: ymu-37-1

Bond precision: C-C = 0.0096 A Wavelength=0.81070

Cell:	a=12.9662(9)	b=13.3402(9)	c=22.4086(18)
	alpha=105.8934(15)	beta=105.4630(15)	gamma=94.294(2)
Temperature:	90 K		

	Calculated	Reported
Volume	3546.9(4)	3546.9(4)
Space group	P -1	P -1
Hall group	-P 1	-P 1
Moiety formula	2(C46 H39 F2 N3 O2 Pt), 2(C44 H28 Au N4), C3 H6 O, 2(Cl)	C46 H39 F2 N3 O2 Pt, C44 H28 Au N4, Cl, 0.5(C3 H6 O)
Sum formula	C183 H140 Au2 Cl2 F4 N14 O5 Pt2	C91.50 H70 Au Cl F2 N7 O2.50 Pt
Mr	3546.09	1773.05
Dx,g cm-3	1.660	1.660
Z	1	2
Mu (mm-1)	5.744	5.744
F000	1760.0	1760.0
F000'	1746.83	
h,k,lmax	15,16,26	15,16,26
Nref	13002	12978
Tmin,Tmax	0.223,0.750	
Tmin'	0.143	

Correction method= Not given

Data completeness= 0.998 Theta(max)= 29.233

R(reflections)= 0.0483(11824)   wR2(reflections)= 0.1589(12978)

S = 1.205   Npar= 958

The following ALERTS were generated. Each ALERT has the format
test-name_ALERT_alert-type_alert-level.
Click on the hyperlinks for more details of the test.

Alert level A

```
PLAT059_ALERT_1_A Minimum Transmission Factor Missing ...........           ?
PLAT974_ALERT_2_A Check Calcd Negative Resid. Density on       Pt1      -3.80 eA-3
```

Author Response: The large peaks around Au and Pt atoms are the ghost peak derived from the ripple of fourier series truncation error.

```
PLAT974_ALERT_2_A Check Calcd Negative Resid. Density on       Au1      -3.61 eA-3
```

Author Response: The large peaks around Au and Pt atoms are the ghost peak derived from the ripple of fourier series truncation error.

Alert level B

```
PLAT934_ALERT_3_B Number of (Iobs-Icalc)/Sigma(W) > 10 Outliers ..          4 Check
PLAT972_ALERT_2_B Check Calcd Resid. Dens.  0.73Ang From Au1            -2.88 eA-3
PLAT972_ALERT_2_B Check Calcd Resid. Dens.  0.62Ang From Pt1            -2.87 eA-3
```

Alert level C

```
PLAT042_ALERT_1_C Calc. and Reported MoietyFormula Strings  Differ     Please Check
PLAT085_ALERT_2_C SHELXL Default Weighting Scheme is not Optimized     Please Check
PLAT220_ALERT_2_C NonSolvent   Resd 1   C   Ueq(max)/Ueq(min) Range        3.7 Ratio
PLAT221_ALERT_2_C Solv./Anion  Resd 3   C   Ueq(max)/Ueq(min) Range        4.6 Ratio
PLAT222_ALERT_3_C NonSolvent Resd 1   H   Uiso(max)/Uiso(min) Range        4.2 Ratio
PLAT234_ALERT_4_C Large Hirshfeld Difference C1       --C2       .        0.18 Ang.
PLAT250_ALERT_2_C Large U3/U1 Ratio for Average U(i,j) Tensor ....         2.9 Note
PLAT334_ALERT_2_C Small <C-C> Benzene Dist.   C1       -C6       .        1.37 Ang.
PLAT342_ALERT_3_C Low Bond Precision on  C-C Bonds ..............      0.00956 Ang.
PLAT414_ALERT_2_C Short Intra D-H..H-X       H1        ..H18      .        1.92 Ang.
                                                      x,y,z  =      1_555 Check
PLAT414_ALERT_2_C Short Intra D-H..H-X       H2A       ..H18      .        1.98 Ang.
                                                      x,y,z  =      1_555 Check
PLAT732_ALERT_1_C Angle   Calc   179.86(7), Rep    179.8(2) ......        2.86 s.u.-R
              N5      -AU1     -N7         1_555  1_555  1_555     #  182 Check
PLAT906_ALERT_3_C Large K Value in the Analysis of Variance ......       2.135 Check
PLAT911_ALERT_3_C Missing FCF Refl Between Thmin & STh/L=    0.600          21 Report
PLAT971_ALERT_2_C Check Calcd Resid. Dens.  0.34Ang From C92             2.50 eA-3
PLAT971_ALERT_2_C Check Calcd Resid. Dens.  0.98Ang From Pt1             1.84 eA-3
PLAT971_ALERT_2_C Check Calcd Resid. Dens.  0.98Ang From Au1             1.67 eA-3
PLAT971_ALERT_2_C Check Calcd Resid. Dens.  1.12Ang From Pt1             1.51 eA-3
PLAT972_ALERT_2_C Check Calcd Resid. Dens.  0.65Ang From Pt1            -2.49 eA-3
```

```
PLAT972_ALERT_2_C Check Calcd Resid. Dens.  2.17Ang From C91          -1.83 eA-3
PLAT972_ALERT_2_C Check Calcd Resid. Dens.  1.32Ang From C31          -1.75 eA-3
PLAT977_ALERT_2_C Check Negative Difference Density on H31      .      -0.44 eA-3
```

Alert level G

```
ABSMU01_ALERT_1_G  Calculation of _exptl_absorpt_correction_mu
                not performed for this radiation type.
PLAT002_ALERT_2_G Number of Distance or Angle Restraints on AtSite          2 Note
PLAT007_ALERT_5_G Number of Unrefined Donor-H Atoms .............           2 Report
PLAT045_ALERT_1_G Calculated and Reported Z Differ by a Factor ...      0.500 Check
PLAT092_ALERT_4_G Check: Wavelength Given is not Cu,Ga,Mo,Ag,In Ka    0.81070 Ang.
PLAT171_ALERT_4_G The CIF-Embedded .res File Contains EADP Records          1 Report
PLAT172_ALERT_4_G The CIF-Embedded .res File Contains DFIX Records          1 Report
PLAT174_ALERT_4_G The CIF-Embedded .res File Contains FLAT Records          1 Report
PLAT300_ALERT_4_G Atom Site Occupancy of O3         Constrained at        0.5 Check
PLAT300_ALERT_4_G Atom Site Occupancy of C91        Constrained at        0.5 Check
PLAT300_ALERT_4_G Atom Site Occupancy of C92        Constrained at        0.5 Check
PLAT300_ALERT_4_G Atom Site Occupancy of C93        Constrained at        0.5 Check
PLAT300_ALERT_4_G Atom Site Occupancy of H90        Constrained at        0.5 Check
PLAT300_ALERT_4_G Atom Site Occupancy of H91        Constrained at        0.5 Check
PLAT300_ALERT_4_G Atom Site Occupancy of H92        Constrained at        0.5 Check
PLAT300_ALERT_4_G Atom Site Occupancy of H93        Constrained at        0.5 Check
PLAT300_ALERT_4_G Atom Site Occupancy of H94        Constrained at        0.5 Check
PLAT300_ALERT_4_G Atom Site Occupancy of H95        Constrained at        0.5 Check
PLAT302_ALERT_4_G Anion/Solvent/Minor-Residue Disorder (Resd  3  )       100% Note
PLAT371_ALERT_2_G Long   C(sp2)-C(sp1) Bond  C4       - C7       .       1.41 Ang.
PLAT371_ALERT_2_G Long   C(sp2)-C(sp1) Bond  C8       - C9       .       1.41 Ang.
PLAT371_ALERT_2_G Long   C(sp2)-C(sp1) Bond  C29      - C30      .       1.43 Ang.
PLAT432_ALERT_2_G Short Inter X...Y Contact  C4       ..C42      .       3.19 Ang.
                                                    x,1+y,z  =      1_565 Check
PLAT432_ALERT_2_G Short Inter X...Y Contact  C43      ..C77      .       3.16 Ang.
                                                   -1+x,y,z  =      1_455 Check
PLAT789_ALERT_4_G Atoms with Negative _atom_site_disorder_group  #         10 Check
PLAT794_ALERT_5_G Tentative Bond Valency for Au1       (III)     .       3.43 Info
PLAT794_ALERT_5_G Tentative Bond Valency for Pt1       (II)      .       2.21 Info
PLAT822_ALERT_4_G CIF-embedded .res Contains Negative PART Numbers          1 Check
PLAT860_ALERT_3_G Number of Least-Squares Restraints ............           2 Note
PLAT883_ALERT_1_G No Info/Value for _atom_sites_solution_primary .     Please Do !
PLAT912_ALERT_4_G Missing # of FCF Reflections Above STh/L=  0.600          3 Note
PLAT933_ALERT_2_G Number of HKL-OMIT Records in Embedded .res File          8 Note
PLAT941_ALERT_3_G Average HKL Measurement Multiplicity ..........         3.0 Low
PLAT965_ALERT_2_G The SHELXL WEIGHT Optimisation has not Converged     Please Check
PLAT978_ALERT_2_G Number C-C Bonds with Positive Residual Density.          0 Info
```

---

3 **ALERT level A** = Most likely a serious problem - resolve or explain
3 **ALERT level B** = A potentially serious problem, consider carefully
22 **ALERT level C** = Check. Ensure it is not caused by an omission or oversight
35 **ALERT level G** = General information/check it is not something unexpected

6 ALERT type 1 CIF construction/syntax error, inconsistent or missing data
28 ALERT type 2 Indicator that the structure model may be wrong or deficient
7 ALERT type 3 Indicator that the structure quality may be low
19 ALERT type 4 Improvement, methodology, query or suggestion
3 ALERT type 5 Informative message, check

It is advisable to attempt to resolve as many as possible of the alerts in all categories. Often the minor alerts point to easily fixed oversights, errors and omissions in your CIF or refinement strategy, so attention to these fine details can be worthwhile. In order to resolve some of the more serious problems it may be necessary to carry out additional measurements or structure refinements. However, the purpose of your study may justify the reported deviations and the more serious of these should normally be commented upon in the discussion or experimental section of a paper or in the "special_details" fields of the CIF. checkCIF was carefully designed to identify outliers and unusual parameters, but every test has its limitations and alerts that are not important in a particular case may appear. Conversely, the absence of alerts does not guarantee there are no aspects of the results needing attention. It is up to the individual to critically assess their own results and, if necessary, seek expert advice.

Publication of your CIF in IUCr journals

A basic structural check has been run on your CIF. These basic checks will be run on all CIFs submitted for publication in IUCr journals (*Acta Crystallographica*, *Journal of Applied Crystallography*, *Journal of Synchrotron Radiation*); however, if you intend to submit to *Acta Crystallographica Section C* or *E* or *IUCrData*, you should make sure that full publication checks are run on the final version of your CIF prior to submission.

Publication of your CIF in other journals

Please refer to the *Notes for Authors* of the relevant journal for any special instructions relating to CIF submission.

Validation response form

Please find below a validation response form (VRF) that can be filled in and pasted into your CIF.

```
# start Validation Reply Form
_vrf_PLAT059_ymu-37-1
;
PROBLEM: Minimum Transmission Factor Missing ...........          ?
RESPONSE: ...
;
_vrf_PLAT934_ymu-37-1
;
PROBLEM: Number of (Iobs-Icalc)/Sigma(W) > 10 Outliers ..          4 Check
RESPONSE: ...
;
_vrf_PLAT972_ymu-37-1
;
PROBLEM: Check Calcd Resid. Dens.  0.73Ang From Au1            -2.88 eA-3
RESPONSE: ...
;
_vrf_PLAT042_ymu-37-1
;
PROBLEM: Calc. and Reported MoietyFormula Strings  Differ     Please Check
RESPONSE: ...
;
_vrf_PLAT085_ymu-37-1
```

```
;
PROBLEM: SHELXL Default Weighting Scheme is not Optimized     Please Check
RESPONSE: ...
;
_vrf_PLAT220_ymu-37-1
;
PROBLEM: NonSolvent   Resd 1  C   Ueq(max)/Ueq(min) Range        3.7 Ratio
RESPONSE: ...
;
_vrf_PLAT221_ymu-37-1
;
PROBLEM: Solv./Anion  Resd 3  C   Ueq(max)/Ueq(min) Range        4.6 Ratio
RESPONSE: ...
;
_vrf_PLAT222_ymu-37-1
;
PROBLEM: NonSolvent Resd 1  H   Uiso(max)/Uiso(min) Range        4.2 Ratio
RESPONSE: ...
;
_vrf_PLAT234_ymu-37-1
;
PROBLEM: Large Hirshfeld Difference C1       --C2       .       0.18 Ang.
RESPONSE: ...
;
_vrf_PLAT250_ymu-37-1
;
PROBLEM: Large U3/U1 Ratio for Average U(i,j) Tensor ....        2.9 Note
RESPONSE: ...
;
_vrf_PLAT334_ymu-37-1
;
PROBLEM: Small <C-C> Benzene Dist.   C1       -C6       .       1.37 Ang.
RESPONSE: ...
;
_vrf_PLAT342_ymu-37-1
;
PROBLEM: Low Bond Precision on  C-C Bonds ..............    0.00956 Ang.
RESPONSE: ...
;
_vrf_PLAT414_ymu-37-1
;
PROBLEM: Short Intra D-H..H-X       H1       ..H18      .       1.92 Ang.
RESPONSE: ...
;
_vrf_PLAT732_ymu-37-1
;
PROBLEM: Angle   Calc   179.86(7), Rep    179.8(2) ......       2.86 s.u.-R
RESPONSE: ...
;
_vrf_PLAT906_ymu-37-1
;
PROBLEM: Large K Value in the Analysis of Variance ......      2.135 Check
RESPONSE: ...
;
_vrf_PLAT911_ymu-37-1
;
PROBLEM: Missing FCF Refl Between Thmin & STh/L=    0.600         21 Report
```

```
RESPONSE: ...
;
_vrf_PLAT971_ymu-37-1
;
PROBLEM: Check Calcd Resid. Dens.  0.34Ang From C92            2.50 eA-3
RESPONSE: ...
;
_vrf_PLAT977_ymu-37-1
;
PROBLEM: Check Negative Difference Density on H31      .      -0.44 eA-3
RESPONSE: ...
;
# end Validation Reply Form
```

PLATON version of 06/07/2023; check.def file version of 30/06/2023

Datablock ymu-37-1 - ellipsoid plot

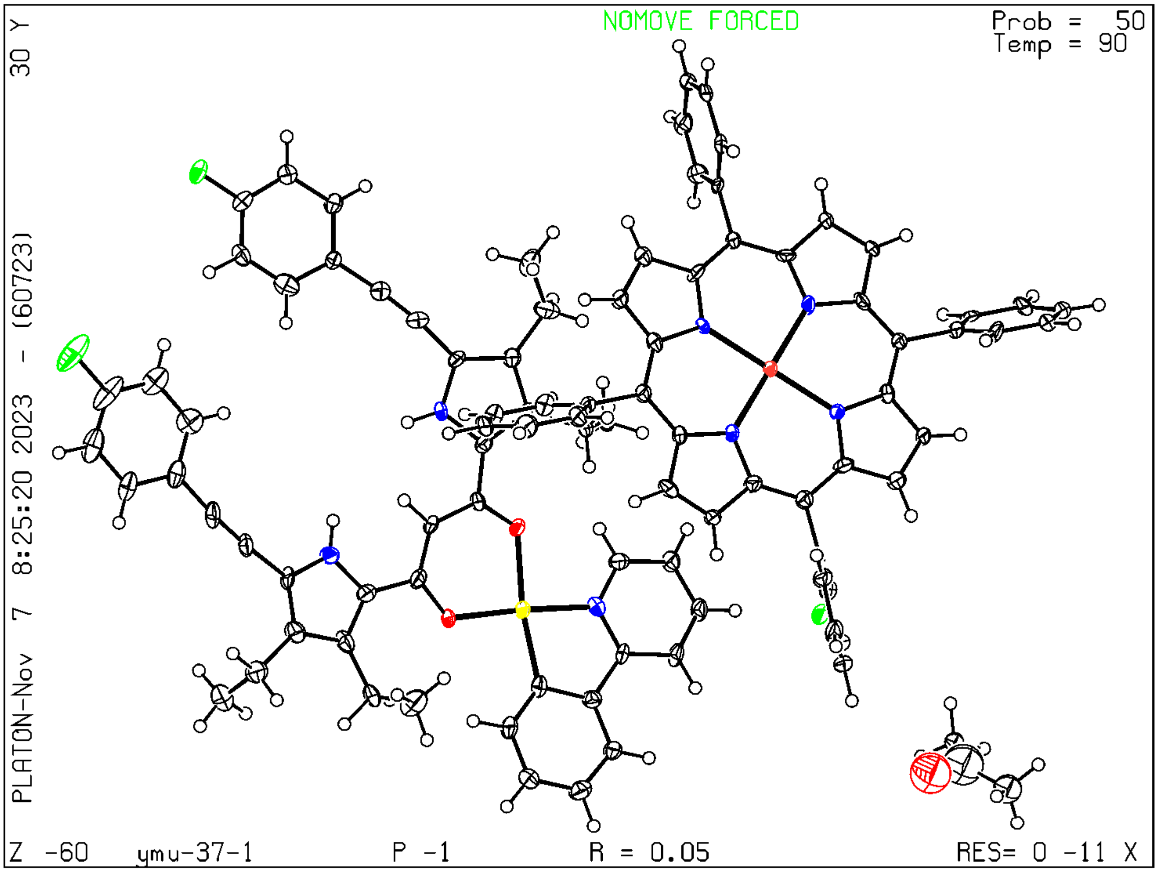

Supplement: Supplemental Material [file TSTA_A_2313958_SM6117.pdf]
