# Supplementary material for: Ion-pairing assemblies of π-extended anion-responsive organoplatinum complexes
Source: Sci Technol Adv Mater. 2024 Feb 6;25(1):2313958. doi: 10.1080/14686996.2024.2313958 (PMC10898271; doi:10.1080/14686996.2024.2313958)
Supplement: Supplemental Material [file TSTA_A_2313958_SM6114.pdf]

Supporting Information

Ion-pairing assemblies of π-extended anion-responsive organoplatinum complexes

Yohei Haketa, Yu Murakami, and Hiromitsu Maeda*

Department of Applied Chemistry, College of Life Sciences, Ritsumeikan University, Kusatsu 525–8577, Japan, E-mail: maedahir@ph.ritsumei.ac.jp

Table of Contents

1. Spectroscopic data S2
Figure S1–4 ^{1}H and ^{13}C NMR spectra. S2
Figure S5 UV/vis absorption spectra. S5
2. X-ray crystallographic data S6
Figure S6–10 Ortep drawings. S6
Figure S11–14 Packing diagrams. S9
Figure S15–18 Hirshfeld surfaces. S13
3. Theoretical studies S17
Figure S19,**20** Optimized structures. S19
Figure S21–24 Molecular orbitals and theoretical UV/vis absorption spectra. S21
Figure S25 Electrostatic potential (ESP) mapping. S22
Figure S26–29 EDA calculations. S22
Cartesian coordination of optimized structures S24
4. Anion-binding behaviors S37
Figure S30 UV/vis absorption spectral changes and corresponding titration plots. S37
Figure S31 ^{1}H NMR spectral changes. S38
5. Solution-state excited-state properties S39
Figure S32,33 Emission spectra. S39
Figure S34 Emission decay profiles. S39

1. Spectroscopic data

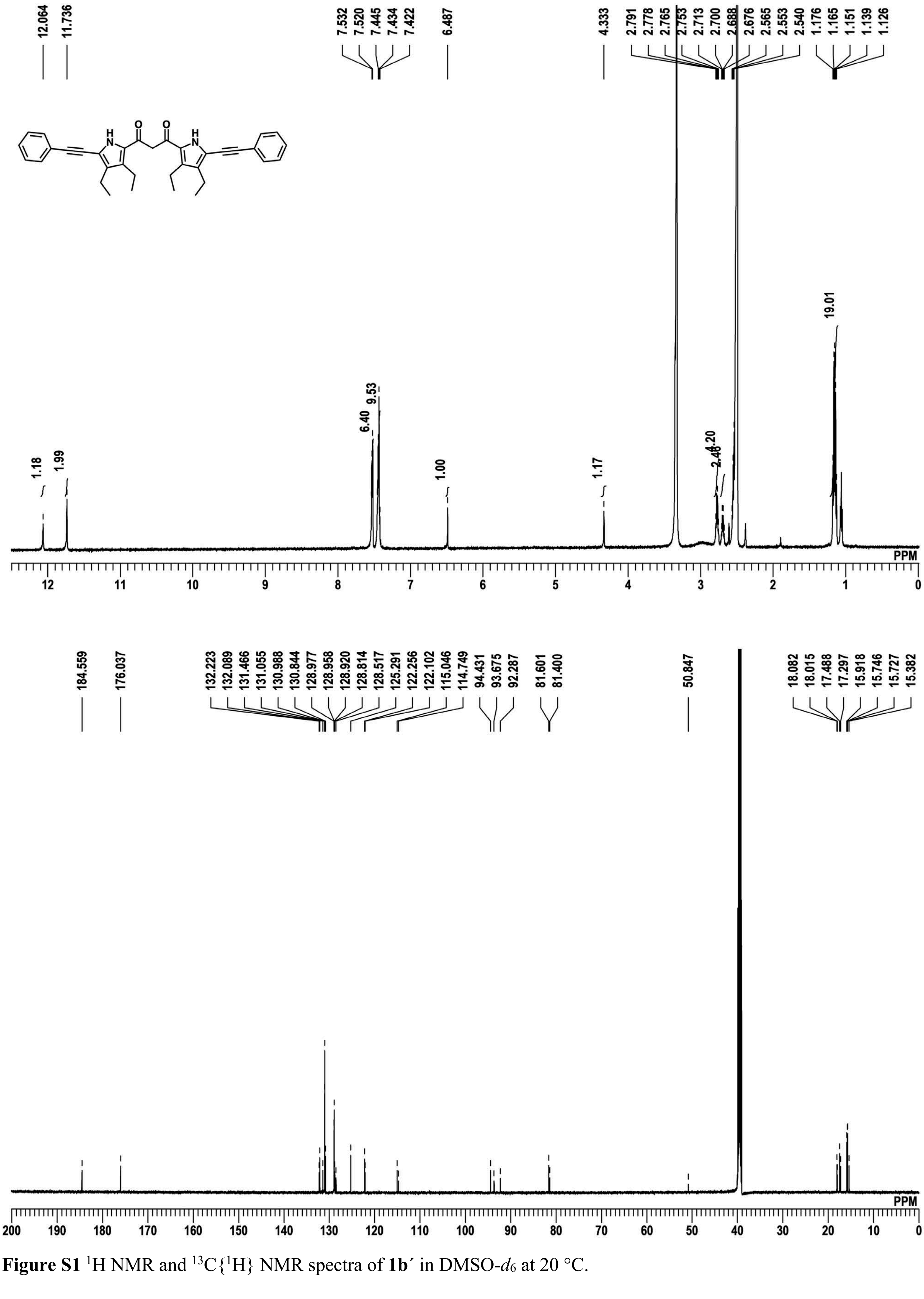

Figure S1 ^{1}H NMR and ^{13}C{^{1}H} NMR spectra of **1b´** in DMSO-d_6 at 20 °C.

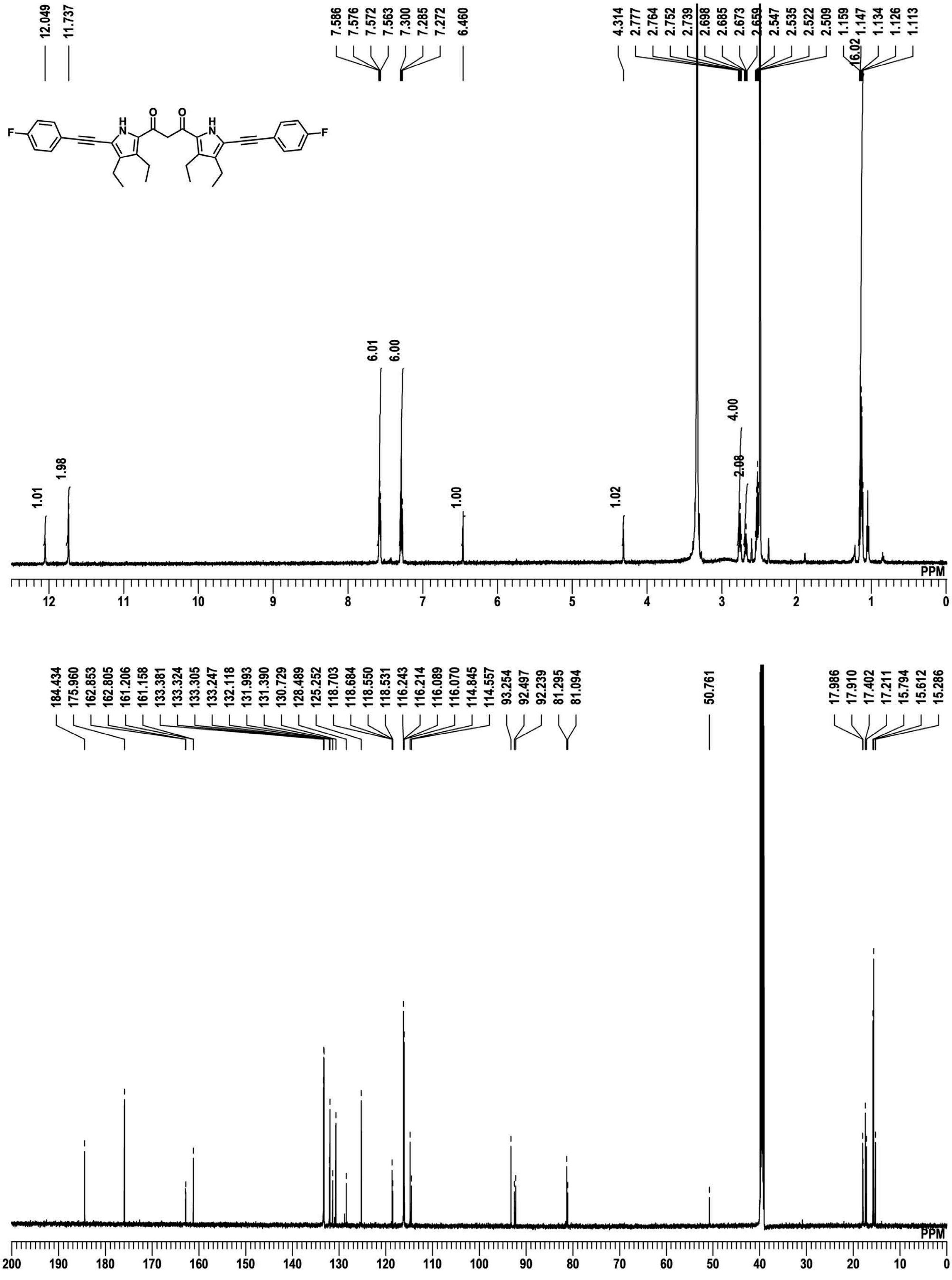

Figure S2 ^{1}H NMR and ^{13}C{^{1}H} NMR spectra of **1c´** in DMSO-d_6 at 20 °C.

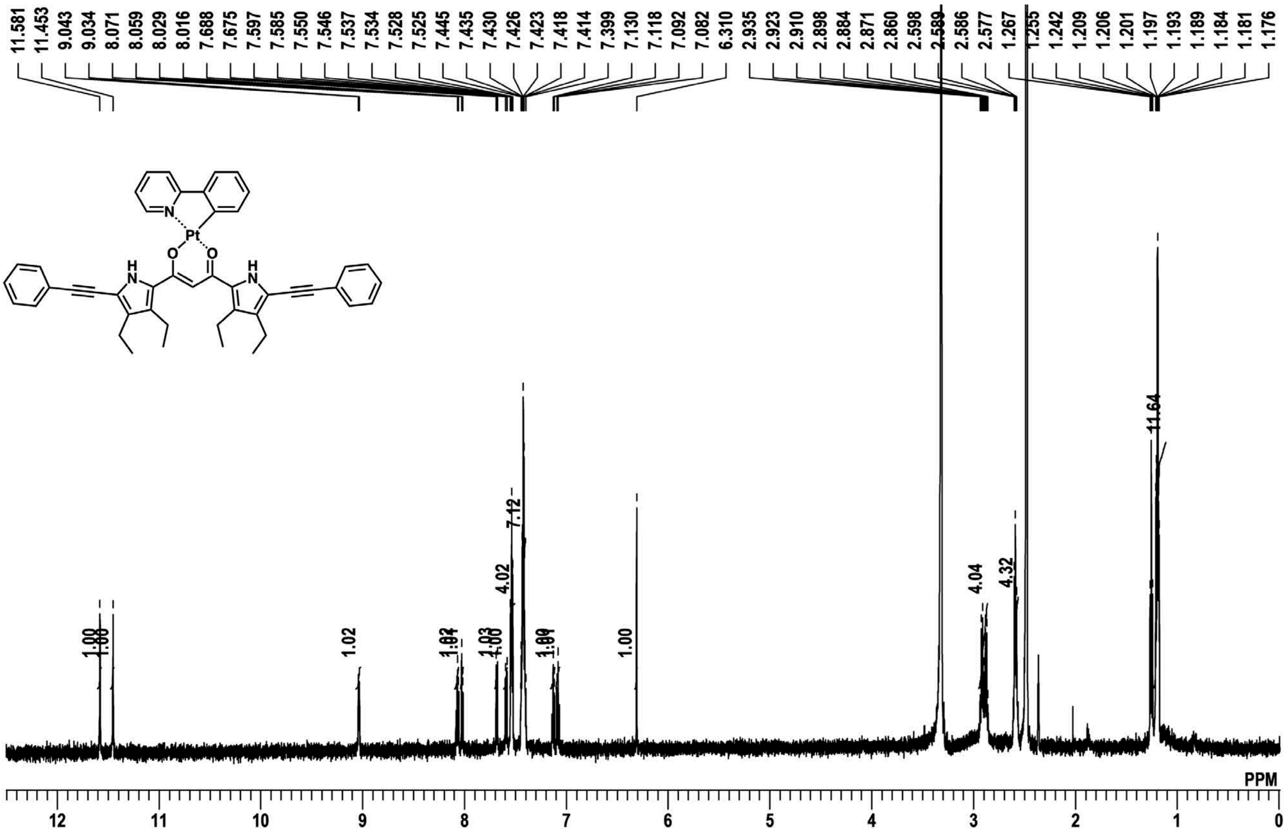

Figure S3 ^{1}H NMR spectrum of **2b** in DMSO-d_6 at 20 °C. $^{13}C\{^{1}H\}$ NMR spectrum was not measured due to the decomposition after prolonged measurement time.

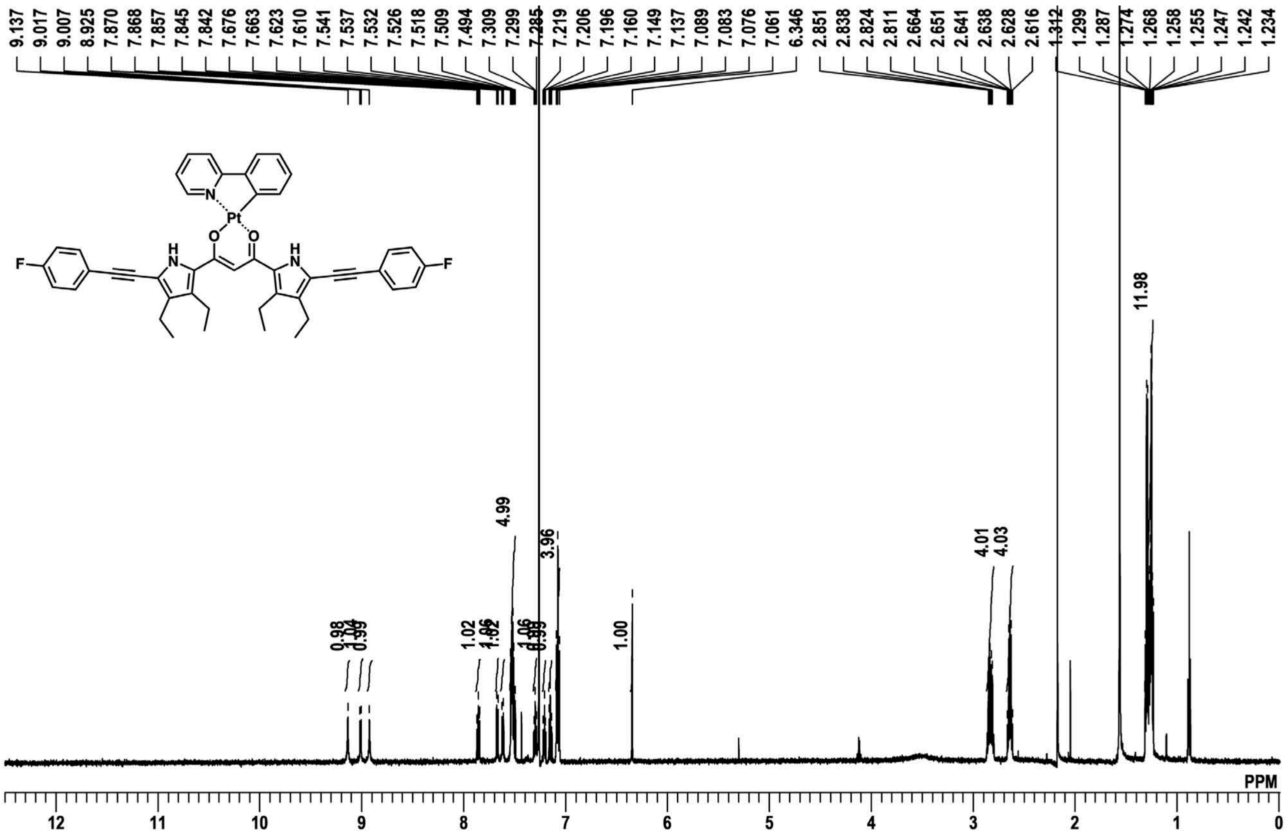

Figure S4 ^{1}H NMR spectrum of **2c** in $CDCl_3$ at 20 °C. $^{13}C\{^{1}H\}$ NMR spectrum was not measured due to the decomposition after prolonged measurement time.

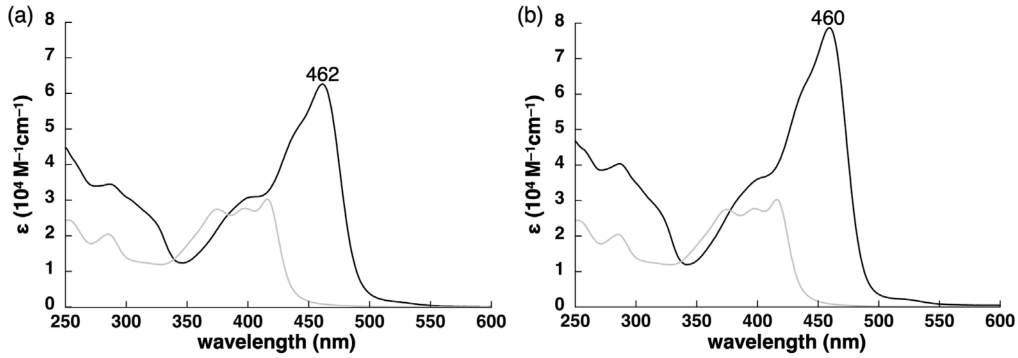

Figure S5 UV/vis absorption spectra of (a) **2b** and (b) **2c** in CH_2Cl_2 (0.02 mM) along with unsubstituted Pt^{II} complex **2a** as a reference (gray).

2. X-ray crystallographic data

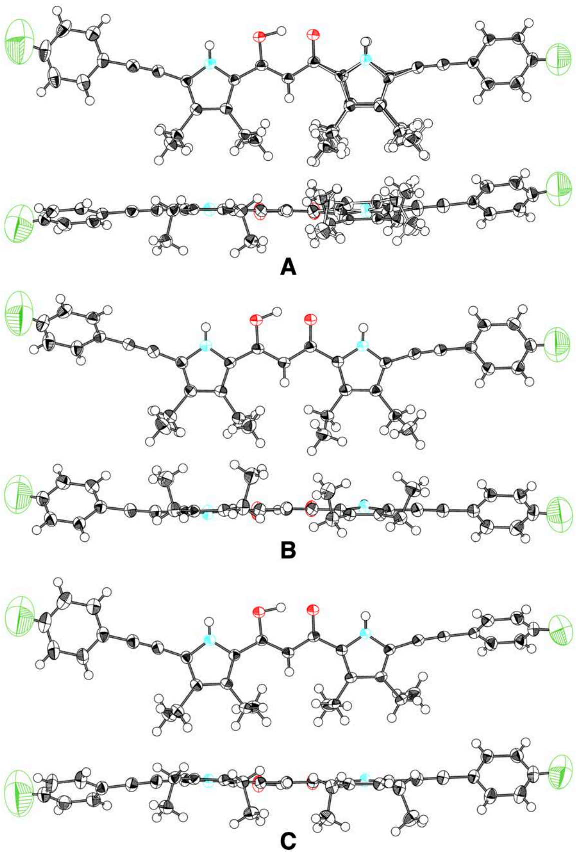

Figure S6 Ortep drawings of single-crystal X-ray structure (top and side views) of **1c**´ as three independent structures A–C. Thermal ellipsoids are scaled to the 50% probability level. In structure A, disordered structures are represented by black and white bonds for major and minor structures, respectively, in the ratio of 58.2:41.8 for a pyrrole unit. Atom color code: black, blue, red, and green refer to carbon, nitrogen, oxygen, and fluorine, respectively.

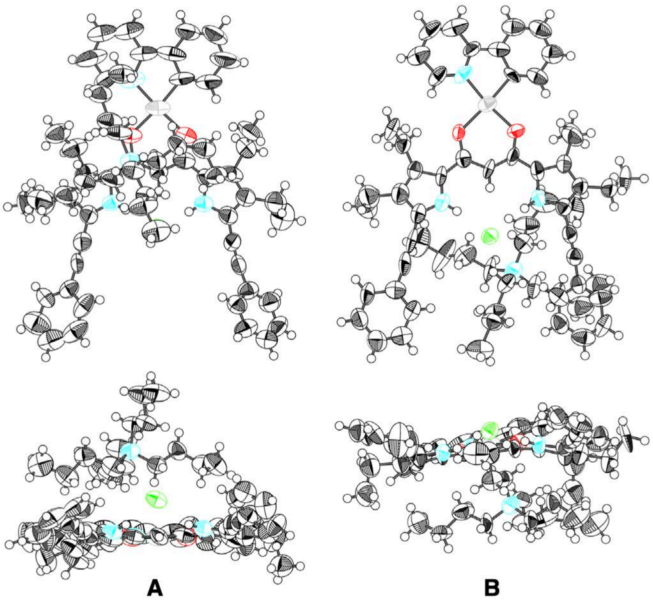

Figure S7 Ortep drawings of single-crystal X-ray structure (top and side views) of **2b**·Cl^{-}-TBA^{+} as two independent structures (A,B). Thermal ellipsoids are scaled to the 50% probability level. Atom color code: black, blue, red, yellow green, and gray refer to carbon, nitrogen, oxygen, chlorine, and platinum, respectively.

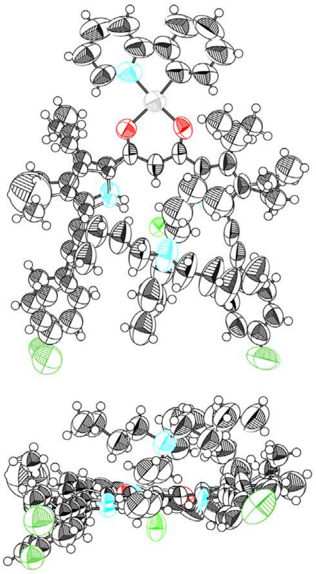

Figure S8 Ortep drawings of single-crystal X-ray structure (top and side views) of **2c**·Cl^{-}-TBA^{+}. Thermal ellipsoids are scaled to the 50% probability level. Disordered structures are represented by black and white bonds for major and minor structures, respectively, in the ratio of 55.6:44.4 for one side of the pyrrole unit with arylethynyl and 60.0:40.0 for the ethyl units. Atom color code: black, blue, red, green, yellow green, and gray refer to carbon, nitrogen, oxygen, fluorine, chlorine, and platinum, respectively.

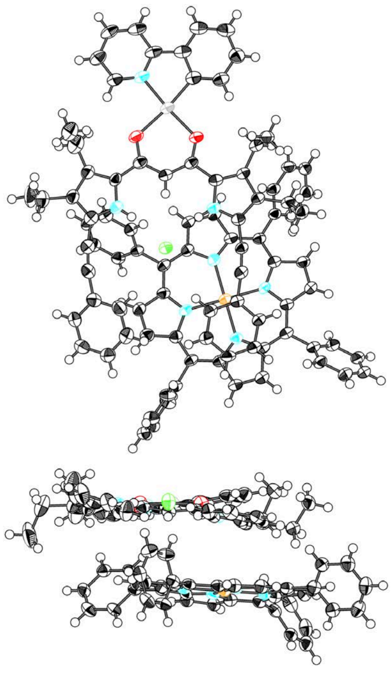

Figure S9 Ortep drawings of single-crystal X-ray structure (top and side views) of **2b**·Cl^{-}-$TPPAu^{+}$. Thermal ellipsoids are scaled to the 50% probability level. Atom color code: black, blue, red, yellow green, gray, and orange refer to carbon, nitrogen, oxygen, chlorine, platinum, and gold, respectively.

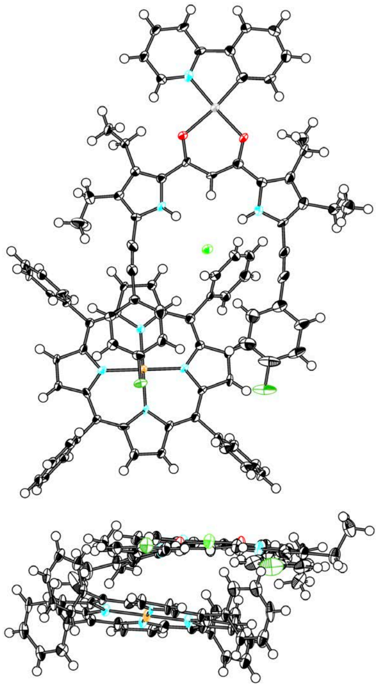

Figure S10 Ortep drawings of single-crystal X-ray structure (top and side views) of **2c**·Cl⁻-TPPAu⁺. Thermal ellipsoids are scaled to the 50% probability level. Atom color code: black, blue, red, green, yellow green, gray, and orange refer to carbon, nitrogen, oxygen, fluorine, chlorine, platinum, and gold, respectively. Solvent molecules are omitted for clarity.

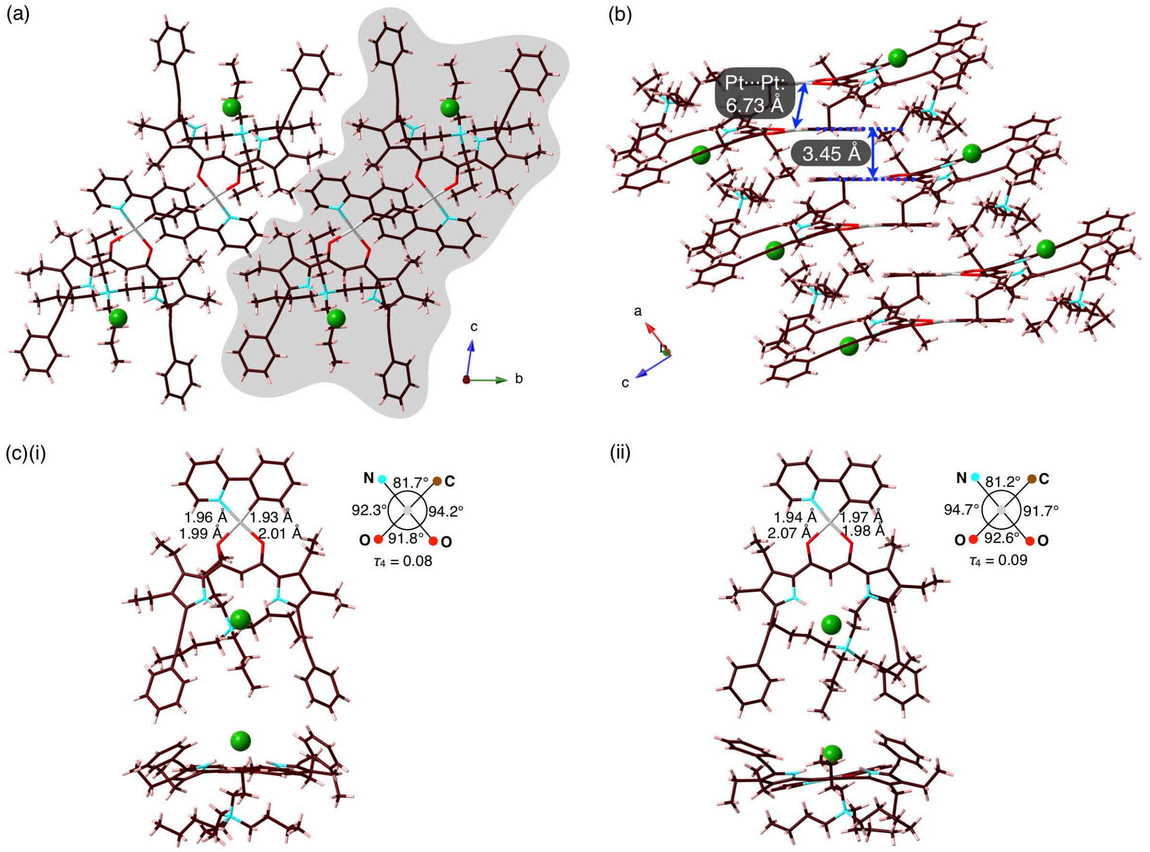

Figure S11 Packing diagrams of **2b**·Cl$^-$-TBA$^+$, which forms a charge-by-charge assembly: (a) the packing structure viewed from the top view, (b) packing structure as charge-by-charge assembly, and (c) two independent structures of ion pair **2b**·Cl$^-$-TBA$^+$. The stacking distances between two phenylpyridine mean planes (12 atoms of phenylpyridine) is 3.45 Å. The closest Pt···Pt distance is 6.73 Å, indicating the absence of Pt···Pt interaction. **2b**·Cl$^-$ in (c)(i) exhibits [1+1]-type anion complex through hydrogen-bonding interactions with N(–H)···Cl$^-$, $C_{bridging}$(–H)···Cl$^-$, and C_{phenyl}(–H)···Cl distances of 3.18/3.19, 3.60, and 5.46/4.66 Å, respectively. The other independent structure in (c)(ii) exhibits hydrogen-bonding interactions with N(–H)···Cl$^-$, $C_{bridging}$(–H)···Cl$^-$, and C_{phenyl}(–H)···Cl distances of 3.12/3.12, 3.50, and 5.45/5.70 Å, respectively. Mean-plane deviations of the receptor units for two independent structures (core 33 atoms including Pt and Cl$^-$) are 0.37 and 0.49 Å. The N···Pt, C···Pt, and O···Pt distances of **2b**·Cl$^-$ for the two independent structures are 1.96, 1.93, and 1.99/2.01 Å, respectively, and 1.94, 1.97, and 2.07/1.98 Å, respectively. The angles around Pt^{II} for the two independent structures are 81.7°, 94.2°, 91.8°, and 92.3° with τ_4 value of 0.08, and 81.2°, 91.7°, 92.6°, and 94.7° with τ_4 value of 0.09.[S1] Atom color code: brown, pink, blue, red, yellow green (spherical), and gray refer to carbon, hydrogen, nitrogen, oxygen, chlorine, and platinum, respectively.

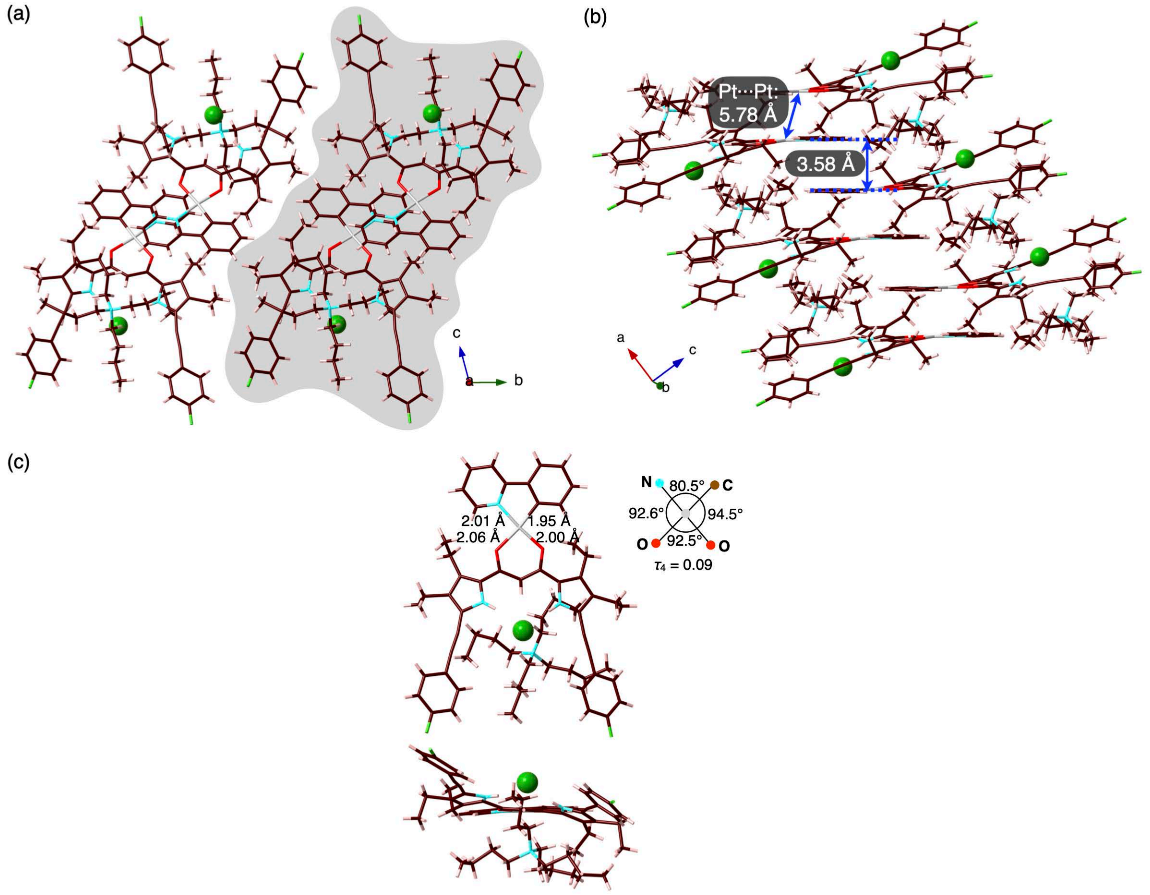

Figure S12 Packing diagrams of **2c**·Cl^{-}-TBA^{+}, which forms a charge-by-charge assembly: (a) the packing structure viewed from the top view, (b) packing structure as charge-by-charge assembly, and (c) ion pair **2c**·Cl^{-}-TBA^{+}. The stacking distance between two phenylpyridine mean planes (12-atoms of phenylpyridine) is 3.58 Å. The closest Pt···Pt distance is 5.78 Å, indicating the absence of Pt···Pt interaction. **2c**·Cl^{-} exhibits [1+1]-type anion complex through hydrogen-bonding interactions with N(–H)···Cl^{-}, $C_{bridging}$(–H)···Cl^{-}, and C_{phenyl}(–H)···Cl distances of 3.28/3.17, 3.60, and 5.54/5.26 Å, respectively. Mean-plane deviation of the receptor unit (core 33 atoms including Pt and Cl^{-}) is 0.35 Å. The N···Pt, C···Pt, and O···Pt distances of **2c**·Cl^{-} are 2.01, 1.95, and 2.00/2.06 Å, respectively. The angles around Pt^{II} are 80.5°, 94.5°, 92.5°, and 92.6° with τ_4 value of 0.09.[S1] Atom color code: brown, pink, blue, red, green, yellow green (spherical), and gray refer to carbon, hydrogen, nitrogen, oxygen, fluorine, chlorine, and platinum, respectively.

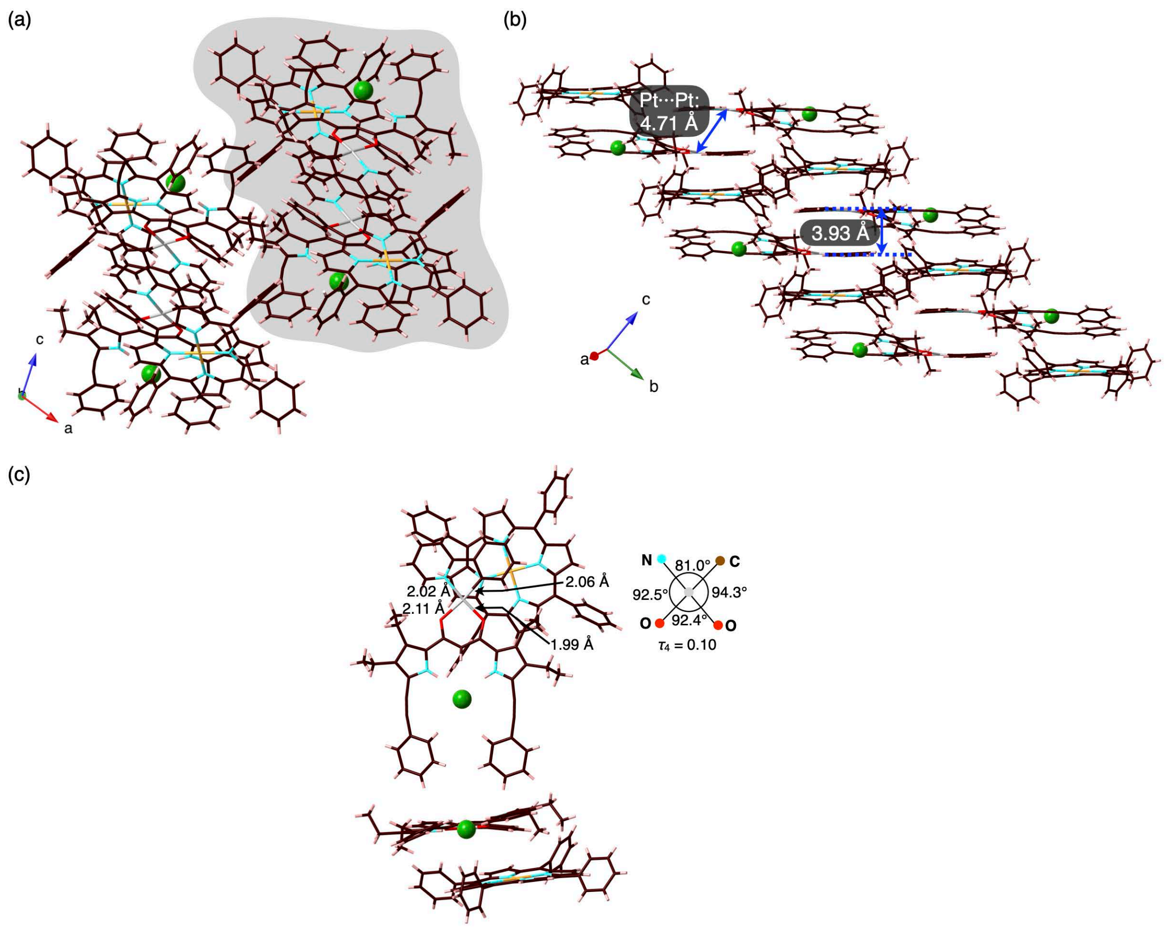

Figure S13 Packing diagrams of **2b**·Cl⁻-TPPAu⁺ which forms a charge-by-charge assembly: (a) the packing structure viewed from the top view, (b) packing structure as charge-by-charge assembly, and (c) ion pair **2b**·Cl⁻-TPPAu⁺. The stacking distance between two phenylpyridine mean planes (12 atoms of phenylpyridine) is 3.93 Å. The closest Pt···Pt distance is 4.71 Å, indicating the absence of Pt···Pt interaction. The distance between receptor mean planes (core 33 atoms including Pt and Cl⁻) and TPPAu⁺ (core 25 atoms) is 3.91 Å. **2b**·Cl⁻ exhibits [1+1]-type anion complex through hydrogen-bonding interactions with N(–H)···Cl⁻, $C_{bridging}$(–H)···Cl⁻, and C_{phenyl}(–H)···Cl distances of 3.19/3.18, 3.68, and 3.81/3.86 Å, respectively. Mean-plane deviation of the receptor unit (core 33 atoms including Pt and Cl⁻) is 0.20 Å. The N···Pt, C···Pt, and O···Pt distances of **2b**·Cl⁻ are 2.02, 2.06, and 1.99/2.11 Å, respectively. The angles around Pt^{II} are 81.0°, 94.3°, 92.4°, and 92.5° with τ_4 value of 0.10.[S1] Atom color code: brown, pink, blue, red, yellow green (spherical), gray, and orange refer to carbon, hydrogen, nitrogen, oxygen, chlorine, platinum, and gold, respectively.

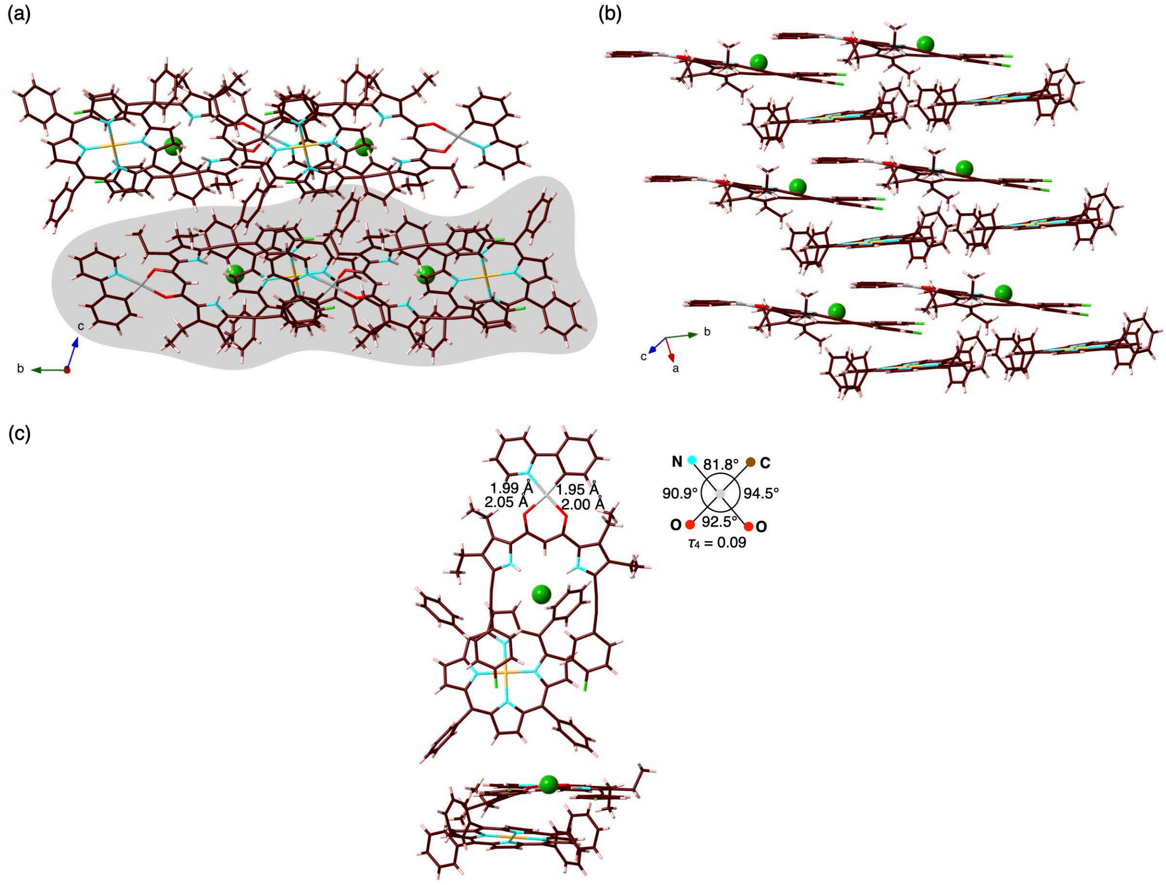

Figure S14 Packing diagrams of **2c**·Cl$^-$-TPPAu$^+$ which forms a charge-by-charge assembly: (a) the packing structure viewed from the top view, (b) packing structure as charge-by-charge assembly, and (c) ion pair **2c**·Cl$^-$-TPPAu$^+$. The closest Pt···Au distances is 4.91 Å. **2c**·Cl$^-$ exhibits [1+1]-type anion complex through hydrogen-bonding interactions with N(–H)···Cl$^-$, $C_{bridging}$(–H)···Cl$^-$, and C_{phenyl}(–H)···Cl distances of 3.09/3.12, 3.60, and 3.79/3.94 Å, respectively. Mean-plane deviation of the receptor unit (core 33 atoms including Pt and Cl$^-$) is 0.28 Å. The N···Pt, C···Pt, and O···Pt distances for Pt^{II} complex are 1.99, 1.95, and 2.00/2.05 Å, respectively. The angles around Pt^{II} are 81.8°, 90.9°, 92.5°, and 94.5° with τ_4 value of 0.09.[S1] Atom color code: brown, pink, blue, red, green, yellow green (spherical), gray, and orange refer to carbon, hydrogen, nitrogen, oxygen, fluorine, chlorine, platinum, and gold, respectively.

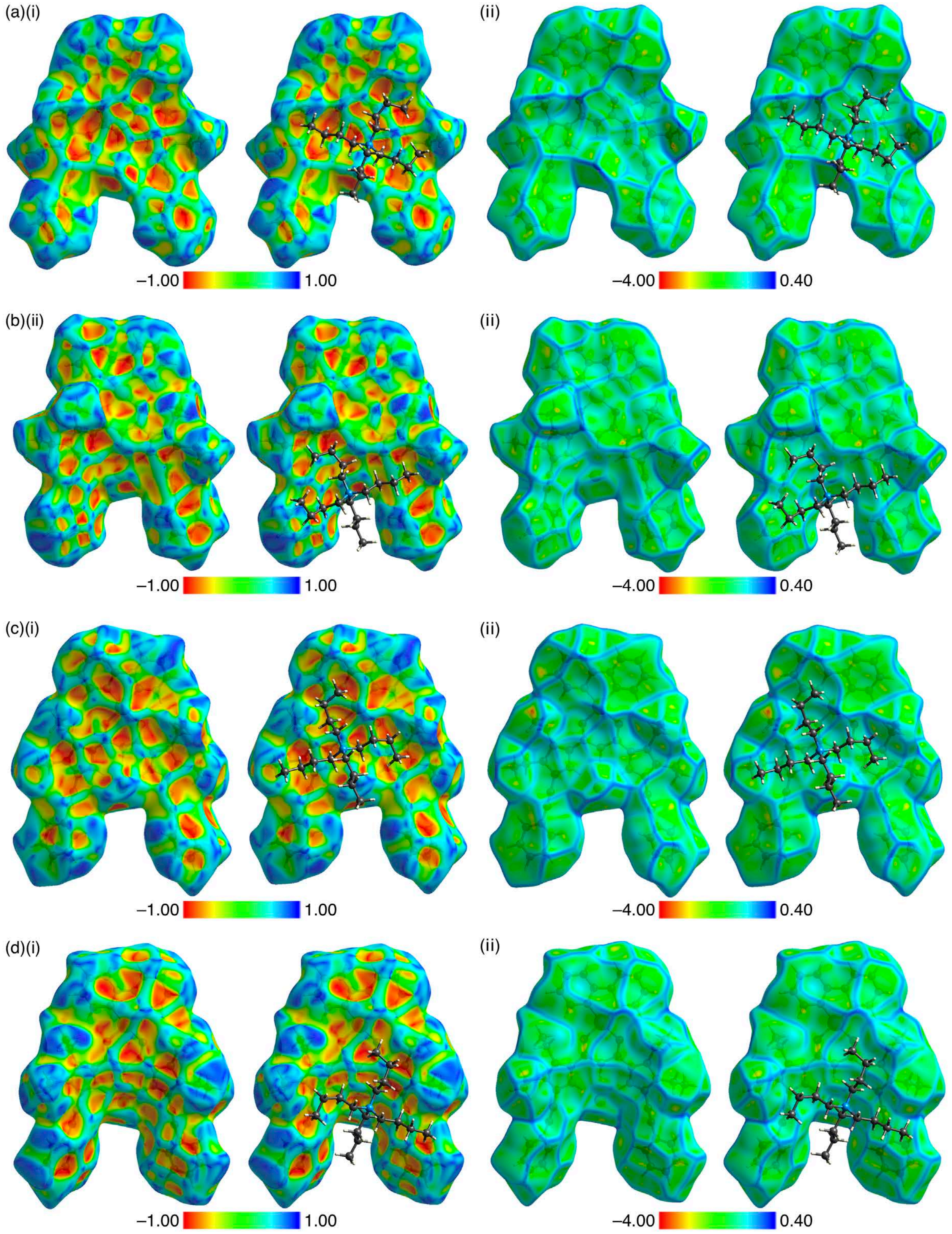

Figure S15 Hirshfeld surfaces[2,3] of **2b**·Cl⁻ (independent structures (a,b) and (c,d), wherein (b,d) show different sides of (a,c)) in the crystal structure of **2b**·Cl⁻-TBA⁺ mapped over (i) shape-index and (ii) curvedness properties for only surface (left) and surface with a ball-and-stick model of the neighboring TBA⁺ (right). Shape index is a qualitative measure of shape and is sensitive to subtle changes in surface shape, particularly in a flat region by differing by signs representing complementary bumps (blue) and hollows (red), whereas curvedness is a function of the root-mean-square curvature of the surface, and maps of curvedness typically show large regions of green (relatively flat) separated by dark blue edges (large positive curvature). The flat region on the curvedness surface suggested the characteristic mapping pattern for stacking between TBA⁺ and **2b**·Cl⁻.

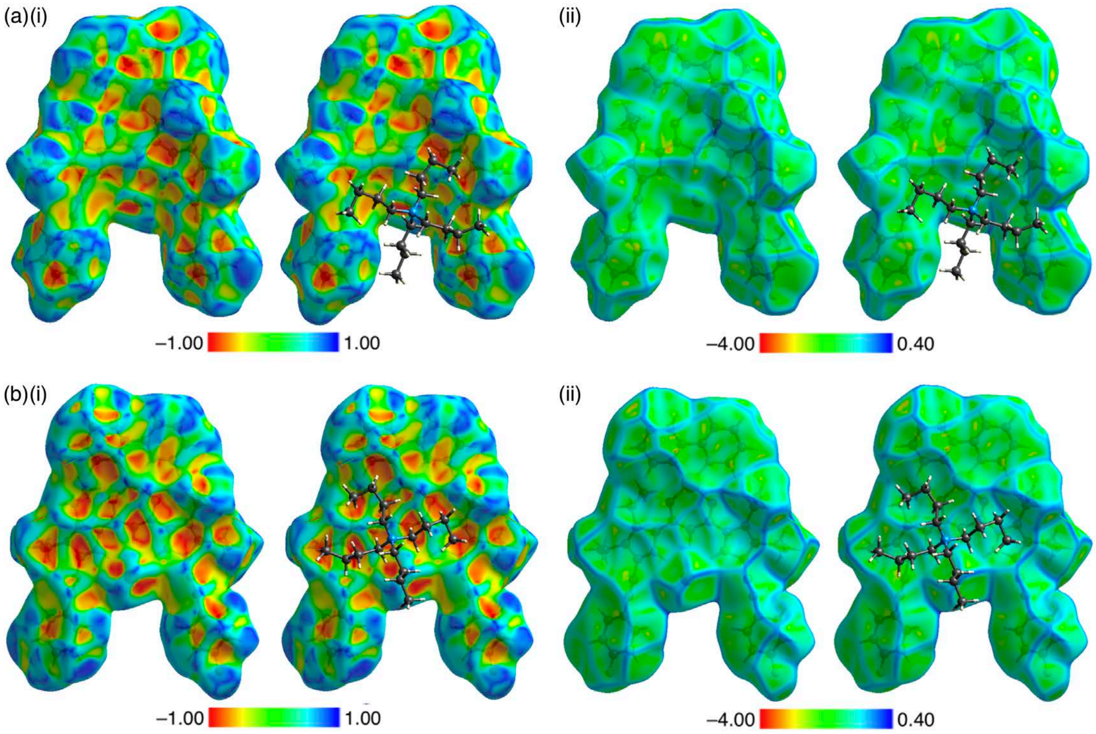

Figure S16 Hirshfeld surfaces[2,3] of **2c**·Cl⁻ ((a,b) for different sides) in the crystal structure of **2c**·Cl⁻-TBA⁺ mapped over (i) shape-index and (ii) curvedness properties for only surface (left) and surface with a ball-and-stick model of the neighboring TBA⁺ (right). Shape index is a qualitative measure of shape and is sensitive to subtle changes in surface shape, particularly in a flat region by differing by signs representing complementary bumps (blue) and hollows (red), whereas curvedness is a function of the root-mean-square curvature of the surface, and maps of curvedness typically show large regions of green (relatively flat) separated by dark blue edges (large positive curvature). The flat region on the curvedness surface suggested the characteristic mapping pattern for stacking between TBA⁺ and **2c**·Cl⁻.

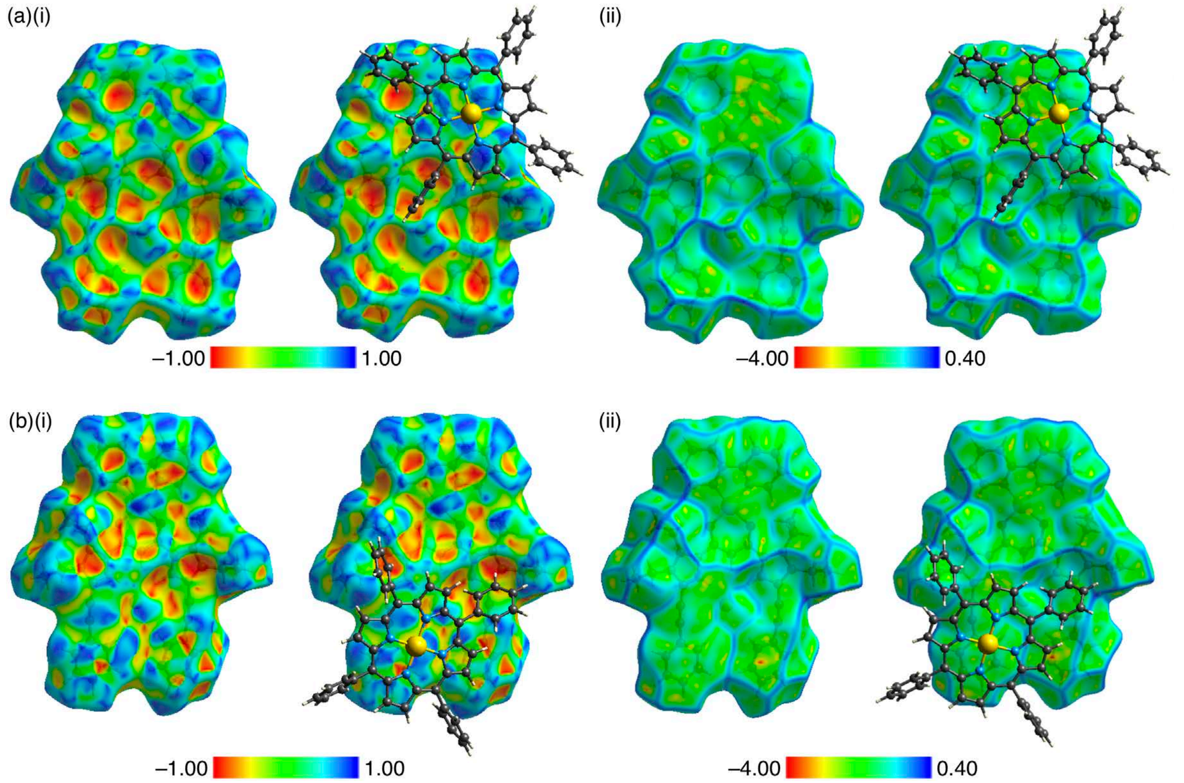

Figure S17 Hirshfeld surfaces[2,3] of **2b**·Cl^{-} ((a,b) for different sides) in the crystal structure of **2b**·Cl^{-}-TPPAu^{+} mapped over (i) shape-index and (ii) curvedness properties for only surface (left) and surface with a ball-and-stick model of the neighboring TPPAu^{+} (right). Shape index is a qualitative measure of shape and is sensitive to subtle changes in surface shape, particularly in a flat region by differing by signs representing complementary bumps (blue) and hollows (red), whereas curvedness is a function of the root-mean-square curvature of the surface, and maps of curvedness typically show large regions of green (relatively flat) separated by dark blue edges (large positive curvature). The flat region on the curvedness surface suggested the characteristic mapping pattern for stacking between TPPAu^{+} and **2b**·Cl^{-}.

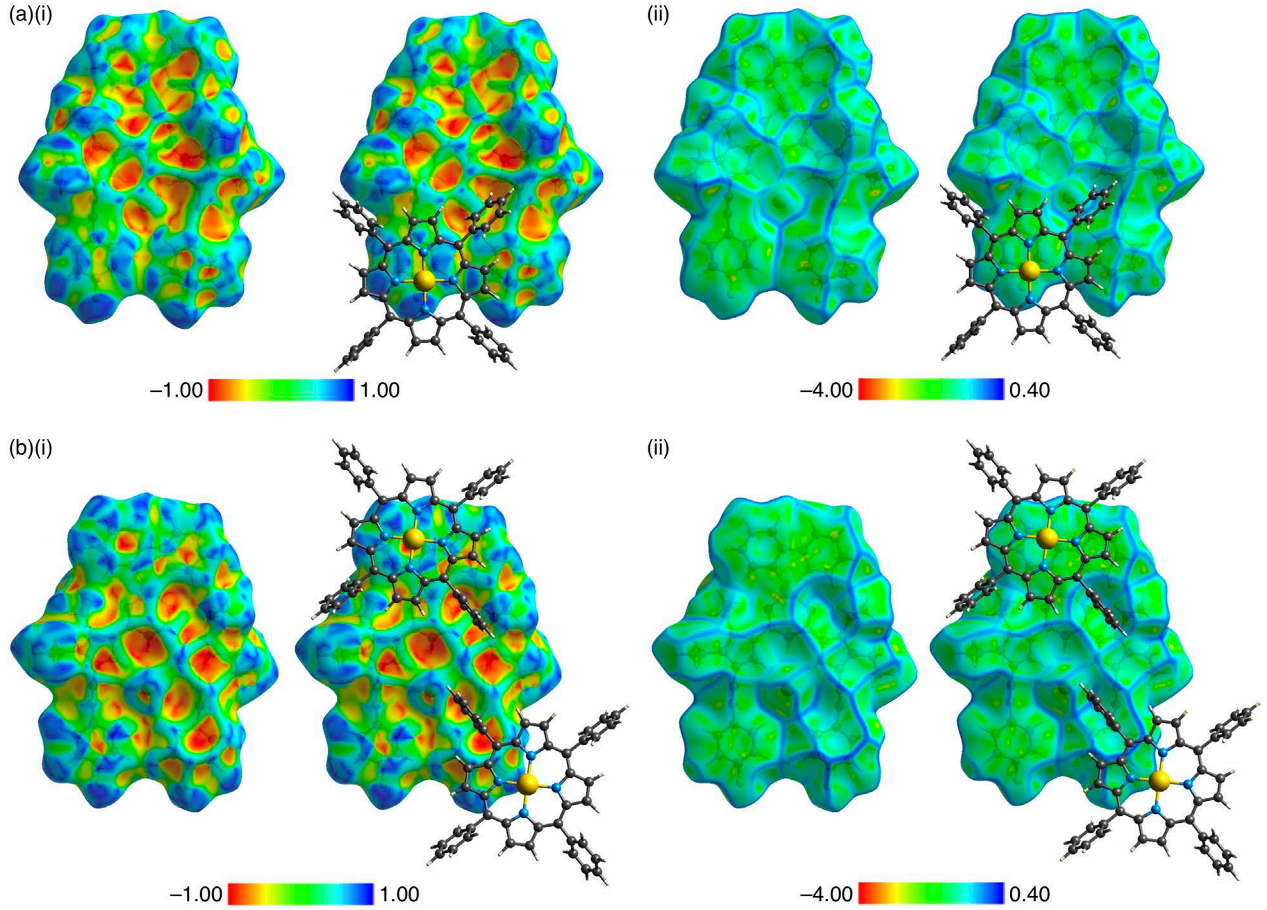

Figure S18 Hirshfeld surfaces[2,3] of **2c**·Cl^- ((a,b) for different sides) in the crystal structure of **2c**·Cl^--$TPPAu^+$ mapped over (i) shape-index and (ii) curvedness properties for only surface (left) and surface with a ball-and-stick model of the neighboring $TPPAu^+$ (right). Shape index is a qualitative measure of shape and is sensitive to subtle changes in surface shape, particularly in a flat region by differing by signs representing complementary bumps (blue) and hollows (red), whereas curvedness is a function of the root-mean-square curvature of the surface, and maps of curvedness typically show large regions of green (relatively flat) separated by dark blue edges (large positive curvature). The flat region on the curvedness surface suggested the characteristic mapping pattern for stacking between $TPPAu^+$ and **2c**·Cl^-.

[S1] Yang, L.; Powell, D. R.; Houser, R. P. Structural variation in copper(I) complexes with pyridylmethylamide ligands: structural analysis with a new four-coordinate geometry index, τ4. *Dalton Trans.* **2007**, 955–964.

[S2] Spackman, P. R.; Turner, M. J.; McKinnon, J. J.; Wolff, S. K.; Grimwood, D. J.; Jayatilaka, D.; Spackman, M. A. CrystalExplorer: a program for Hirshfeld surface analysis, visualization and quantitative analysis of molecular crystals. *J. Appl. Cryst.* **2021**, *54*, 1006–1011.

[S3] (a) Spackman, M. A.; Jayatilaka, D. Hirshfeld surface analysis. *CrystEngComm* **2009**, *11*, 19–32. (b) McKinnon, J. J.; Spackman, M. A.; Mitchell, A. S. Novel tools for visualizing and exploring intermolecular interactions in molecular crystals. *Acta Crystallogr. Sect. B* **2004**, *60*, 627–668.

3. Theoretical Studies

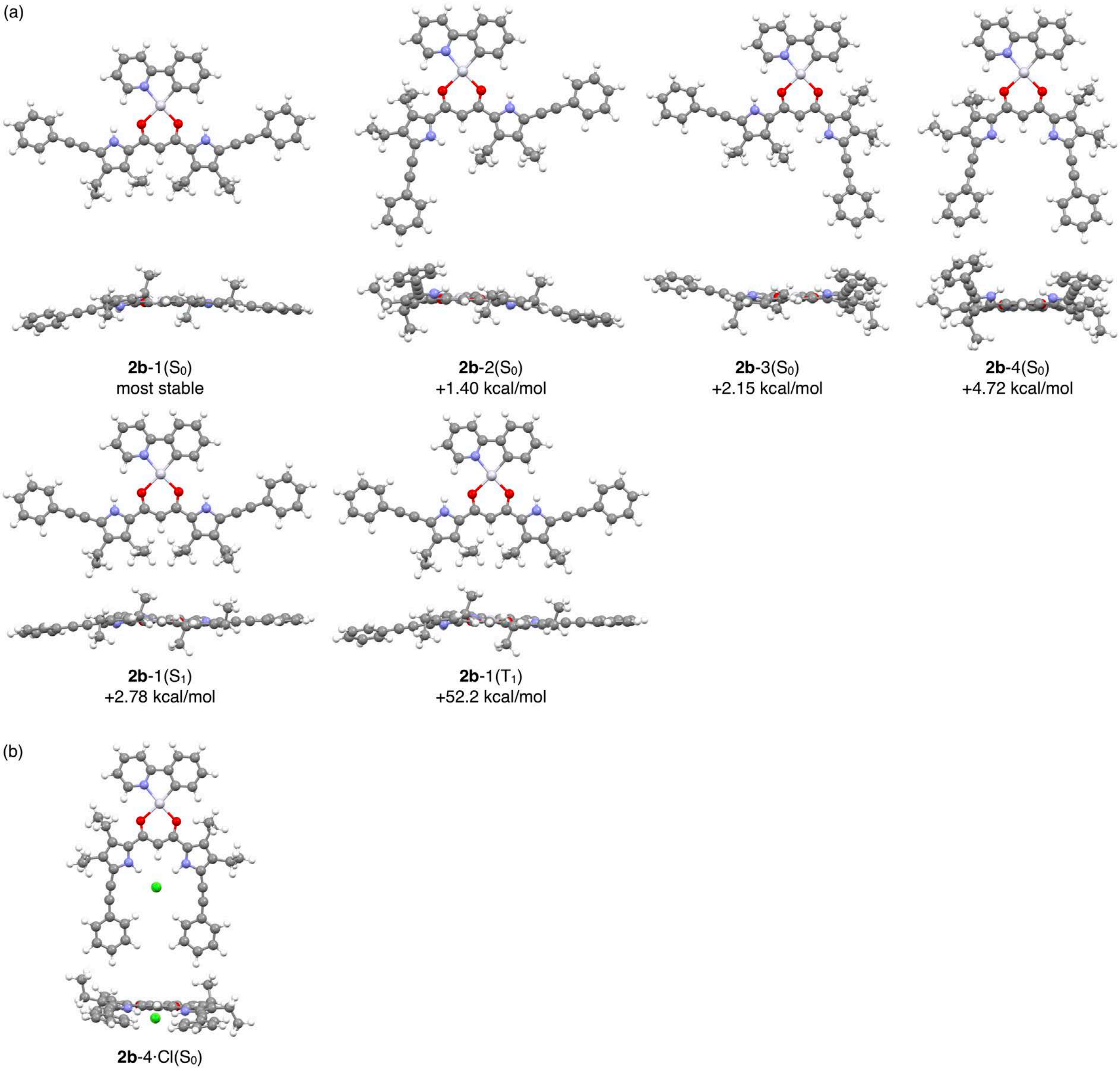

Figure S19 Optimized structures and relative energies of (a) **2b** (four conformations) in the ground (S_0) state and singlet (S_1) and triplet (T_1) excited states and (b) **2b**$\cdot Cl^-$ in the ground (S_0) state. The ground states (S_0) and excited triplet states (T_1) were optimized at the CAM-B3LYP level by using 6-31+G(d,p) basis set for C, H, N, O, and Cl and the LanL2DZ basis set and associated effective core potentials for Pt, whereas the excited singlet states (S_1) were optimized using the TD-DFT calculations at the same level to the ground states.[S4]

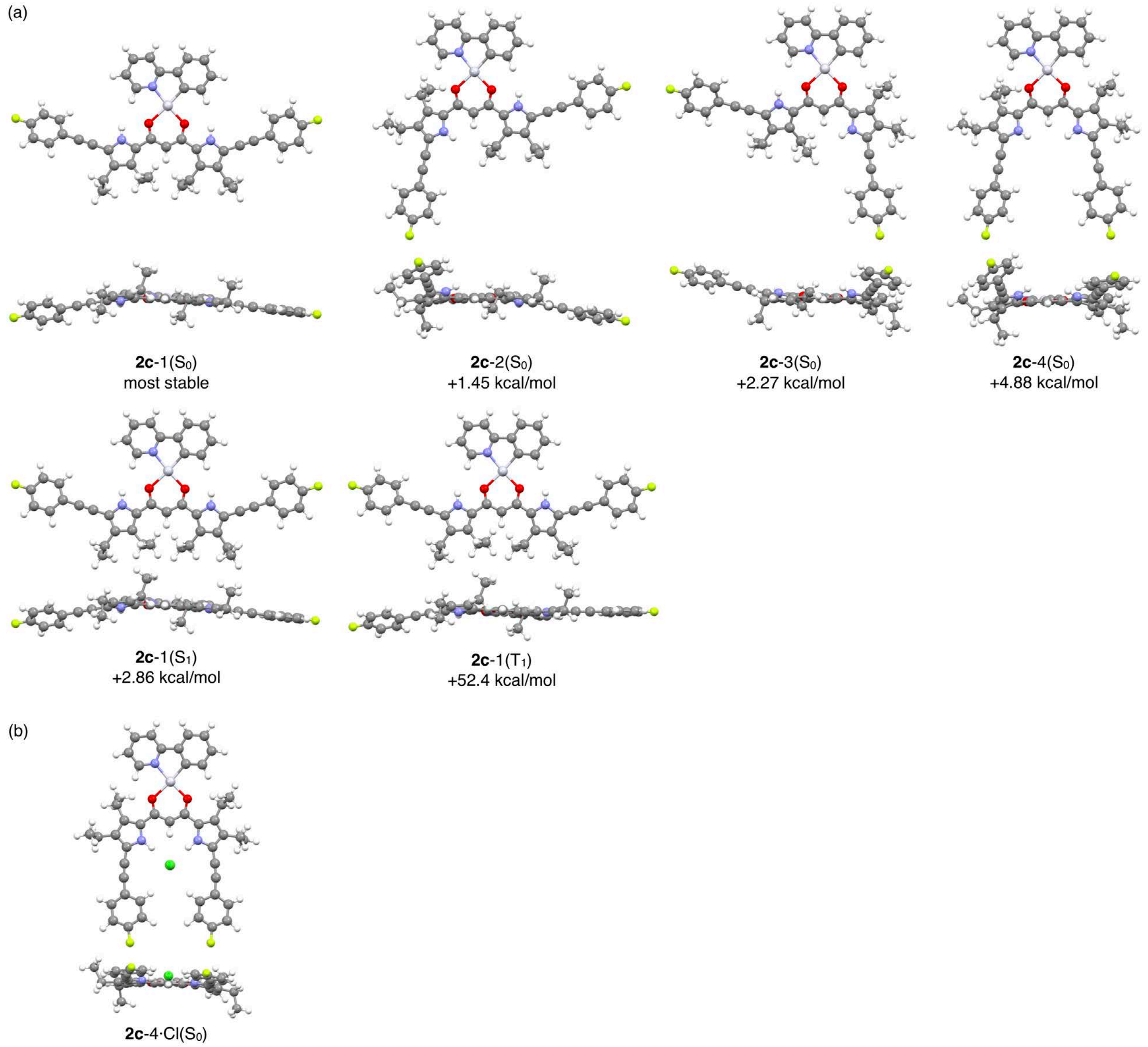

Figure 20 Optimized structures and relative energies of (a) **2c** (four conformations) in the ground (S_0) state and singlet (S_1) and triplet (T_1) excited states and (b) **2c**·Cl^- in the ground (S_0) state. The ground states (S_0) and excited triplet states (T_1) were optimized at the CAM-B3LYP level by using 6-31+G(d,p) basis set for C, H, N, O, F, and Cl and the LanL2DZ basis set and associated effective core potentials for Pt, whereas the excited singlet states (S_1) were optimized using the TD-DFT calculations at the same level to the ground states.[S4]

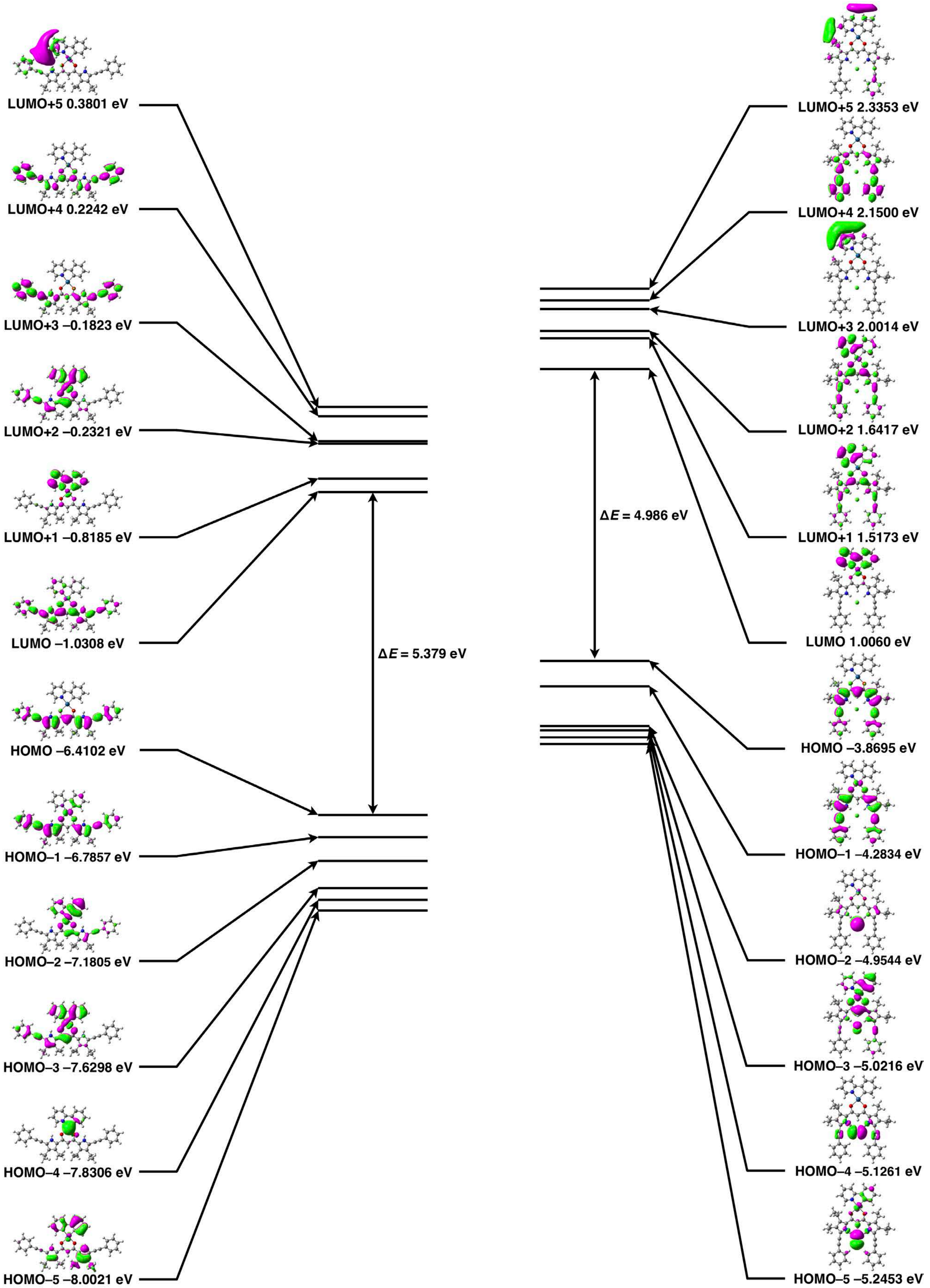

Figure S21 Molecular orbitals (HOMO and LUMO) of **2b** (left) and **2b**·Cl^- (right) estimated at the PCM-B3LYP level by using 6-31+G(d,p) basis set was used for C, H, N, O, and Cl and the LanL2DZ basis set and associated effective core potentials were used for Pt in solution phase (CH_2Cl_2).[S4]

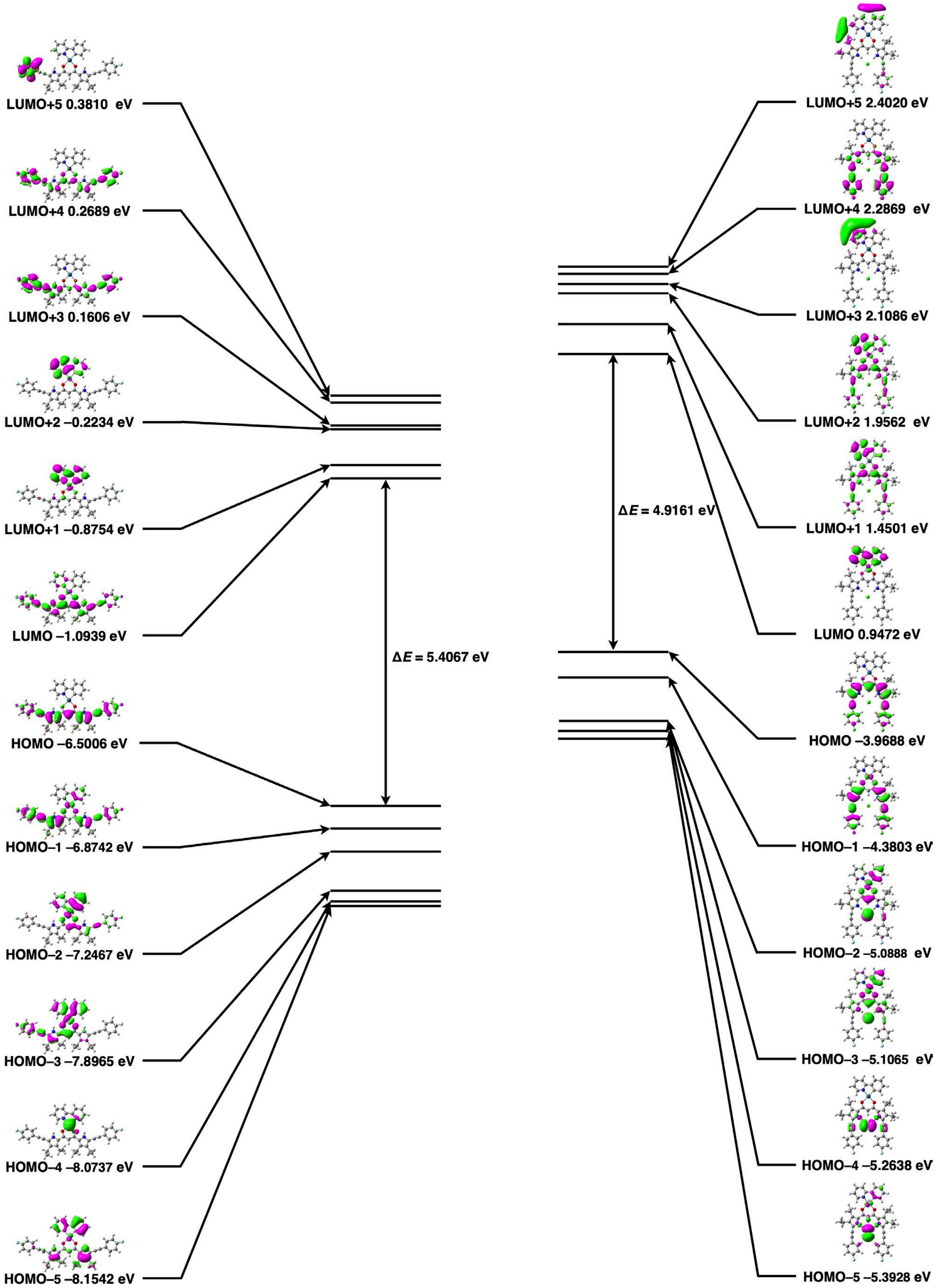

Figure S22 Molecular orbitals (HOMO and LUMO) of **2c** (left) and **2c**·Cl^- (right) estimated at the PCM-B3LYP level by using 6-31+G(d,p) basis set was used for C, H, N, O, F, and Cl and the LanL2DZ basis set and associated effective core potentials were used for Pt in solution phase (CH_2Cl_2).[S4]

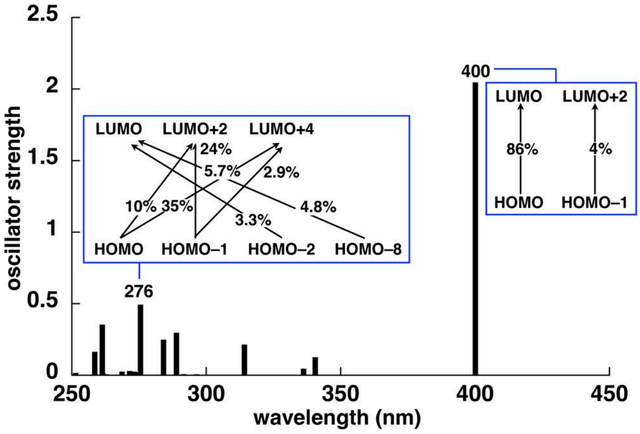

Figure S23 TD-DFT-based UV/vis absorption stick spectrum of **2b** with the transitions correlated with molecular orbitals estimated at the PCM-B3LYP level by using 6-31+G(d,p) basis set was used for C, H, N, and O and the LanL2DZ basis set and associated effective core potentials were used for Pt in solution phase (CH_2Cl_2).[S4]

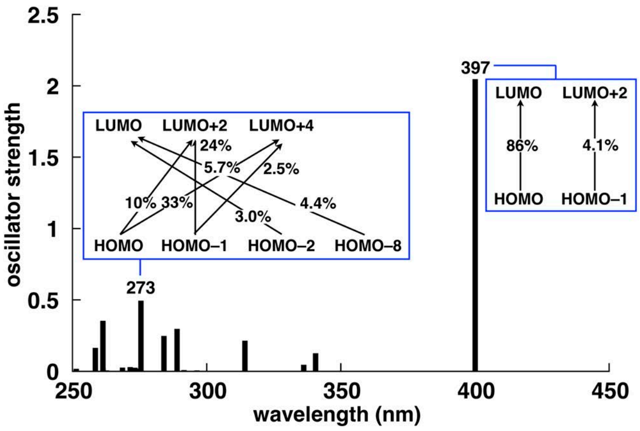

Figure S24 TD-DFT-based UV/vis absorption stick spectrum of **2c** with the transitions correlated with molecular orbitals estimated at the PCM-B3LYP level by using 6-31+G(d,p) basis set was used for C, H, N, O, and F and the LanL2DZ basis set and associated effective core potentials were used for Pt in solution phase (CH_2Cl_2).[S4]

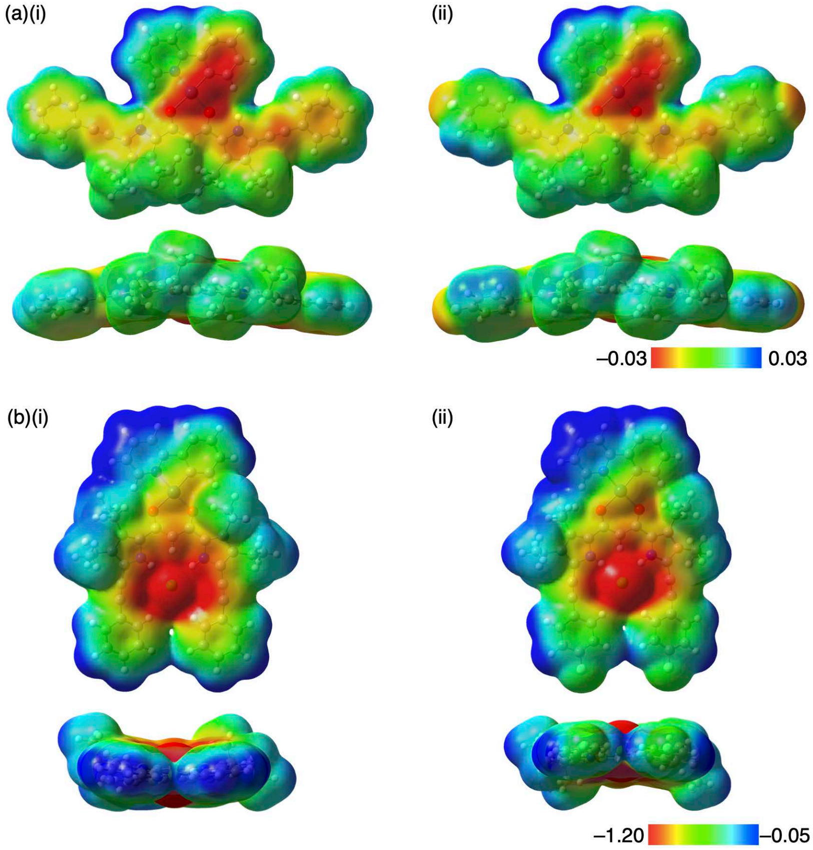

Figure S25 ESP mapping (δ = 0.0004) of (a) Pt^{II} complexes (i) **2b** and (ii) **2c** and (b) their Cl^- complexes calculated at the CAM-B3LYP level by using 6-31+G(d,p) basis set was used for C, H, N, O, F, and Cl and the LanL2DZ basis set and associated effective core potentials were used for Pt in solution phase.[S4]

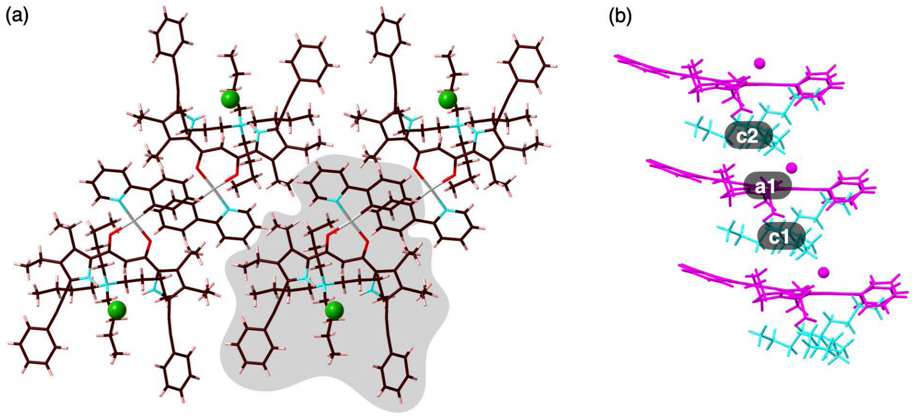

Figure S26 Single-crystal X-ray structure of **2b**·Cl^--TBA^+ for the EDA calculations (Table S1): (a) top view of charge-by-charge structure and (b) side view of shaded part in (a). The labels (c1,2 and a1) correspond to the fragments shown in Table S1.

Table S1 Energies between selected fragments in **2b**·Cl^--TBA^+ (Figure S26) estimated by EDA calculation[S5] based on an FMO2-MP2 using mixed basis sets including NOSeC-V-TZP for Pt and NOSeC-V-DZP for the other atoms.[S6–8]

fragments	total interaction energy (E_{tot}) (kcal/mol)	electrostatic interaction energy (E_{es}) (kcal/mol)	dispersion interaction energy (E_{disp}) (kcal/mol)	exchange repulsion interaction energy (E_{ex}) (kcal/mol)	charge-transfer interaction energy (E_{ct+mix}) (kcal/mol)
c1-a1	–170.512	–86.266	–95.174	19.682	–8.753
c2-a1	–162.399	–89.726	–83.085	18.327	–7.915
c1-c2	41.140	41.140	0.000	0.000	0.000

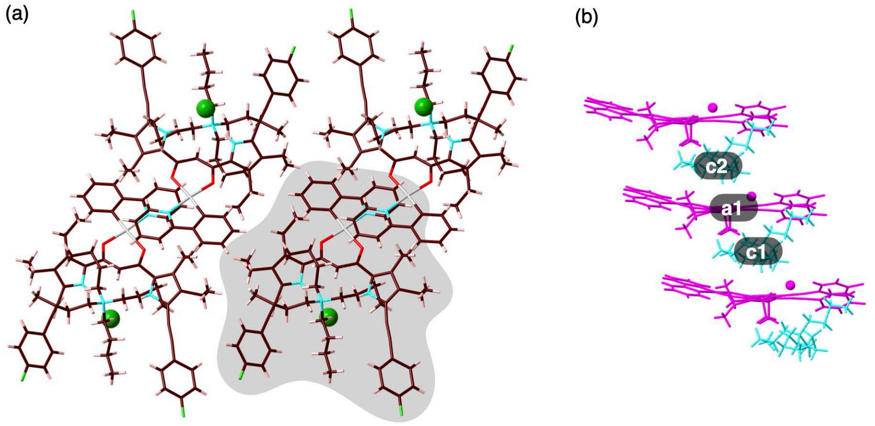

Figure S27 Single-crystal X-ray structure of **2c**·Cl⁻-TBA⁺ for the EDA calculations (Table S2): (a) top view of structure and (b) side view of shaded part in (a). The labels (c1,2 and a1) correspond to the fragments shown in Table S2.

Table S2 Energies between selected fragments in **2c**·Cl⁻-TBA⁺ (Figure S27) estimated by EDA calculation[S5] based on an FMO2-MP2 using mixed basis sets including NOSeC-V-TZP for Au and Pt and NOSeC-V-DZP for the other atoms.[S6–8]

fragments	total interaction energy (E_{tot}) (kcal/mol)	electrostatic interaction energy (E_{es}) (kcal/mol)	dispersion interaction energy (E_{disp}) (kcal/mol)	exchange repulsion interaction energy (E_{ex}) (kcal/mol)	charge-transfer interaction energy (E_{ct+mix}) (kcal/mol)
c1-a1	–157.747	–78.559	–87.808	16.262	–7.642
c2-a1	–169.380	–93.726	–91.909	28.453	–12.198
c1-c2	40.020	40.020	0.000	0.000	0.000

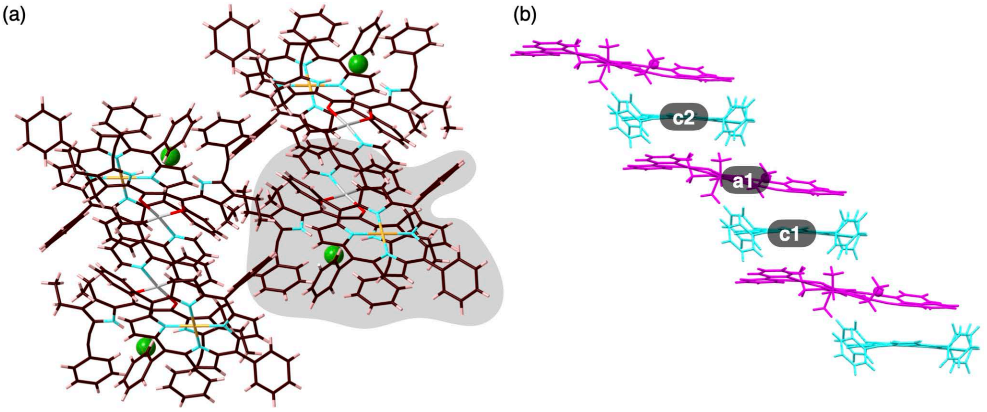

Figure S28 Single-crystal X-ray structure of **2b**·Cl⁻-TPPAu⁺ for the EDA calculations (Table S3): (a) top view of structure and (b) side view of shaded part in (a). The labels (c1,2 and a1) correspond to the fragments shown in Table S3.

Table S3 Energies between selected fragments in **2b**·Cl⁻-TPPAu⁺ (Figure S28) estimated by EDA calculation[S5] based on an FMO2-MP2 using mixed basis sets including NOSeC-V-TZP for Au and Pt and NOSeC-V-DZP for the other atoms.[S6–8]

fragments	total interaction energy (E_{tot}) (kcal/mol)	electrostatic interaction energy (E_{es}) (kcal/mol)	dispersion interaction energy (E_{disp}) (kcal/mol)	exchange repulsion interaction energy (E_{ex}) (kcal/mol)	charge-transfer interaction energy (E_{ct+mix}) (kcal/mol)
c1-a1	–203.082	–61.877	–155.574	25.844	–11.476
c2-a1	–163.537	–54.911	–119.056	19.274	–8.845
c1-c2	25.320	25.320	0.000	0.000	0.000

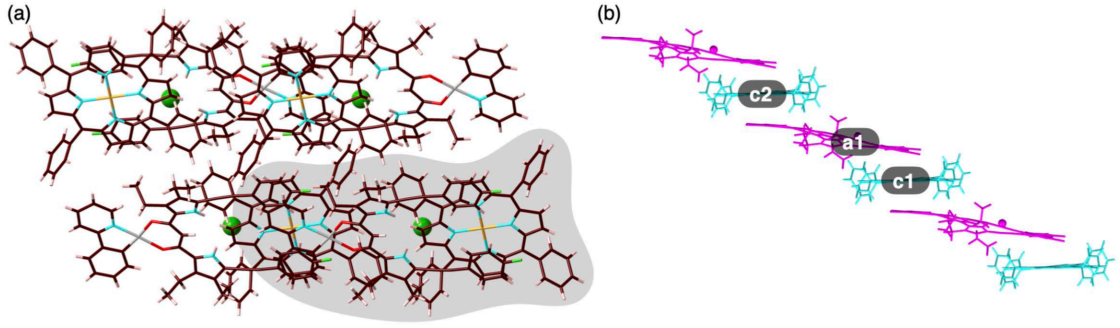

Figure S29 Single-crystal X-ray structure of **2c**·Cl⁻-TPPAu⁺ for the EDA calculations (Table S4): (a) top view of structure and (b) side view of shaded part in (a). The labels (c1,2 and a1) correspond to the fragments shown in Table S4.

Table S4 Energies between selected fragments in **2c**·Cl^--$TPPAu^+$ (Figure S29) estimated by EDA calculation[S5] based on an FMO2-MP2 using mixed basis sets including NOSeC-V-TZP for Au and Pt and NOSeC-V-DZP for the other atoms.[S6–8]

fragments	total interaction energy (E_{tot}) (kcal/mol)	electrostatic interaction energy (E_{es}) (kcal/mol)	dispersion interaction energy (E_{disp}) (kcal/mol)	exchange repulsion interaction energy (E_{ex}) (kcal/mol)	charge-transfer interaction energy (E_{ct+mix}) (kcal/mol)
c1-a1	–167.987	–56.990	–127.411	28.183	–11.768
c2-a1	–186.461	–61.929	–152.355	45.647	–17.824
c1-c2	20.142	20.142	0.000	0.000	0.000

Cartesian Coordination and Heat of Formation

2b-1(S_0)
(CAM-B3LYP/6-31+G(d,p) for C, H, N, and O and CAM-B3LYP/LanL2DZ for Pt)
-2210.3015706 hartree
C,-0.879837817,4.5514869967,-0.2773236792
C,-1.7037477886,5.6808128579,-0.271168465
H,-1.2789459234,6.6795723412,-0.3093111897
C,-3.0816466659,5.5286339753,-0.2178208613
H,-3.7270676989,6.4008244964,-0.213173084
C,-3.6290032007,4.2451374016,-0.1732649081
H,-4.7078032479,4.1227132096,-0.1362847078
C,-2.8069145559,3.1227811059,-0.1773770557
H,-3.2492827233,2.1335027734,-0.146825346
C,1.3092130578,-0.9163343572,-0.2229796683
C,0.0660985914,-1.5678054337,-0.1876260158
C,-1.1951462657,-0.96205438,-0.1184365103
C,-1.4172789844,3.2469137878,-0.2262286137
C,2.5003708002,3.2602454704,-0.4386293836
C,0.5793404285,4.6019600898,-0.3495058319
C,3.3295241321,4.3632751826,-0.5242147488
C,1.3728908921,5.7474844114,-0.4293917717
C,2.7499775159,5.6304456309,-0.5150882122
H,0.902592026,6.7229293102,-0.4280344224
H,3.3692977361,6.5192522473,-0.579256019
N,1.168425557,3.3775641616,-0.3473725299
Pt,-0.07452033,1.7986723529,-0.222397566
H,2.8721075255,2.2430117726,-0.4478509942
H,4.40177245,4.2280828399,-0.5995855626
H,0.0873699332,-2.6422578688,-0.1584738357
O,1.487774074,0.3526547568,-0.2027937068
O,-1.4374834591,0.3040064972,-0.1156515664
C,-2.4207191132,-1.7651931898,-0.0225202624
C,-2.7437126732,-3.1282956969,-0.0359781952
N,-3.591846951,-1.0711234637,0.1152421278
C,-4.1503669562,-3.2248610737,0.1054806149
H,-3.619431025,-0.0635865697,0.1529459836
C,2.5603576085,-1.6898828668,-0.2451791687
C,2.9252012737,-3.0152978716,-0.5079566753
N,3.7082171967,-0.9966943356,0.0249795972
C,4.3364169544,-3.0915787028,-0.3845985736
H,3.707784571,-0.0143068335,0.249642553
C,5.2053296002,-4.303405122,-0.5506148123
H,4.8427500811,-4.9069186517,-1.3900486722
H,6.2142830341,-3.9780001684,-0.8240251356
C,2.0588754751,-4.1686858551,-0.9328721887
H,1.2116298403,-4.28619354,-0.251460736
H,2.6386734063,-5.0903076566,-0.8287748179
C,-1.8376158261,-4.323237369,-0.1359678461
H,-1.0371110193,-4.1377496037,-0.8570858172
H,-2.4111588732,-5.1577137601,-0.5509073934
C,-4.9834238624,-4.4727450004,0.1158885905
H,-4.4389804413,-5.2766488667,0.6229758778
H,-5.8835018811,-4.2916625047,0.7126109517
C,-5.3958486104,-4.9389256608,-1.2866739608
H,-4.5199953125,-5.1468758446,-1.9088158804
H,-5.9994651726,-5.8506197888,-1.2352032793
H,-5.9845243024,-4.1682065891,-1.7922883463
C,-1.2496998793,-4.753167068,1.2150930081
H,-2.0484458029,-4.9915331521,1.9234413464
H,-0.6190142309,-5.641035339,1.1051178109
H,-0.645781953,-3.9573667154,1.6604898818
C,1.5592014565,-4.0590076468,-2.3790958717
H,2.4021579126,-3.9984396888,-3.0736579475
H,0.9569070089,-4.9313159313,-2.6520086122
H,0.9472719322,-3.1643513029,-2.5223121243
C,5.2820724077,-5.1738702526,0.7104574179
H,4.2916090228,-5.5338095084,1.0049394722
H,5.9244149663,-6.0449003851,0.5468777268

H,5.6891207659,-4.6028761261,1.5498393711
C,-5.9799590657,-1.4754730291,0.3495398962
C,-7.122727421,-1.0964452483,0.4833236828
C,6.1071015463,-1.3627459996,0.207632578
C,7.2353638164,-0.9766259046,0.4219987178
C,8.5676326332,-0.5266647583,0.6759614116
C,9.6633190289,-1.3570835898,0.396993315
C,8.7995347128,0.7502402632,1.2085023413
C,10.9571686871,-0.9175804815,0.6464303929
H,9.4885597815,-2.3449311703,-0.0155749374
C,10.0965565834,1.1829106024,1.453514642
H,7.9549910322,1.394073869,1.4299397662
C,11.1794486257,0.3523125713,1.1742237986
H,11.7965199643,-1.5698347274,0.4268383263
H,10.2628268582,2.1728090404,1.8666889959
H,12.1916360473,0.6927057445,1.3676306805
C,-8.4707899409,-0.6497154877,0.640123314
C,-9.5220393308,-1.575164766,0.7191956954
C,-8.7620050953,0.7203431325,0.7168942954
C,-10.8313200448,-1.1368695361,0.8711325503
H,-9.3008547288,-2.6354898872,0.6604891127
C,-10.0739919395,1.150372618,0.8689551319
H,-7.9517383649,1.4390084664,0.6569948964
C,-11.1127558888,0.2254001245,0.9465441561
H,-11.6361559531,-1.8627226716,0.9310873951
H,-10.2865689338,2.2131987363,0.9274049818
H,-12.1368034897,0.5645893436,1.065445511
C,-4.6484436931,-1.9263407808,0.1972441952
C,4.793001886,-1.8198944011,-0.0445936017

2b-2(S_0)

(CAM-B3LYP/6-31+G(d,p) for C, H, N, and O and CAM-B3LYP/LanL2DZ for Pt)

-2210.2993425 hartree
C,2.4427930986,-1.3030919023,-4.1726041164
C,2.2957136471,-1.2992624336,-5.5626555408
H,2.9374404025,-1.9092345611,-6.191608693
C,1.3164998704,-0.5111211958,-6.1501500859
H,1.1963078584,-0.5043620582,-7.228523568
C,0.4845070453,0.2662892954,-5.3424124373
H,-0.2894530316,0.8766012476,-5.7993483803
C,0.6340551868,0.2633394321,-3.9590214104
H,-0.0285789181,0.8623792254,-3.3441920055
C,1.9163472682,-0.3845405839,1.6137985576
C,0.8987290163,0.5860764977,1.4984356147
C,0.3051905286,1.0561608058,0.3232510609
C,1.6169696403,-0.5138761856,-3.3434860912
C,4.2033440112,-2.6482813618,-1.3059475804
C,3.4263048183,-2.1114679396,-3.4532039537
C,5.1462490392,-3.5243467659,-1.8121496149
C,4.3617628293,-2.9823247334,-4.013133897
C,5.224501717,-3.6905808142,-3.1929832043
H,4.4051985275,-3.0986068751,-5.0889227006
H,5.952998155,-4.3688606803,-3.6253815623
N,3.372041108,-1.9637921889,-2.1034533131
Pt,1.9970615534,-0.6665957587,-1.412350534
H,4.079610661,-2.4637557342,-0.2461677193
H,5.8008646059,-4.0608654329,-1.1359092222
H,0.5979149782,1.0828436651,2.4055671395
O,2.5036691069,-0.9665240749,0.6439716292
O,0.5827485238,0.6840999588,-0.8780853005
C,-0.727729381,2.0978963374,0.371256321
C,-1.5187090669,2.6797441916,1.3684174599
N,-1.0761218791,2.6760773338,-0.8179734701
C,-2.3495884575,3.6376402102,0.734579375
H,-0.6487088436,2.4155734297,-1.6939692376
C,2.3605840803,-0.7564026804,2.9640534211
C,3.5473617984,-1.334334563,3.4147433684
N,1.5743338115,-0.5192067941,4.0656683115
C,3.457256756,-1.4413851712,4.8266730724
H,0.6179552166,-0.2039516398,4.0416193456
C,4.4773624339,-2.0097720515,5.7682183933
H,5.4811047206,-1.7044943208,5.4526370147
H,4.3238349165,-1.5733853346,6.7604286215
C,4.7451630768,-1.7268672558,2.5971382435
H,4.4294063685,-2.4044852764,1.7997901229
H,5.4270356121,-2.298899292,3.2337551619
C,-1.5443725465,2.3983427006,2.845032633
H,-1.3666236295,1.3335710524,3.0264981184
H,-2.555504201,2.5911497186,3.2173048492
C,-3.4018549129,4.4964588295,1.372165191
H,-3.0888070411,4.7736778438,2.3844579572
H,-3.4824885181,5.4334553977,0.8111943742
C,-4.7804111303,3.8247014002,1.4285614432
H,-4.7433109811,2.8905461383,1.9973100503
H,-5.5173261831,4.4810379864,1.9022222892
H,-5.1345879381,3.5845656683,0.4220807206
C,-0.5530193712,3.2480094834,3.6524100085
H,-0.7629815619,4.3134055276,3.5214539861
H,-0.6180567817,3.019816784,4.7210301482
H,0.4787161623,3.0807692631,3.330031335
C,5.5009115688,-0.538259489,1.9924081642
H,5.8381575208,0.1475818403,2.7754397208
H,6.3824691692,-0.8783913349,1.4392784856
H,4.8634303707,0.0194800305,1.3026052225
C,4.4179266061,-3.5390861839,5.8752490534
H,4.5851645704,-4.0109769852,4.9023255944
H,5.177947296,-3.9129098031,6.5685600733
H,3.437811165,-3.8638130025,6.236209706
C,-2.5848231735,4.3774306194,-1.6868943205
C,-3.0466544257,5.037621166,-2.5914954053
C,1.6149222418,-0.8469429286,6.4766881016
C,1.104787858,-0.7672716674,7.5727584169
C,0.5075332585,-0.676052947,8.867642642
C,1.2182676836,-1.0804009099,10.0074757548
C,-0.7965908621,-0.1816693971,9.0176305881
C,0.6349255324,-0.991070244,11.2648760809
H,2.227160858,-1.4628497155,9.8957091247
C,-1.3726324028,-0.0955635418,10.2786443525
H,-1.3502152131,0.1313415805,8.1388535943
C,-0.6607189545,-0.4991010564,11.4059646592
H,1.1949662184,-1.3070597205,12.1393277189
H,-2.3826471849,0.288374174,10.381716781
H,-1.1135225392,-0.4305472144,12.389866413
C,-3.5911855491,5.8160682959,-3.6587792165
C,-4.6190328953,6.7386417562,-3.4135146653
C,-3.1057382473,5.6683854471,-4.9665758764
C,-5.1462339422,7.4930341379,-4.4538915594
H,-4.9968472862,6.855846814,-2.4034696941
C,-3.6386382998,6.4264068196,-6.0013919611

H,-2.309717818,4.95731354,-5.1599169658
C,-4.6594498252,7.340464369,-5.7502102412
H,-5.9413335285,8.2037241692,-4.2515253071
H,-3.2544597523,6.303309557,-7.009081588
H,-5.0734901574,7.9314063939,-6.5608998496
C,-2.0474123723,3.6131688895,-0.6255333701
C,2.2133545849,-0.9416924137,5.1992787224

2b-3(S_0)

(CAM-B3LYP/6-31+G(d,p) for C, H, N, and O and CAM-B3LYP/LanL2DZ for Pt)

-2210.298137 hartree
C,1.4161361837,0.658614088,-4.7809301915
C,1.3789665923,0.4779403787,-6.166960682
H,2.0889741111,0.9852959579,-6.8134879685
C,0.4255799127,-0.3598821341,-6.7267692378
H,0.391108442,-0.5054563401,-7.8015244813
C,-0.4857807053,-1.014275604,-5.8955429458
H,-1.23160591,-1.6736471886,-6.3309292423
C,-0.4480986798,-0.833666383,-4.516392062
H,-1.1561993004,-1.3516171857,-3.8831511478
C,0.4492990071,0.5398364904,1.0175823333
C,-0.6652139473,-0.3067845556,0.9503439325
C,-1.199829239,-0.9360618454,-0.1886051954
C,0.5000097171,0.0048299777,-3.9290400989
C,3.0188050373,2.2910250794,-1.9741337274
C,2.3756251731,1.5181419373,-4.0921749629
C,4.0230805375,3.0764914024,-2.5086896364
C,3.3776159885,2.2916829604,-4.6810078419
C,4.204377401,3.0714251799,-3.890341219
H,3.4978459912,2.2779231521,-5.7571068558
H,4.9829136113,3.6750527388,-4.34554995
N,2.2245926844,1.5319706926,-2.7418634233
Pt,0.7415574998,0.3844497088,-2.0062070477
H,2.8091514201,2.2461137525,-0.9127897476
H,4.6430411634,3.6785448511,-1.8553465323
H,-1.1778841464,-0.4852589071,1.8797663863
O,1.1770220051,0.886805985,0.0230190168
O,-0.8092008456,-0.7971845189,-1.4018937292
C,-2.3354050733,-1.8464123018,0.0149844343
C,-3.3677738486,-2.2407333926,-0.8342783213
N,-2.5782365157,-2.4078049193,1.2472229914
C,-4.2480667629,-3.0605361962,-0.0802756067
H,-1.9426781598,-2.3962428509,2.0287324726
C,0.8889750252,1.0880627565,2.3095447192
C,0.3450289644,1.1915970133,3.59434777
N,2.1183742934,1.6862438226,2.3369650265
C,1.2981473819,1.8680174061,4.3980660327
H,2.7163434697,1.7358365457,1.5272602461
C,1.1607846638,2.2587715339,5.8402094103
H,0.5864274601,1.4957472196,6.3763266568
H,2.1542933356,2.2717062605,6.3005542183
C,-0.9817322564,0.7011338165,4.1043984814
H,-1.7445039543,0.785150865,3.32537855
H,-1.3135846336,1.3642233319,4.9095100945
C,-3.5509345836,-1.8974694841,-2.283698723
H,-2.995297785,-0.987495177,-2.5178490703
H,-4.6092067671,-1.6833942536,-2.4718468983
C,-5.5470077363,-3.6600242894,-0.5316257747
H,-5.4855091688,-3.9141275789,-1.5942311149
H,-5.7106346022,-4.6026149604,0.0015516688
C,-6.7487636678,-2.7342147257,-0.299092342
H,-6.6257168155,-1.7867579722,-0.8322544079
H,-7.6756014045,-3.2011528007,-0.6470101713
H,-6.861103808,-2.50464988,0.7644007102
C,-3.0944230592,-3.0320602037,-3.2111804619
H,-3.6555601158,-3.9516508634,-3.0210538773
H,-3.2402759122,-2.7610598033,-4.2617111137
H,-2.0353773391,-3.2546737934,-3.0567193759
C,-0.9323732798,-0.7355145293,4.6430314814
H,-0.2427769479,-0.8103397373,5.4888044227
H,-1.9204714398,-1.064081138,4.9799284207
H,-0.5737082579,-1.4351420992,3.8812163147
C,0.4979195728,3.6288716115,6.0359220337
H,-0.5063984191,3.6489716158,5.602093058
H,0.4128767951,3.87480733,7.0990947272
H,1.083491256,4.4144155543,5.5498711137
C,-4.185072756,-3.8851327573,2.3237563478
C,-4.5833466256,-4.511110258,3.2816128381
C,3.60932516,2.80699535,3.9021833916
C,4.6508703481,3.3595562398,4.181144897
C,5.8771850842,4.0128936229,4.5146798682
C,6.0484331157,4.6007624112,5.7768282826
C,6.9272323785,4.074946517,3.5866158294
C,7.2419188741,5.2343682109,6.0985825045
H,5.2389945294,4.5549994283,6.4974767428
C,8.1172182566,4.711409951,3.9157352616
H,6.800638385,3.618121215,2.6108764014
C,8.2797102511,5.2929134676,5.1711182084
H,7.3623052357,5.6851306659,7.0786185595
H,8.9226516521,4.7520753769,3.1892009213
H,9.2109170436,5.7887063127,5.4257348108
C,-5.0573709982,-5.2496583546,4.4092081418
C,-6.2438146302,-5.9932584854,4.3235152597
C,-4.3437920749,-5.2421122176,5.6169158159
C,-6.702229088,-6.7098105445,5.4215949439
H,-6.7980540683,-6.0022200735,3.3911229889
C,-4.8090031849,-5.961558491,6.7102740468
H,-3.4248740723,-4.6699001986,5.6870822675
C,-5.9883045042,-6.697263965,6.6177719207
H,-7.6216109106,-7.2813995684,5.3428582596
H,-4.2475843847,-5.948232258,7.6391609099
H,-6.3490590231,-7.2585740443,7.4736974412
C,-3.720359951,-3.1610406393,1.201829129
C,2.3932979292,2.1576354629,3.587605252

2b-4(S_0)

(CAM-B3LYP/6-31+G(d,p) for C, H, N, and O and CAM-B3LYP/LanL2DZ for Pt)

-2210.2940412 hartree
C,0.5503442085,-4.1730833084,-2.7057612283
C,0.2954961238,-5.0518819747,-3.7627426276
H,0.8510987124,-5.9798869629,-3.8608854706
C,-0.6798215739,-4.7392423059,-4.6986241563
H,-0.8831537252,-5.4168571444,-5.5214291175
C,-1.3978423347,-3.5488380327,-4.5697879102
H,-2.1658877296,-3.3037333913,-5.2982464377
C,-1.1421390343,-2.673972351,-3.5182268648
H,-1.7090570459,-1.7568249267,-3.4275933908
C,0.6338139621,0.2994693871,1.0953719624

C,-0.3655136345,1.053046296,0.4500868324
C,-1.0865296111,0.7095651551,-0.7097415269
C,-0.1632353977,-2.963050234,-2.5670029525
C,2.454041741,-3.5112050915,0.3037238502
C,1.5419791224,-4.4147049439,-1.659951266
C,3.2969677541,-4.5949342332,0.4695963886
C,2.3748841008,-5.5279382599,-1.538487425
C,3.2555105335,-5.6194427448,-0.4735840588
H,2.3231973314,-6.3157321208,-2.2796983602
H,3.9038772609,-6.4842270687,-0.3758375637
N,1.6079834841,-3.4242660698,-0.7322454268
Pt,0.3666489593,-1.8651607029,-1.0127143349
H,2.4235720924,-2.6769090485,0.9936574755
H,3.9672499641,-4.6305525598,1.3199708692
H,-0.5004050533,2.0619777807,0.8154935938
O,1.0824487204,-0.8277831118,0.7097428611
O,-0.9583561654,-0.3606868693,-1.4024755385
C,-2.0791977703,1.6726400407,-1.2059026323
C,-2.5997863516,1.8889659418,-2.4806575634
N,-2.5971287726,2.6438848973,-0.3803078749
C,-3.4518051434,3.0224237506,-2.4051298841
H,-2.4988657553,2.6693325473,0.6209787803
C,1.2388357008,0.8576947293,2.3147712263
C,2.5149468589,0.7126050255,2.8527673418
N,0.5410366497,1.7176073038,3.1260814513
C,2.5718336833,1.5109630897,4.0259434864
H,-0.4448785172,1.9097949035,3.0523188424
C,3.7324607937,1.6777754215,4.9614623886
H,4.6621500331,1.7497217905,4.3862322027
H,3.626191097,2.6303475458,5.4903531288
C,3.6646796357,-0.0632202774,2.2738502343
H,3.3555729564,-1.0959868166,2.0961487209
H,4.4667286844,-0.1046059132,3.0171594471
C,-2.3457343281,1.0950719622,-3.728623098
H,-1.4076402667,0.5460945447,-3.6296067125
H,-2.2273013509,1.7824092375,-4.5739855894
C,-4.176144355,3.6954346579,-3.5333110946
H,-4.4766742076,2.9491058076,-4.2749760536
H,-5.1012502805,4.1394717628,-3.1505169764
C,-3.340182016,4.7852211006,-4.2181150884
H,-2.4132555905,4.372528145,-4.6274360986
H,-3.8972697637,5.247287656,-5.0391823986
H,-3.068743877,5.5700986537,-3.5064211018
C,-3.4861475944,0.1141032958,-4.0340498563
H,-4.4366125026,0.6382184256,-4.1703647037
H,-3.2804791124,-0.4515810174,-4.9483034381
H,-3.6164390416,-0.5950601474,-3.2123600002
C,4.2142613666,0.5309432183,0.9717855042
H,4.542194195,1.5640442471,1.1217491802
H,5.0704103528,-0.0479464284,0.6111606446
H,3.4523447964,0.5270973633,0.1885540248
C,3.8520464755,0.5422905197,5.9861975593
H,3.9774006772,-0.425664166,5.4916239832
H,4.7108491206,0.6994600656,6.646321878
H,2.9522445394,0.483623261,6.605290486
C,-4.1414356996,4.5226482807,-0.4716710346
C,-4.7412827877,5.4368951534,0.0501184224
C,0.8397222558,2.9598171425,5.198422238
C,0.424672674,3.6904736464,6.0713887973
C,-0.0589581228,4.5514598533,7.1042177645
C,0.8179145509,5.0580051991,8.0749643802
C,-1.4160201958,4.9017070458,7.1632570479
C,0.3448972005,5.893744115,9.0785584115
H,1.8682382331,4.7904124365,8.0324106159
C,-1.8808568511,5.7386767947,8.1696828296
H,-2.0985126018,4.5099814317,6.4165610167
C,-1.0040402894,6.2376330387,9.1302839674
H,1.0333612123,6.2790900752,9.8239977941
H,-2.9334683698,6.0012877562,8.205305766
H,-1.3702862398,6.8909115755,9.9156924525
C,-5.4491718743,6.5186148602,0.6592144619
C,-6.4807717937,7.1706731941,-0.0324470071
C,-5.1218012237,6.9448663595,1.9548765778
C,-7.1650317735,8.2239476134,0.5606581455
H,-6.7375659822,6.8429976606,-1.0340801171
C,-5.8123121927,7.9976499248,2.5416380164
H,-4.3219131799,6.4461940855,2.4917688994
C,-6.8350298685,8.6409024889,1.8482405755
H,-7.960819547,8.7211597232,0.0150973037
H,-5.5489594827,8.3191723243,3.5443591213
H,-7.3719457083,9.4639142176,2.3088825082
C,-3.4440002572,3.4564369419,-1.0849416802
C,1.3247216599,2.1068554236,4.1806589734

2b-1(S_1)
(CAM-B3LYP/6-31+G(d,p) for C, H, N, and O and CAM-B3LYP/LanL2DZ for Pt)
-2210.2971365 hartree
C,0.8455930635,4.5871633446,-0.033459841
C,1.6550347225,5.7251358469,-0.0995006503
H,1.2203358387,6.7198839107,-0.064512355
C,3.0309718181,5.5873358505,-0.2124755196
H,3.6650488003,6.466387689,-0.2647925243
C,3.590421615,4.3087575579,-0.2605172977
H,4.6674171063,4.1979180286,-0.3525083569
C,2.7828701155,3.1781048211,-0.1943298204
H,3.2315806441,2.1920489377,-0.2399186832
C,-1.3023637,-0.9096950383,0.1350285513
C,-0.0393631559,-1.5445341152,0.0763438795
C,1.2260558295,-0.9320046484,-0.0382669396
C,1.3949716979,3.28636786,-0.078099099
C,-2.5140102047,3.2637653904,0.2513385416
C,-0.6112397562,4.6238286234,0.0862111968
C,-3.3531263042,4.3598760119,0.3365032322
C,-1.4148980097,5.7622224046,0.1654867123
C,-2.788303712,5.6326478314,0.2897315033
H,-0.9556558036,6.7424371206,0.1323283188
H,-3.4154089733,6.5160984822,0.3531212004
N,-1.1864192506,3.3929502636,0.125414729
Pt,0.0709630249,1.8253367329,0.0129477954
H,-2.8700760031,2.2414992098,0.2870566711
H,-4.4218963571,4.2152883173,0.4399331121
H,-0.0434549765,-2.6201524657,0.1107473124
O,-1.4894337526,0.3774913923,0.0837679135
O,1.4597883079,0.3551797135,-0.0777909958
C,2.4307762873,-1.7244471969,-0.0964092753
C,2.7664974885,-3.1130874013,-0.1728455078
N,3.6100303454,-1.0330848225,-0.0869965828
C,4.1615927008,-3.2012858998,-0.20100837
H,3.6265580155,-0.0245444895,-0.0427257871

C,-2.5137498613,-1.6920498753,0.2068151881
C,-2.8606483606,-3.0621259338,0.4105282454
N,-3.6884488398,-1.0067941327,0.0597855785
C,-4.2578469685,-3.1490242602,0.3681559579
H,-3.7010789477,-0.013386507,-0.1117082808
C,-5.1086537847,-4.3765052764,0.5063623263
H,-4.6757170354,-5.043795805,1.2591798794
H,-6.0914123944,-4.0820537168,0.8883091878
C,-1.9548761274,-4.221805954,0.7022743165
H,-1.141304684,-4.2686946592,-0.0288232992
H,-2.5219560569,-5.1459227472,0.5576922284
C,1.8472855515,-4.2934543421,-0.2785405206
H,1.0552029077,-4.233186959,0.4747540951
H,2.413943875,-5.1945508441,-0.0265082996
C,5.005953744,-4.4395834333,-0.2526144138
H,4.523296567,-5.1896783286,-0.8878754077
H,5.9576937205,-4.1964532372,-0.7358616677
C,5.2822236185,-5.0402076843,1.1323256123
H,4.351394956,-5.3170771977,1.6363098056
H,5.9044030085,-5.9368325688,1.0513249661
H,5.8030059185,-4.3205679549,1.7702547945
C,1.2347477625,-4.4711526046,-1.6755134475
H,2.0202723617,-4.6005511693,-2.4255991843
H,0.5869148715,-5.3531462824,-1.7067889395
H,0.6415813715,-3.6003293395,-1.9675296811
C,-1.3815155565,-4.2130399534,2.1266288285
H,-2.187697881,-4.2361984676,2.8655498994
H,-0.7426936754,-5.0862918651,2.2938621402
H,-0.7881701435,-3.3142159755,2.3145855368
C,-5.2880071288,-5.1391160599,-0.813116935
H,-4.3247292126,-5.469227366,-1.2134135363
H,-5.9174259417,-6.0229835462,-0.6705952259
H,-5.759607133,-4.5028997258,-1.5674043353
C,6.002967056,-1.4538506069,-0.1503369977
C,7.1608390785,-1.0717273183,-0.1570059058
C,-6.0851052098,-1.4206580576,0.0181110403
C,-7.2395880439,-1.0450922195,-0.0896343855
C,-8.5846360061,-0.6143935246,-0.2180370223
C,-9.6443083265,-1.5338566027,-0.1028379821
C,-8.8839205731,0.7390732863,-0.4629086991
C,-10.9582714684,-1.1075275288,-0.229366366
H,-9.4207708355,-2.5783705778,0.0860213165
C,-10.2017618158,1.1538888208,-0.5876034575
H,-8.0719599577,1.4525227581,-0.554866985
C,-11.2447992123,0.2355761964,-0.4717594426
H,-11.7658740728,-1.8271326429,-0.1383992136
H,-10.4184924771,2.2005330052,-0.7772369125
H,-12.274304695,0.5644059051,-0.5700871728
C,8.5061104356,-0.626937911,-0.1622322772
C,9.5661738904,-1.5516899386,-0.2226673877
C,8.8046468373,0.7480386527,-0.1077076442
C,10.8806790675,-1.109380229,-0.2275160812
H,9.3421880558,-2.6122030786,-0.2652951823
C,10.1232047273,1.1781054588,-0.1130880858
H,7.9919608481,1.4649236049,-0.0608335762
C,11.1666422665,0.2546346783,-0.1729285371
H,11.6891284587,-1.8322295208,-0.2742971853
H,10.3402414037,2.2407959858,-0.070398324
H,12.1967947351,0.5959657183,-0.1770570671
C,4.677787574,-1.8884281064,-0.1453829182
C,-4.762992704,-1.8530556972,0.1427009751

2b-1(T_1)
(CAM-B3LYP/6-31+G(d,p) for C, H, N, and O and CAM-B3LYP/LanL2DZ for Pt)
-2210.218361 hartree
C,0.8308433658,4.5854404813,-0.0018796603
C,1.6371797891,5.7254599629,-0.0724777996
H,1.2007306513,6.7190578891,-0.0288286792
C,3.0119496642,5.5911989473,-0.2010092549
H,3.643457549,6.4718747525,-0.2568995628
C,3.5736539576,4.3140644756,-0.2596754784
H,4.6498615879,4.2059844628,-0.3632234984
C,2.7693036705,3.1814892343,-0.1892362951
H,3.2194749996,2.196593089,-0.242816231
C,-1.3045208087,-0.9067034185,0.1349720211
C,-0.0630348761,-1.5446417864,0.0594169653
C,1.2051484789,-0.9326715517,-0.0488988851
C,1.3827134361,3.2865169061,-0.0577282442
C,-2.5226251912,3.2562458093,0.3264394152
C,-0.6244516551,4.6189773451,0.1367962432
C,-3.3612950049,4.3507212124,0.4311975954
C,-1.4279429634,5.7562086172,0.2345145112
C,-2.7987964779,5.6244664997,0.3803191518
H,-0.9702445835,6.7370642586,0.199517967
H,-3.4258185368,6.5067840763,0.458479436
N,-1.1973196574,3.3874204167,0.1775527672
Pt,0.0627781905,1.8214008954,0.0375621277
H,-2.879220861,2.234295857,0.3659948309
H,-4.4277403163,4.2038268369,0.5533379829
H,-0.0798277759,-2.6201143406,0.064295353
O,-1.4913526193,0.3707447498,0.108269496
O,1.4495011454,0.3562378348,-0.0693339644
C,2.4016927171,-1.7057171216,-0.1368662908
C,2.7494561586,-3.1435199564,-0.1788969333
N,3.5859829784,-1.0180933777,-0.1801741572
C,4.1055457969,-3.2426243902,-0.2086494319
H,3.6090857274,-0.0135870092,-0.0984714836
C,-2.5491603799,-1.6855412013,0.2149612202
C,-2.8996702761,-3.0146849067,0.4864846879
N,-3.7091790108,-0.996460962,0.000921694
C,-4.3146405052,-3.0952497109,0.4243844894
H,-3.7167669036,-0.0121539451,-0.2152062251
C,-5.1711949602,-4.3113827978,0.6202213639
H,-4.7722074489,-4.9181089854,1.4408547236
H,-6.169311354,-3.9914376634,0.9365536832
C,-2.0102143599,-4.1675599886,0.86043468
H,-1.2028402449,-4.2827652964,0.1303384624
H,-2.5930891404,-5.0903814365,0.7867818967
C,1.7997725533,-4.3028483159,-0.2314671078
H,1.027078269,-4.191819312,0.5346375337
H,2.3481117509,-5.2089354306,0.0403942737
C,4.960508183,-4.4719199783,-0.2325393516
H,4.4055765163,-5.3006624217,-0.6807082887
H,5.8211324917,-4.2869512599,-0.8843728995
C,5.4600839794,-4.8783891821,1.1611411101
H,4.6220124761,-5.1068422067,1.8259071995
H,6.09749657,-5.7652616836,1.0988821621
H,6.0414868841,-4.0740641692,1.6199021436
C,1.1586815339,-4.504328814,-1.611933636

H,1.927278846,-4.6918449733,-2.3672377504
H,0.4782697468,-5.3610691854,-1.6005888279
H,0.5916847763,-3.6248175576,-1.9275509771
C,-1.4281856445,-4.0639143679,2.2760711331
H,-2.2306028525,-4.0140074014,3.0177911256
H,-0.8053976225,-4.9338824514,2.5092696457
H,-0.8171970199,-3.1644970546,2.3915539324
C,-5.2971737452,-5.1762564995,-0.6407981767
H,-4.3183393534,-5.5306650932,-0.978068289
H,-5.9290334498,-6.0507546622,-0.4554401941
H,-5.7405479355,-4.6023935418,-1.459563089
C,5.9745232832,-1.5153453496,-0.2170926629
C,7.1603956397,-1.1676653238,-0.2361988067
C,-6.1152828682,-1.3685010567,-0.0719541121
C,-7.2537708587,-0.9836486709,-0.2305710587
C,-8.5969237516,-0.5355566319,-0.4185403868
C,-9.676821641,-1.3853435598,-0.1347658459
C,-8.8578423787,0.7598366303,-0.8903666185
C,-10.9818643841,-0.9472116116,-0.3199135874
H,-9.4805143528,-2.3875100925,0.2309776184
C,-10.1658556411,1.1908368273,-1.0714250743
H,-8.026466265,1.419477422,-1.1150880617
C,-11.2325027688,0.3408508369,-0.7877172971
H,-11.8080245362,-1.6150937168,-0.0969612662
H,-10.3536063241,2.1952588092,-1.4378617741
H,-12.2533847171,0.6801402318,-0.9309352954
C,8.5081775649,-0.7737880708,-0.2579557334
C,9.5409441193,-1.7378849298,-0.2790175522
C,8.8572974604,0.5954235932,-0.2603139129
C,10.867920028,-1.3403905727,-0.3012581978
H,9.2810337469,-2.7909652768,-0.2775534362
C,10.1888670356,0.9769514699,-0.2823911189
H,8.0700082232,1.3412486229,-0.2444117487
C,11.2007887665,0.0153711947,-0.3030432589
H,11.6516121278,-2.0912084414,-0.3173453495
H,10.4433402109,2.0320933158,-0.2837509345
H,12.2418769077,0.3205619221,-0.3204212637
C,4.6725134412,-1.8958277311,-0.1953978195
C,-4.7908779742,-1.8232830413,0.1146121937

2b-4·Cl(S_0)

(CAM-B3LYP/6-31+G(d,p) for C, H, N, and O and CAM-B3LYP/LanL2DZ for Pt)

-2670.6254997 hartree
C,5.1744978043,0.7114533227,1.9862879873
C,6.2272609596,1.5190287548,2.4297874889
H,7.1060132252,1.0803370067,2.8943902708
C,6.1524946,2.8952866651,2.2773105678
H,6.965213238,3.5286980642,2.6191319413
C,5.0202940953,3.4552541635,1.679643178
H,4.9550927379,4.5332601305,1.5571491094
C,3.9760159996,2.6500380955,1.2391390388
H,3.1076037871,3.0981479832,0.7762927938
C,0.2414304427,-1.4970947657,-0.302944496
C,-0.307252874,-0.2592563961,-0.6715981672
C,0.2160112071,1.0210441763,-0.4460433553
C,4.0217651246,1.2598772511,1.3790458208
C,3.8653784036,-2.6475066626,1.6645127355
C,5.1590496237,-0.7439429789,2.1087678103
C,4.8190335295,-3.4849758716,2.2160339847
C,6.1526873768,-1.5459894292,2.6733290449
C,5.9848891921,-2.9198550627,2.7269876927
H,7.0491447578,-1.0843773221,3.0687289308
H,6.754970751,-3.5464091051,3.166256007
N,4.0342955871,-1.3198476761,1.6100294092
Pt,2.6639695738,-0.0679230795,0.8217808907
H,2.932209233,-3.0057717674,1.2507818311
H,4.6428854591,-4.5536343904,2.2425386898
H,-1.2455491564,-0.2979206142,-1.2023777421
O,1.3654671581,-1.6608027066,0.2978934515
O,1.3568640149,1.3051377755,0.0931724058
C,-0.5813874787,2.1804173516,-0.8662274883
C,-0.2295408698,3.5325931262,-1.0003734471
N,-1.8982218832,2.057181504,-1.2291613864
C,-1.3739757167,4.2198337504,-1.4616447753
H,-2.4951245171,1.2132476121,-1.217658852
C,-0.5055112024,-2.7201744161,-0.6318660996
C,-0.0813367901,-4.0557970553,-0.6829162406
N,-1.8209206323,-2.6885123459,-1.0133234143
C,-1.1833413699,-4.831374518,-1.1078649338
C,-1.2206648481,-6.3076182485,-1.372688094
H,-0.5532564157,-6.8279238664,-0.6761896826
H,-2.2301422293,-6.6813089496,-1.1680240796
C,1.2669070175,-4.6205071207,-0.3429390669
H,2.0352558472,-3.8658146741,-0.5229394881
H,1.4845156532,-5.4592602015,-1.0148851577
C,1.1044331255,4.1757965757,-0.7577993608
H,1.4628244173,3.892877576,0.2339207551
H,0.9675120343,5.2624258893,-0.7395194036
C,-1.5192429082,5.6864107034,-1.7421466877
H,-0.6025626277,6.0697709159,-2.2054169278
H,-2.3169350448,5.8266734608,-2.4797169318
C,-1.8426793635,6.5168846076,-0.4929264603
H,-1.056987866,6.4141747624,0.2620630379
H,-1.942761968,7.5800863905,-0.7391628228
H,-2.7803756444,6.180474467,-0.0410859676
C,2.1615501534,3.8232676868,-1.8103162837
H,1.8190345926,4.1050219761,-2.8113083516
H,3.1041050095,4.345670884,-1.6123336419
H,2.3639910042,2.7499457871,-1.8071121578
C,1.3452866652,-5.1152378892,1.1079189012
H,0.5973096719,-5.8915222649,1.2957292711
H,2.3311465593,-5.5391295134,1.3345118657
H,1.1466996517,-4.2969110547,1.8057883929
C,-0.8357743552,-6.674887002,-2.8124667507
H,0.1754268755,-6.3292640211,-3.049288298
H,-0.8714054339,-7.7591274855,-2.9679083962
H,-1.5202989681,-6.2044832431,-3.5240411537
C,-3.7376809941,3.422402795,-1.9902913733
C,-4.9009070736,3.4568923706,-2.3227719118
C,-3.5800650793,-4.1936865264,-1.6993783907
C,-4.737707414,-4.3070546403,-2.0335513539
C,-6.1121052789,-4.2721712546,-2.4158360278
C,-6.8267144737,-5.4348568349,-2.7355262447
C,-6.7605688684,-3.0265737719,-2.4704736295
C,-8.1650979664,-5.3552126958,-3.1035142833
H,-6.3240193772,-6.3957233072,-2.6924322822
C,-8.097856343,-2.9602444504,-2.8396730933
H,-6.1951030292,-2.1323765278,-2.2199889159
C,-8.8056914878,-4.1191815777,-3.1569378334

H,-8.7099673088,-6.2621319133,-3.349461112
H,-8.5919414012,-1.9940891051,-2.8797958126
H,-9.8511432454,-4.0588741718,-3.4447472238
C,-6.2746415699,3.3338002151,-2.6882415803
C,-7.0400325084,4.4368807049,-3.0911910515
C,-6.8701599536,2.0617249244,-2.6398750882
C,-8.3761297444,4.2728582712,-3.4389388743
H,-6.5781685378,5.4182639068,-3.1282149534
C,-8.205752334,1.9109456019,-2.9898712014
H,-6.2657023106,1.2143867008,-2.3257389413
C,-8.964256972,3.0108371442,-3.3896347313
H,-8.9603767154,5.1342443032,-3.7496996835
H,-8.6588266575,0.9248590382,-2.9494331817
H,-10.0080598381,2.8846064003,-3.6616853366
C,-2.3912307782,3.273680182,-1.5851943068
C,-2.2467139266,-3.9498907784,-1.2964174572
H,-2.4572869996,-1.873726261,-1.0671908764
Cl,-3.9539694996,-0.3731316796,-1.40251107

2c-1(S0)

(CAM-B3LYP/6-31+G(d,p) for C, H, N, O, and F and CAM-B3LYP/LanL2DZ for Pt)

-2408.7470842 hartree
C,0.8620677614,4.5209080449,0.3327384171
C,1.6843312834,5.6513967583,0.3286729773
H,1.2581133802,6.6495102119,0 .3672411475
C,3.0625423535,5.5012526524,0.2772829787
H,3.7066798305,6. 3743731881,0.2744661509
C,3.6117783822,4.2186171374,0.2329320133
H,4.6 90875371,4.097996604,0.1984323434
C,2.7913293916,3.0950005709,0.234624 3163
H,3.2350798901,2.1062598693,0.2051819016
C,-1.3175724776,-0.95070 90387,0.2741198426
C,-0.0733733848,-1.600017515,0.2387917191
C,1.18642 41341,-0.9917145557,0.1668344417
C,1.4014902847,3.217232592,0.28092322 02
C,-2.5161384036,3.2243675556,0.4937575637
C,-0.5972251993,4.5691087 655,0.4046406674
C,-3.3468604248,4.3260792055,0.5807579525
C,-1.392440 4667,5.7133722614,0.4857539613
C,-2.7692855856,5.5941760195,0.57213286 2
H,-0.9236712683,6.6895441876,0.484997671
H,-3.389916426,6.4819733209 ,0.637398772
N,-1.1844147192,3.3438174676,0.4016716653
Pt,0.0608895961 ,1.7670720938,0.2737956152
H,-2.8863497052,2.2065507848,0.5026204033
H ,-4.4188512832,4.1893424909,0.6570372705
H,-0.092844368,-2.6745370275, 0.2087854126
O,-1.4990044268,0.3176487338,0.2522839441
O,1.4263467412, 0.2746145669,0.1628972998
C,2.4133720358,-1.7925675044,0.0669324614
C, 2.7399755362,-3.1544903312,0.088594411
N,3.5812824995,-1.0962708694,-0 .0858944084
C,4.1457691954,-3.2485921404,-0.0637641272
H,3.6051510621, -0.0889255585,-0.1314917975
C,-2.567069574,-1.7272783579,0.2965391309
C,-2.9293176487,-3.0515003442,0.5676440201
N,-3.7153551547,-1.03877913 42,0.0166509906
C,-4.3399586317,-3.1324441618,0.4390397432
H,-3.716172 1808,-0.0575498099,-0.2132113931
C,-5.2060451826,-4.3456797001,0.60961 00184
H,-4.8462869786,-4.9414137077,1.4557472931
H,-6.2176052258,-4.02 16365401,0.8750018933
C,-2.0613087847,-4.198989101,1.0050629156
H,-1.2 130806787,-4.3216391297,0.3257757143
H,-2.6391661457,-5.1227318742,0.9 094976618
C,1.8379080247,-4.3507926434,0.2069662539
H,1.0392313327,-4. 1581572938,0.9282204509
H,2.4152024429,-5.1782248349,0.6307489911
C,4. 9817255031,-4.494574862,-0.0704851373
H,4.4341485327,-5.3044384892,-0. 5644200023
H,5.8753685976,-4.3175402735,-0.6780752218
C,5.4089213487,- 4.9464497764,1.3323457273
H,4.5396556896,-5.1506101039,1.9648275102
H, 6.0141437585,-5.8571947504,1.2836561365
H,6.0004086137,-4.1696442108,1 .8252565461
C,1.2469190801,-4.7996449956,-1.1365952449
H,2.0439039181, -5.0446527568,-1.8446464767
H,0.6193746364,-5.6879421641,-1.0132334156
H,0.6391354501,-4.0114128444,-1.5901575331
C,-1.5632712313,-4.0739377 925,2.4505769803
H,-2.4069210455,-4.0079697872,3.1437898713
H,-0.95977 92968,-4.9424574427,2.7326648129
H,-0.9529474984,-3.1768978014,2.58541 16
C,-5.272874583,-5.2261779138,-0.6450346106
H,-4.2796314773,-5.58529 47901,-0.9309180556
H,-5.9133606821,-6.0979240857,-0.4782882755
H,-5.6 767882574,-4.6631698336,-1.4913050891
C,5.9683378814,-1.4966395211,-0. 3380957997
C,7.1088772947,-1.1164627464,-0.4854256241
C,-6.1126884082, -1.4130336143,-0.1734416033
C,-7.2414263239,-1.033065124,-0.3951314867
C,-8.5741474157,-0.5901057474,-0.6574373373
C,-9.6730930516,-1.373677 8621,-0.2737178242
C,-8.8058726396,0.6337800711,-1.3031994783
C,-10.97 06296156,-0.9493438905,-0.5273473226
H,-9.5021029795,-2.3201971743,0.2 269027555
C,-10.0997606717,1.0681472565,-1.5587937715
H,-7.9617518427, 1.2425964565,-1.6077220795
C,-11.1570202592,0.265560602,-1.1648492676
H,-11.8300458588,-1.5432037244,-0.2378315728
H,-10.2957064595,2.010518 3588,-2.0574386558
C,8.4540435202,-0.6684012878,-0.6584518553
C,9.5097 100993,-1.5906006861,-0.7206795379
C,8.7408588812,0.7006364908,-0.7686 012016
C,10.8191095449,-1.1601108876,-0.8884091239
H,9.2957338112,-2.6 502794505,-0.6364271838
C,10.0469692663,1.1408729361,-0.9365253543
H,7 .9300876174,1.4191568497,-0.7225498279
C,11.0605130322,0.1992696943,-0 .9929096458
H,11.6458188343,-1.8594860358,-0.9385100897
H,10.285302329 6,2.1947646276,-1.0235949657
F,-12.4183669475,0.6838987214,-1.41228444 26
F,12.333911951,0.6231708977,-1.1564230017
C,-4.7981537299,-1.864587 8655,0.0877708939
C,4.6392794787,-1.9495843802,-0.1701880715

2c-2(S0)

(CAM-B3LYP/6-31+G(d,p) for C, H, N, O, and F and CAM-B3LYP/LanL2DZ for Pt)

-2408.7448491 hartree
C,2.4172833092,-1.3285712556,-4.1682462708
C,2.2646512204,-1.3314316704,-5.557651493
H,2.9029269395,-1.9454558964,-6.1861352316
C,1.2840674063,-0.5449645951,-6.1451696335
H,1.1594916903,-0.543573416,-7.2230470313
C,0.4562047389,0.2373321997,-5.3380218531
H,-0.3191875075,0.8457924454,-5.7950241523
C,0.6114263236,0.2412749676,-3.9552031227
H,-0.0482626256,0.8438674641,-3.3406070914
C,1.9137882863,-0.3814237507,1.6153877694
C,0.8953975951,0.5884186517,1.5001294516
C,0.3008699841,1.0563657488,0.3245265108
C,1.5959862236,-0.5339246896,-3.3398849947
C,4.1842008162,-2.6653942926,-1.3015146739
C,3.401734338,-2.1357017513,-3.4486046968
C,5.1227623403,-3.5465133345,-1.8069563715
C,4.3328257506,-3.011642812,-4.0077696696
C,5.1961851504,-3.7188040572,-3.1873139845
H,4.3723452744,-3.1329048563,-5.0831535552
H,5.921285462,-4.401085972,-3.6190928102
N,3.3525975798,-1.9816448705,-2.0993894108
Pt,1.9841769126,-0.6769920801,-1.4096259953
H,4.0642603505,-2.4762124526,-0.2420882046
H,5.7778090301,-4.0822964249,-1.1305578101
H,0.5977122317,1.08797263,2.4068780285
O,2.4991678412,-0.9661209684,0.6464255145
O,0.5753932447,0.6802946754,-0.8760124615
C,-0.7290608212,2.1014213063,0.3708877955
C,-1.517759874,2.6881220593,1.3667659263
N,-1.0752095914,2.6782177954,-0.8196259785
C,-2.3454329887,3.6476377574,0.7309465656
H,-0.648578432,2.4134578376,-1.6947766115
C,2.3620119562,-0.7482566217,2.965983483
C,3.5560225585,-1.3101202183,3.4169313564
N,1.5717294328,-0.5212982453,4.066733195
C,3.4664496327,-1.4186712877,4.82893976
H,0.6108622294,-0.2200522841,4.0406195151
C,4.4938083769,-1.9735254912,5.770689115
H,5.4933042554,-1.6542310467,5.4556449324
H,4.3342572435,-1.5397497951,6.7631317969
C,4.7598520889,-1.6839817308,2.5993074093
H,4.4549589071,-2.3657418619,1.8013438516
H,5.4505194757,-2.2456268251,3.2356567787
C,-1.5440021114,2.4099129054,2.8439604648
H,-1.3677839318,1.345258601,3.0275138654
H,-2.5548587397,2.6050946898,3.2158025791
C,-3.3946807209,4.5112982068,1.3669967344
H,-3.0809101802,4.7885840051,2.3789936269
H,-3.4714255348,5.448153401,0.8051804136
C,-4.7757666793,3.8447926759,1.4239307576
H,-4.74227303,2.911412387,1.9941107263
H,-5.5103690146,4.5044813873,1.8964455727
H,-5.1306774862,3.6040842899,0.4178189195
C,-0.5510920742,3.2595837122,3.6493963027
H,-0.7589873905,4.3250963631,3.5162498902
H,-0.616581871,3.0338975159,4.718518847
H,0.4802995876,3.0895745549,3.327424661
C,5.4969065933,-0.4835062492,1.9949376754
H,5.823190332,0.2076262354,2.7779565627
H,6.383648112,-0.8098347468,1.4419187814
H,4.8510262864,0.064093184,1.3048082124
C,4.4554682172,-3.503581725,5.8766355447
H,4.630001806,-3.9723237802,4.9035151095
H,5.2201621168,-3.8673317501,6.5701138308
H,3.4796949281,-3.8423725286,6.2364882627
C,-2.5774307102,4.3835730289,-1.6919191966
C,-3.0365547808,5.0439760486,-2.5975085649
C,1.6154564776,-0.8505505559,6.4779816631
C,1.1055199862,-0.7796805106,7.5745751936
C,0.509155331,-0.6994566808,8.8701281812
C,1.233036903,-1.0783879145,10.0108723585
C,-0.8081570764,-0.2413447868,9.0228870275
C,0.6589456697,-1.003287528,11.2728408101
H,2.251834265,-1.4329462619,9.9008024854
C,-1.3908619452,-0.1625016299,10.2807374194
H,-1.3746950795,0.052378444,8.1461837232
C,-0.643435428,-0.5458415982,11.3814312926
H,1.2044907078,-1.2920947232,12.1639312549
H,-2.4074276964,0.1888095626,10.4157803992
C,-3.5772449545,5.8226338418,-3.6660208333
C,-4.6209578078,6.7299355589,-3.4291459831
C,-3.0731334871,5.6921303446,-4.9689271216
C,-5.1502223062,7.4887415984,-4.46456568
H,-5.0155584809,6.8362318019,-2.4247524398
C,-3.5962118875,6.4461208819,-6.0109716739
H,-2.2651622907,4.9944433995,-5.1592442467
C,-4.6257405371,7.3302360563,-5.7361535885
H,-5.9564644923,8.1940424747,-4.2984287924
H,-3.2179143819,6.3574421182,-7.0229456049
C,-2.0431212089,3.619141657,-0.628937637
C,2.2156771942,-0.9360222878,5.2006282321
F,-5.1381151009,8.0667472298,-6.7475261445
F,-1.2063788439,-0.4706969386,12.6079766784

2c-3(S_0)
(CAM-B3LYP/6-31+G(d,p) for C, H, N, O, and F and CAM-B3LYP/LanL2DZ for Pt)
-2408.743556 hartree
C,1.4116432938,0.6591344755,-4.7832854318
C,1.3740394405,0.4765563039,-6.1690330177
H,2.083047659,0.9839285836,-6.8166133078
C,0.4214536636,-0.3632598671,-6.7272260405
H,0.38667124,-0.5103262401,-7.8017502845
C,-0.4886644598,-1.0176831306,-5.8947410763
H,-1.2338352627,-1.6785700984,-6.3289160646
C,-0.4505705066,-0.8351833155,-4.5158116914
H,-1.1577311887,-1.3532224807,-3.881596792
C,0.4478207359,0.5443973021,1.0161345249
C,-0.6666321508,-0.3023603906,0.9502103922
C,-1.2007887218,-0.9331793834,-0.1882321932
C,0.4967499415,0.0052800513,-3.9302090937
C,3.0125071875,2.2983821472,-1.9794604918
C,2.3700726583,1.5210261998,-4.0961121002
C,4.0146972326,3.0853489165,-2.5156370842
C,3.3701827313,2.2958334218,-4.6865078739
C,4.1955471519,3.078538885,-3.8973804431
H,3.4898960682,2.2808860478,-5.762639844
H,4.9724855788,3.6832753943,-4.3538216768
N,2.2197838986,1.5362453006,-2.7457262516

Pt,0.7392070104,0.3870104865,-2.0079315379
H,2.8032316134,2.2548756685,-0.9179611415
H,4.6333802011,3.6901608781,-1.8636279082
H,-1.1791160109,-0.4801801777,1.8799459556
O,1.1757970078,0.8900704653,0.0215967083
O,-0.810423715,-0.7954831836,-1.4013989529
C,-2.335899243,-1.8441770949,0.0164624981
C,-3.3699198097,-2.2371974992,-0.8311173994
N,-2.5757089313,-2.4072463478,1.2485156448
C,-4.2485866711,-3.0582560441,-0.0763584422
H,-1.937536755,-2.3968738147,2.0278923447
C,0.8877006328,1.0939237492,2.3077953564
C,0.3404910276,1.2059291409,3.5902464419
N,2.1208799977,1.6840792032,2.3363962156
C,1.2955523212,1.8792922293,4.3944586084
H,2.7204805025,1.7278694226,1.5274982812
C,1.1558481992,2.2766746673,5.8345829122
H,0.5735554949,1.5202309402,6.3713648288
H,2.1475672782,2.2834197766,6.2989803045
C,-0.9915258557,0.7268045874,4.0973121956
H,-1.7495677932,0.8088902916,3.3135242002
H,-1.3239255018,1.397899003,4.8955500134
C,-3.5560588142,-1.8918241561,-2.2796718534
H,-2.9993428122,-0.9825700191,-2.5140314522
H,-4.6143546789,-1.6755191278,-2.4650333062
C,-5.5487056442,-3.6566369612,-0.5258193574
H,-5.4896244877,-3.9087200487,-1.5889848702
H,-5.7113308198,-4.6004348649,0.0056028504
C,-6.7497024725,-2.7309304848,-0.2889581799
H,-6.6277346996,-1.782763446,-0.8210302831
H,-7.6774377902,-3.1970702201,-0.635450533
H,-6.8595388876,-2.5026198719,0.7750966812
C,-3.1037245292,-3.0260575151,-3.2096412828
H,-3.666468902,-3.9447725322,-3.020085295
H,-3.2509229007,-2.7530344464,-4.2594138762
H,-2.044898871,-3.2512565498,-3.0575050142
C,-0.9529665611,-0.7059065217,4.6471786559
H,-0.2682792755,-0.7783046938,5.4971360461
H,-1.9447482809,-1.0261213401,4.9812982578
H,-0.5944173389,-1.4132807606,3.8924885562
C,0.5025574281,3.652462882,6.0225320795
H,-0.500005318,3.6782936117,5.5850471359
H,0.415454333,3.9032085124,7.0843797756
H,1.0953328582,4.4319079969,5.5353238248
C,-4.1803328495,-3.8869960614,2.3257535296
C,-4.578057669,-4.5154632478,3.2819997021
C,3.6143158014,2.8008503345,3.9025984724
C,4.6579382048,3.3476739,4.1843315618
C,5.8864603736,3.9946416565,4.5207471122
C,6.0281972934,4.6582592476,5.7489338488
C,6.970524946,3.976155069,3.6302459581
C,7.2198733636,5.2882590714,6.0819489849
H,5.1947667342,4.6770915883,6.4423492037
C,8.1656715857,4.6049308716,3.9532537996
H,6.8705755257,3.4611698364,2.6811960003
C,8.2653496894,5.248899431,5.1749431393
H,7.3454830637,5.8048334254,7.0266425984
H,9.0127401962,4.5992796588,3.2768461446
C,-5.0516091647,-5.2570534208,4.4072555588
C,-6.2444928894,-5.9913037232,4.324479367
C,-4.3324139846,-5.2635715488,5.6119767422
C,-6.7102029077,-6.7136235504,5.4150008046
H,-6.8053648793,-5.9916582686,3.3964721325
C,-4.7899840535,-5.9829338505,6.7079644232
H,-3.408586323,-4.7002543903,5.6833917823
C,-5.9717382783,-6.6940590617,6.5861088015
H,-7.6296393713,-7.285852459,5.3674026513
H,-4.2459817098,-5.9987621261,7.6454533877
C,-3.71783018,-3.1606470458,1.2042158978
C,2.3948817508,2.1583641232,3.5862350277
F,-6.4211669698,-7.3958832279,7.6504837341
F,9.4270710546,5.8616587552,5.4940557479

2c-4(S_0)
(CAM-B3LYP/6-31+G(d,p) for C, H, N, O, and F and CAM-B3LYP/LanL2DZ for Pt)
-2408.7393894 hartree
C,0.5405374284,-4.1783079106,-2.6997768565
C,0.2849500581,-5.0572546519,-3.7564122316
H,0.8381846868,-5.9868193994,-3.8529703557
C,-0.6880810617,-4.7427379625,-4.6940675225
H,-0.8919354968,-5.4204820897,-5.5166160373
C,-1.4030903055,-3.5503562025,-4.5673697097
H,-2.1693312265,-3.3038291538,-5.2972297924
C,-1.146634367,-2.6752705118,-3.5161151004
H,-1.7112275923,-1.7565110178,-3.4272101488
C,0.6299241412,0.2976030717,1.097267606
C,-0.3675454508,1.0527524745,0.4510390038
C,-1.0868155252,0.710433249,-0.7103746714
C,-0.1700292695,-2.9663049125,-2.5632484761
C,2.4407216948,-3.5193381956,0.3126220682
C,1.5297532212,-4.4217636281,-1.6520987959
C,3.2801174692,-4.6053860681,0.4809675669
C,2.3592292097,-5.5372641289,-1.5281990744
C,3.2375454846,-5.6304724659,-0.4615537546
H,2.3066616275,-6.325518065,-2.2688491478
H,3.8832228102,-6.4970367841,-0.3619022291
N,1.596962525,-3.430716541,-0.725131425
Pt,0.3607399691,-1.8683289598,-1.0093341636
H,2.4113909862,-2.6845617352,1.0020429752
H,3.948593038,-4.6423991131,1.3326977087
H,-0.5008840877,2.0620215221,0.81617745
O,1.0771790885,-0.830287182,0.712970571
O,-0.959578512,-0.3599644547,-1.4024112234
C,-2.0761471997,1.6759851528,-1.2090217578
C,-2.5926123354,1.8938420334,-2.4849892431
N,-2.5938992184,2.6480490934,-0.3842433329
C,-3.44235726,3.0293569649,-2.4113863458
H,-2.4996928456,2.6708817982,0.6174849945
C,1.2351066838,0.855919875,2.3169702152
C,2.5129874981,0.7155395142,2.8512364041
N,0.5351933608,1.7112658087,3.1311385315
C,2.5692784492,1.5121069166,4.0258627172
H,-0.4518764996,1.8982597856,3.0594884325
C,3.7316491339,1.681564506,4.9587615858
H,4.6594749294,1.757977657,4.3811805572
H,3.6236071348,2.6328365584,5.4897063213
C,3.6645644483,-0.0532607294,2.2666020613
H,3.3576901835,-1.0850914468,2.0802561585
H,4.4668155033,-0.098772262,3.0094415433

C,-2.3367613417,1.0998671698,-3.7325415099
H,-1.4006049091,0.5481345555,-3.6306578126
H,-2.2134341182,1.7872618758,-4.5771356099
C,-4.1610722917,3.7048508919,-3.5417164084
H,-4.4607110021,2.9596535485,-4.2848093782
H,-5.0866467332,4.1508516893,-3.1622634466
C,-3.3199699081,4.792966913,-4.2228544029
H,-2.3926864685,4.37817421,-4.6291478862
H,-3.8729543995,5.2568210642,-5.0456230772
H,-3.0490106813,5.5768301551,-3.5098425318
C,-3.4789490061,0.1223256818,-4.0422433031
H,-4.4274285908,0.6491125681,-4.1820445138
H,-3.2714783307,-0.44369825,-4.9558218172
H,-3.6143219045,-0.5866835711,-3.2212515038
C,4.2122732643,0.5528099763,0.9692009131
H,4.537891405,1.5854489744,1.1272062467
H,5.0694712588,-0.0212041389,0.6034783906
H,3.4499959513,0.5535735063,0.1862566098
C,3.8580307514,0.5445953269,5.9810273666
H,3.9856089062,-0.4218883568,5.4842407678
H,4.7180421294,0.7035850117,6.6390682762
H,2.9601428663,0.4813015282,6.6024517755
C,-4.134034579,4.5305272752,-0.4796666657
C,-4.7338696372,5.4464293918,0.0388536426
C,0.8341464149,2.9512321933,5.2053420598
C,0.4203830546,3.6783140274,6.0816877557
C,-0.0607654282,4.5353405108,7.1185647383
C,0.8312803373,5.1132914846,8.0345903463
C,-1.4309743587,4.8123171537,7.2384874197
C,0.3701021943,5.9460147093,9.0454129207
H,1.8918562564,4.9050346972,7.9476525325
C,-1.9016796001,5.6453649058,8.2447800836
H,-2.1276111029,4.3662743457,6.5372771076
C,-0.9892582928,6.1957443899,9.1289488649
H,1.0460912192,6.3999690842,9.7609793238
H,-2.9571263929,5.8682829291,8.3517993416
C,-5.4426053834,6.5295470153,0.6439077174
C,-6.5488200197,7.1035849393,-0.0006981903
C,-5.0434677628,7.0371397107,1.8894818161
C,-7.2397965218,8.1585288584,0.5802278094
H,-6.8640679531,6.7151587968,-0.962722715
C,-5.7299503224,8.0897059752,2.4801254988
H,-4.1862672206,6.602320125,2.3915204473
C,-6.8156017823,8.6294444273,1.8113184513
H,-8.0957457167,8.6136556856,0.095187594
H,-5.4339408443,8.4943239783,3.4412384878
C,-3.437075553,3.4626298158,-1.0911433928
C,1.3199602093,2.1019140376,4.1846439595
F,-7.4861619455,9.6549466114,2.3816207097
F,-1.4427247358,7.006683746,10.1104731544

2c-1(S_1)

(CAM-B3LYP/6-31+G(d,p) for C, H, N, O, and F and CAM-B3LYP/LanL2DZ for Pt)

-2408.7426096 hartree
C,0.8504662308,4.5883766644,-0.0275909575
C,1.6608614142,5.7254368186,-0.0966644899
H,1.2276783269,6.7206572095,-0.0573168687
C,3.0359086063,5.5861595902,-0.2184729667
H,3.6706887892,6.4645253462,-0.2732804214
C,3.5933882723,4.3070200782,-0.2723707532
H,4.6696525277,4.1952463879,-0.3716466793
C,2.7849003675,3.1771855732,-0.2030436108
H,3.2319327429,2.19058072,-0.2537025999
C,-1.3029398643,-0.9066540676,0.1417952443
C,-0.0404323046,-1.5426274056,0.080934193
C,1.225223539,-0.9311572532,-0.0384200614
C,1.3979494685,3.2870204031,-0.0778417659
C,-2.508924558,3.2685362357,0.275781059
C,-0.6055822766,4.6266060439,0.101081764
C,-3.3459201029,4.3655636746,0.3693927297
C,-1.407166719,5.7658529145,0.1883806859
C,-2.7798453758,5.6377601836,0.3216297641
H,-0.9469107961,6.7455620372,0.1546242121
H,-3.40532727,6.5218601566,0.3914671133
N,-1.1820748147,3.3963699598,0.1408880358
Pt,0.0726623892,1.8274761755,0.0175988728
H,-2.8660296813,2.2466052552,0.3119152088
H,-4.41411813,4.2223042108,0.4803058569
H,-0.0452909178,-2.6182278485,0.1166801291
O,-1.4890877796,0.3806692814,0.0909267622
O,1.4594579436,0.3557713687,-0.0817811031
C,2.4291962358,-1.7244647593,-0.0970886452
C,2.764199718,-3.1133222153,-0.1714056934
N,3.608721188,-1.0334554955,-0.0905775499
C,4.1594158735,-3.202183368,-0.2010449032
H,3.6250862006,-0.0248208879,-0.0478548811
C,-2.5144616781,-1.6886264607,0.2129256865
C,-2.8616875612,-3.0580991649,0.4195146875
N,-3.688755837,-1.0037539362,0.0601985213
C,-4.25887907,-3.1454481776,0.3724565363
H,-3.7002659288,-0.0109635692,-0.1151915846
C,-5.1096691685,-4.3729020078,0.5113900688
H,-4.6797905806,-5.0370111743,1.268718156
H,-6.0942707051,-4.0777906378,0.8881455947
C,-1.9564816042,-4.2164002343,0.7183261494
H,-1.1429348441,-4.2680632143,-0.012564472
H,-2.5239003331,-5.1411245864,0.5792366267
C,1.8441942609,-4.2933422869,-0.2735385695
H,1.0538627729,-4.2315543443,0.481540272
H,2.4108298919,-5.1944458016,-0.021525536
C,5.0029334505,-4.4411649517,-0.251285461
H,4.5191885364,-5.1918877247,-0.8849204794
H,5.9543514983,-4.1997642153,-0.736113063
C,5.2799055139,-5.0398627696,1.134339008
H,4.3492810671,-5.3154807819,1.6393139698
H,5.9014240415,-5.9370095187,1.0543032008
H,5.8013226399,-4.3196271931,1.7711174912
C,1.2282746108,-4.4726221154,-1.6688330682
H,2.0118703915,-4.603632915,-2.4206397273
H,0.5798944708,-5.3542888477,-1.6972459655
H,0.6349196093,-3.6018948188,-1.960743174
C,-1.3828862124,-4.1994920054,2.1425106464
H,-2.1888693245,-4.2180336896,2.8817652323
H,-0.7444953487,-5.072084419,2.3146220597
H,-0.7890292337,-3.2999234921,2.3251431859
C,-5.2825678933,-5.1407646418,-0.8058892315
H,-4.3172977139,-5.4720467254,-1.2002828101
H,-5.9121648084,-6.0244004152,-0.6628740198
H,-5.7506716245,-4.5078399209,-1.565140606

C,6.0014489737,-1.4550950395,-0.1560463754
C,7.159164938,-1.0734340086,-0.1646055902
C,-6.0854244447,-1.4191926921,0.0105804159
C,-7.2400254937,-1.0465519926,-0.1028520085
C,-8.5855332162,-0.6198260245,-0.237891652
C,-9.6444192619,-1.5401120748,-0.1179717294
C,-8.8885647329,0.7309676576,-0.4941631641
C,-10.9616187176,-1.1268152362,-0.249049646
H,-9.4216735835,-2.5827728897,0.0795416084
C,-10.2037372893,1.1504836284,-0.6263403368
H,-8.0801764784,1.4472325643,-0.5905635717
C,-11.2158996548,0.2121487035,-0.500741777
H,-11.7874037447,-1.8234415101,-0.1592093823
H,-10.4528869546,2.1867684041,-0.8246715549
C,8.5042924058,-0.6289335325,-0.1722266445
C,9.5651536807,-1.5532387734,-0.2329342396
C,8.8044794186,0.7461542721,-0.1201366979
C,10.8822332294,-1.1203027366,-0.2408996517
H,9.3436595265,-2.6139312825,-0.2738047251
C,10.1196505609,1.1847839622,-0.127587728
H,7.994089828,1.4650293717,-0.0730139965
C,11.1338575307,0.24184029,-0.1880356048
H,11.7101072617,-1.8186688425,-0.2873021997
H,10.3673626993,2.2394269129,-0.0875887739
C,4.6758519125,-1.8895994008,-0.1484964848
C,-4.7631729228,-1.8504269682,0.1414046726
F,-12.4985744468,0.6180550776,-0.6287521732
F,12.416477177,0.6663439797,-0.1957224047

2c-1(T$_1$)
(CAM-B3LYP/6-31+G(d,p) for C, H, N, O, and F and CAM-B3LYP/LanL2DZ for Pt)
-2408.6636565 hartree
C,0.7994657096,4.590364635,0.0889841206
C,1.5964318214,5.7376047463,0.0307263506
H,1.1529253732,6.7269074932,0.0952129174
C,2.9709502673,5.6162157418,-0.1125164992
H,3.5951812832,6.5025781487,-0.1590534288
C,3.5416790566,4.3447020703,-0.1984006894
H,4.6176526742,4.2469628871,-0.3140520074
C,2.7466644149,3.2048242235,-0.1400449328
H,3.203609264,2.2244580496,-0.215494686
C,-1.2920936554,-0.9203044994,0.1466911533
C,-0.046088478,-1.547051439,0.0547516252
C,1.2165559249,-0.9230491957,-0.0504072756
C,1.3606801503,3.2969491782,0.0061083775
C,-2.540853115,3.2298573153,0.4228938902
C,-0.6547858584,4.6102512068,0.2406596404
C,-3.3866810469,4.3158507553,0.5545651809
C,-1.4658585829,5.7394011796,0.3655695577
C,-2.8342925897,5.5946158742,0.5211796699
H,-1.0158959173,6.7242058276,0.344059111
H,-3.467172306,6.4705667071,0.6206543933
N,-1.2179630717,3.3738029262,0.2644335588
Pt,0.0528328458,1.8202340008,0.0873035616
H,-2.889408921,2.2046578851,0.4478415621
H,-4.450820076,4.1587959004,0.6839917673
H,-0.054343864,-2.6225778567,0.0421323105
O,-1.4894727795,0.3556386982,0.1429409019
O,1.4502254161,0.367835147,-0.0529108567
C,2.4188269654,-1.6847835474,-0.1549857742
C,2.7787364099,-3.1190743751,-0.2163013578
N,3.5973442728,-0.9862526478,-0.1927338813
C,4.1356063224,-3.206411653,-0.2463625308
H,3.6116841328,0.0162401184,-0.0869615853
C,-2.5302306273,-1.7106596251,0.217806347
C,-2.8691364155,-3.0462416295,0.4709586059
N,-3.6960714674,-1.0279437561,0.0155157953
C,-4.2836178685,-3.1376288288,0.410153846
H,-3.7114663666,-0.0404246714,-0.1852038881
C,-5.1295114394,-4.3637765607,0.5893234564
H,-4.7238549171,-4.9790952764,1.4001731856
H,-6.1297838409,-4.0573744467,0.9123053752
C,-1.9692379138,-4.1969053987,0.8263706117
H,-1.1638570886,-4.2959400426,0.0916852978
H,-2.5450101884,-5.1232911629,0.7425596677
C,1.8384419484,-4.2850720908,-0.2856225363
H,1.0669420129,-4.1927900882,0.4842008265
H,2.3946852209,-5.1910364035,-0.0300061822
C,5.0009130148,-4.4280740058,-0.2862101874
H,4.4530405203,-5.2554336012,-0.7453881797
H,5.8598783849,-4.2276032908,-0.9357101877
C,5.5035936868,-4.848877213,1.1020882792
H,4.6673301853,-5.0937811808,1.7632096363
H,6.1488047072,-5.7291796198,1.0282654602
H,6.0774212346,-4.0457285262,1.5723874468
C,1.1952430857,-4.4689957985,-1.6675574798
H,1.9631489807,-4.6381763466,-2.4278556687
H,0.5217006563,-5.3311919785,-1.6683314699
H,0.620424697,-3.5890388662,-1.9673666212
C,-1.382538826,-4.1074631673,2.2410254933
H,-2.1823403474,-4.0738643974,2.986464064
H,-0.7520920053,-4.9755334038,2.4601447109
H,-0.7781436785,-3.204903261,2.3662251034
C,-5.2503456564,-5.2109013981,-0.6841760304
H,-4.2691168564,-5.5519578479,-1.0280841883
H,-5.8743724222,-6.0933925297,-0.5107810065
H,-5.6998504605,-4.6289002215,-1.4938127087
C,5.9899271245,-1.462984177,-0.2298240927
C,7.1724072023,-1.1047252584,-0.2419975232
C,-6.0992649705,-1.4193122441,-0.0591291915
C,-7.2414950898,-1.043467733,-0.2107938535
C,-8.5888561183,-0.6055183018,-0.3905778848
C,-9.6620589107,-1.4367927768,-0.0343587559
C,-8.8631440832,0.661964241,-0.9268954349
C,-10.9742373081,-1.0171051315,-0.2083090795
H,-9.45900418,-2.4172442108,0.3818910629
C,-10.1719169503,1.0916618612,-1.1024290031
H,-8.0399418227,1.3088506213,-1.2093743594
C,-11.2021984473,0.2412211798,-0.7389149088
H,-11.8134366485,-1.648000008,0.061649581
H,-10.3996771524,2.0674406834,-1.516135554
C,8.5163949275,-0.6982629533,-0.2563998383
C,9.5591535188,-1.6519033396,-0.2868665629
C,8.8539651907,0.6742807023,-0.2421045431
C,10.8846245511,-1.2509146987,-0.302296676
H,9.311768324,-2.7074981998,-0.2982307148
C,10.178735065,1.0771491748,-0.2570395844
H,8.0618524903,1.4141977305,-0.2189280524
C,11.1701383542,0.1068012272,-0.2870677033

H,11.6955929872,-1.9699157651,-0.3257796782
H,10.4536571995,2.1257010341,-0.2461218558
C,4.6908875328,-1.8551059153,-0.2147723979
C,-4.7705651658,-1.8654155154,0.119245218
F,-12.4785365072,0.6551212004,-0.9087666874
F,12.4625476246,0.4987157563,-0.3019073553

2c-4·Cl(S_0)

(CAM-B3LYP/6-31+G(d,p) for C, H, N, O, and F and CAM-B3LYP/LanL2DZ for Pt)

-2869.0756491 hartree
C,6.7437831647,0.7942203309,1.1963382584
C,7.8348442939,1.6347918405,1.4416449917
H,8.787914891,1.2272757836,1.7674129998
C,7.7035762218,3.0040725854,1.2653584609
H,8.5457081621,3.6629261191,1.4531298594
C,6.4782326195,3.5237770889,0.8394327768
H,6.3708747152,4.5954782969,0.6931947081
C,5.3950744234,2.6863078011,0.5977276873
H,4.455479911,3.1013624349,0.2613165497
C,1.5368366074,-1.547046591,-0.1828365074
C,0.8901308369,-0.3189776175,-0.3974643435
C,1.4370544142,0.9691577849,-0.3358037158
C,5.495322936,1.3033685982,0.7729899644
C,5.4929361301,-2.6007501935,1.1189014737
C,6.7855517426,-0.6585385146,1.3401938797
C,6.5469672641,-3.407265272,1.510417298
C,7.8795560031,-1.4278446879,1.7410648062
C,7.762429724,-2.8053492385,1.8267333493
H,8.8148796792,-0.9381146434,1.9827584561
H,8.6111738812,-3.4063766124,2.1380330416
N,5.6105698785,-1.2692885917,1.0359887547
Pt,4.0939530943,-0.0610256523,0.4799629346
H,4.5173473616,-2.9883246406,0.8581866159
H,6.4099637777,-4.4807345451,1.5644166126
O,2.7584785801,-1.6846855109,0.1892629603
O,2.6584919665,1.2768359399,-0.0428216499
C,0.5550787276,2.1105503263,-0.6188037088
C,0.7177085783,3.4783627384,-0.3553827898
N,-0.6847536348,1.9423418644,-1.1814482969
C,-0.4638368966,4.1309260128,-0.7740660201
H,-1.1286748089,1.0733244431,-1.518323407
C,0.77549826,-2.7884407194,-0.3802492264
C,1.1002548257,-4.115087566,-0.0560667289
N,-0.46692992,-2.7972628687,-0.9588735663
C,0.0114269928,-4.9234360631,-0.4515679495
C,-0.1211052675,-6.4138238415,-0.3418243705
H,0.3164842796,-6.7591357434,0.6023147457
H,-1.1850750732,-6.6704403056,-0.2968415191
C,2.3346151896,-4.6341700262,0.6228485745
H,3.2182969778,-4.2460571885,0.1095731471
H,2.3654906638,-5.7225221367,0.5030551755
C,1.904143706,4.1872300705,0.2285687213
H,2.4619161543,3.5040554133,0.8707250062
H,1.5521450485,5.0081016493,0.8648525143
C,-0.8018356708,5.5872909378,-0.6495073519
H,0.1052684744,6.1907097519,-0.7651910159
H,-1.4673343697,5.871180072,-1.4724671875
C,-1.4776401612,5.9396824478,0.6828330556
H,-0.8334788079,5.6828113854,1.5295301545
H,-1.7061637379,7.0099313916,0.7400179225
H,-2.4123506047,5.3834644937,0.7983009186
C,2.8293264137,4.7586360475,-0.854710402
H,2.2874447782,5.4514626955,-1.506094515
H,3.6734308335,5.3006849225,-0.413382663
H,3.2256505719,3.9562860617,-1.4828711339
C,2.4025659537,-4.2966731927,2.116911969
H,1.5308594568,-4.6998292226,2.6416932425
H,3.3011935888,-4.7208221628,2.5804717585
H,2.4161491907,-3.214627231,2.267818649
C,0.5258616946,-7.1714183064,-1.5090352072
H,1.5962997125,-6.9527952031,-1.5756468274
H,0.4052447357,-8.2544789983,-1.3937416952
H,0.0696658519,-6.8753707561,-2.4580915442
C,-2.5995615065,3.2499629175,-1.8514661287
C,-3.702113565,3.2428522981,-2.3497476214
C,-2.2021561719,-4.3538018675,-1.5823593101
C,-3.2906684765,-4.4985166858,-2.0902697738
C,-4.5735716874,-4.4985394308,-2.7140731632
C,-5.3082483478,-5.6756225307,-2.9086874638
C,-5.1091153564,-3.2721769918,-3.1453229911
C,-6.5555505408,-5.6392031921,-3.5224874883
H,-4.8958145093,-6.6221413981,-2.5758901617
C,-6.3543390522,-3.2308517364,-3.7588640545
H,-4.5298338307,-2.365249234,-2.9885707568
C,-7.0508075446,-4.4149299194,-3.9346078311
H,-7.1392536685,-6.5389383868,-3.68215541
H,-6.7861885085,-2.2967993592,-4.1003377337
C,-4.9854337812,3.0648570809,-2.9464954573
C,-5.8400714006,4.1430110094,-3.2122311176
C,-5.3973593513,1.7616189644,-3.2762268396
C,-7.0847831964,3.9338616488,-3.7960447925
H,-5.5229506982,5.1488867297,-2.9585113224
C,-6.639430262,1.5477817834,-3.8595811838
H,-4.7260145514,0.9326290394,-3.0651356628
C,-7.456529508,2.6381672482,-4.1072258175
H,-7.7599593862,4.7550703464,-4.0093496648
H,-6.9774825478,0.5515816381,-4.1220313733
C,-1.3074441074,3.1478248731,-1.286715704
C,-0.9389379104,-4.0716398302,-1.0132039703
H,-1.0041058694,-2.0042099796,-1.3484961622
F,-8.2696107531,-4.3755971861,-4.5355530732
F,-8.6725771685,2.4298128854,-4.6786640287
H,-0.1611558275,-0.3727546726,-0.6324775024
Cl,-2.3982812019,-0.5724141275,-2.1719971105

[S4 (the complete form of ref. 37 in the manuscript)] Frisch, M. J.; Trucks, G. W.; Schlegel, H. B.; Scuseria, G. E.; Robb, M. A.; Cheeseman, J. R.; Scalmani, G.; Barone, V.; Petersson, G. A.; Nakatsuji, H.; Li, X.; Caricato, M.; Marenich, A. V.; Bloino, J.; Janesko, B. G.; Gomperts, R.; Mennucci, B.; Hratchian, H. P.; Ortiz, J. V.; Izmaylov, A. F.; Sonnenberg, J. L.; Williams-Young, D.; Ding, F.; Lipparini, F.; Egidi, F.; Goings, J.; Peng, B.; Petrone, A.; Henderson, T.; Ranasinghe, D.; Zakrzewski, V. G.; Gao, J.; Rega, N.; Zheng, G.; Liang, W.; Hada, M.; Ehara, M.; Toyota, K.; Fukuda, R.; Hasegawa, J.; Ishida, M.; Nakajima, T.; Honda, Y.; Kitao, O.; Nakai, H.; Vreven, T.; Throssell, K.; Montgomery, J. A., Jr.; Peralta, J. E.; Ogliaro, F.; Bearpark, M. J.; Heyd, J. J.; Brothers, E. N.; Kudin, K. N.; Staroverov, V. N.; Keith, T. A.; Kobayashi, R.; Normand, J.; Raghavachari, K.; Rendell, A. P.; Burant, J. C.; Iyengar, S. S.; Tomasi,

J.; Cossi, M.; Millam, J. M.; Klene, M.; Adamo, C.; Cammi, R.; Ochterski, J. W.; Martin, R. L.; Morokuma, K.; Farkas, O.; Foresman, J. B.; Fox, D. J. *Gaussian 16, Revision C.01*, Gaussian, Inc., Wallingford CT, 2016.

[S5] Phipps, M. J. S.; Fox, T.; Tautermann, C. S.; Skylaris, C.-K. Energy decomposition analysis approaches and their evaluation on prototypical protein–drug interaction patterns. *Chem. Soc. Rev.* **2015**, *44*, 3177–3211.

[S6] Articles for *GAMESS*: (a) Schmidt, M. W.; Baldridge, K. K.; Boatz, J. A.; Elbert, S. T.; Gordon, M. S.; Jensen, J. H.; Koseki, S.; Matsunaga, N.; Nguyen, K. A.; Su, S.; Windus, T. L.; Dupuis, M.; Montgomery, J. A. General Atomic and Molecular Electronic Structure System. *J. Comput. Chem.* **1993**, *14*, 1347–1363. (b) Gordon, M. S.; Schmidt, M. W. Advances in electronic structure theory: GAMESS a decade later. In *Theory and Applications of Computational Chemistry: the first forty years*; Dykstra, C. E., Frenking, G., Kim, K. S., Scuseria, G. E. Eds.; Elsevier, 2005, pp. 1167–1189.

[S7] Report for FMO: Kitaura, K.; Ikeo, E.; Asada, T.; Nakano, T.; Uebayasi, M. Fragment molecular orbital method: an approximate computational method for large molecules. *Chem. Phys. Lett.* **1999**, *313*, 701–706.

[S8] Report for pair interaction energy decomposition analysis (PIEDA): Fedorov, D. G.; Kitaura, K. Pair Interaction Energy Decomposition Analysis. *J. Comput. Chem.* **2007**, *28*, 222–237.

4. Anion-binding behaviors

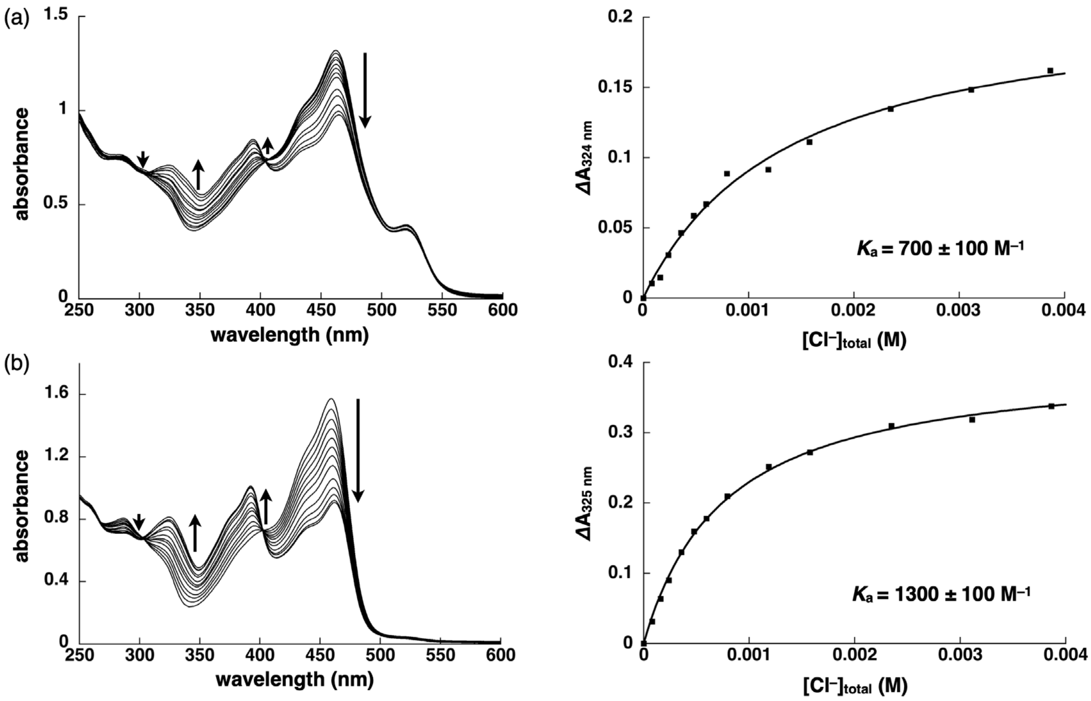

Figure S30 UV/vis absorption spectral changes (left) and corresponding titration plots and 1:1 fitting curves (right) of (a) **2b** and (b) **2c** upon the addition of Cl^- as a tetrabutylammonium (TBA) salt in CH_2Cl_2 (0.02 mM). Due to the observation of slightly decomposed species during titration experiment, the K_a values should be roughly estimated values.

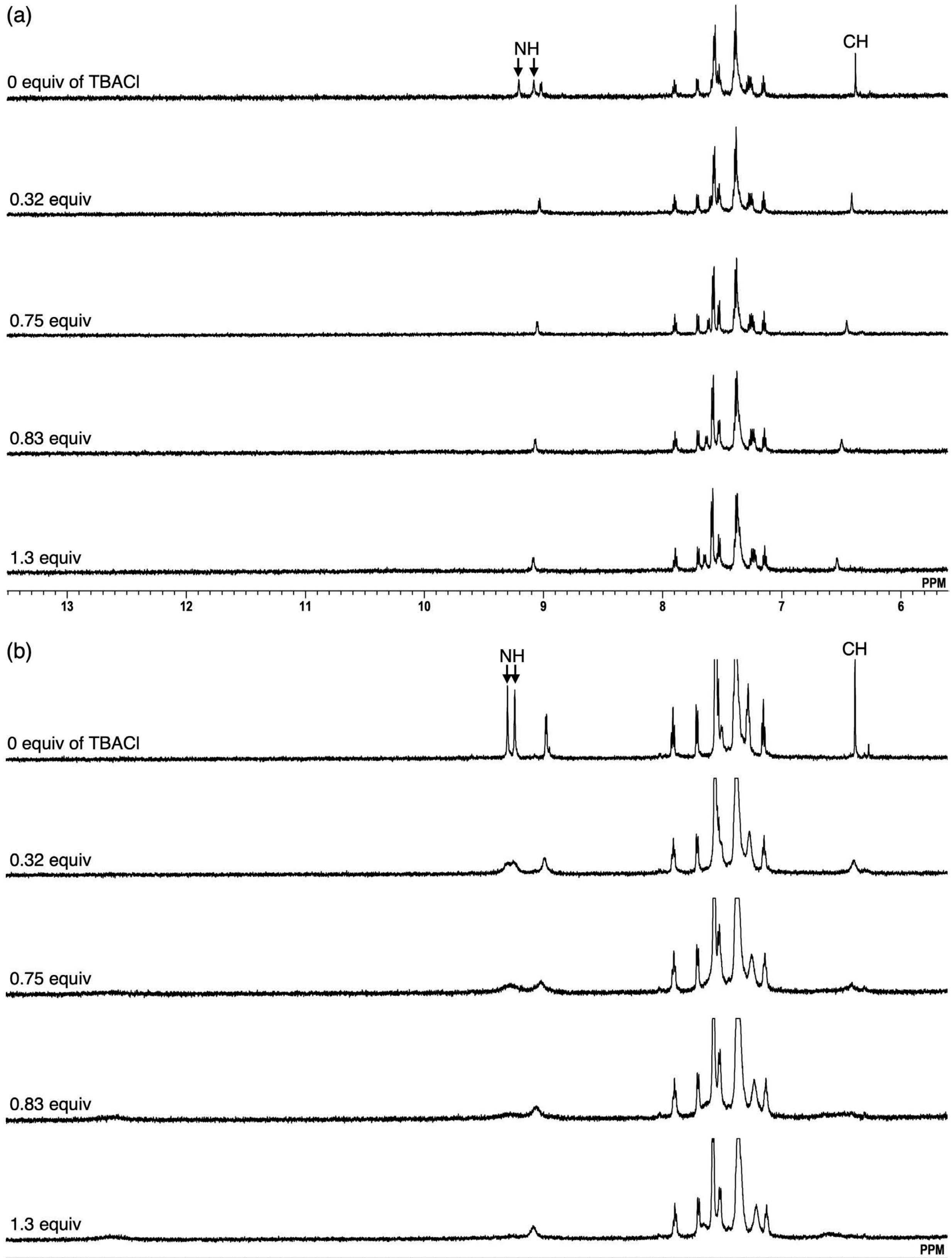

Figure S31 ^{1}H NMR spectral changes of **2b** (1.0 mM) upon the addition of Cl^- (0–1.3 equiv) added as a TBA salt in CD_2Cl_2 at (a) 20 °C and (b) –50 °C. Upon the addition of Cl^-, initial signals of pyrrole NH (9.29 and 9.23 ppm) and bridging CH (6.38 ppm) decreased and new signals were emerged in the downfield regions, whose signals were identified as anion-binding pyrrole NH (12.62 ppm) and bridging CH (6.61 ppm) with 1.3 equiv of TBACl at –50 °C.

5. Solution-state excited-state properties

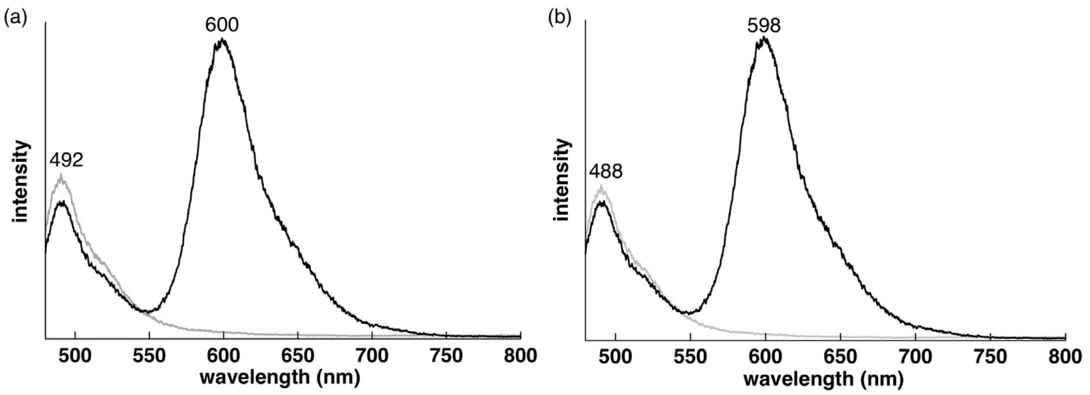

Figure S32 Emission spectra of (a) **2b** (λ_{ex} = 462 nm) and (b) **2c** (λ_{ex} = 460 nm) in deoxygenated CH_2Cl_2 (black) and non-deoxygenated CH_2Cl_2 (gray) (1.9 μM for each) at r.t. Emission peaks at 492 and 488 nm are ascribed to partially decomposed products.

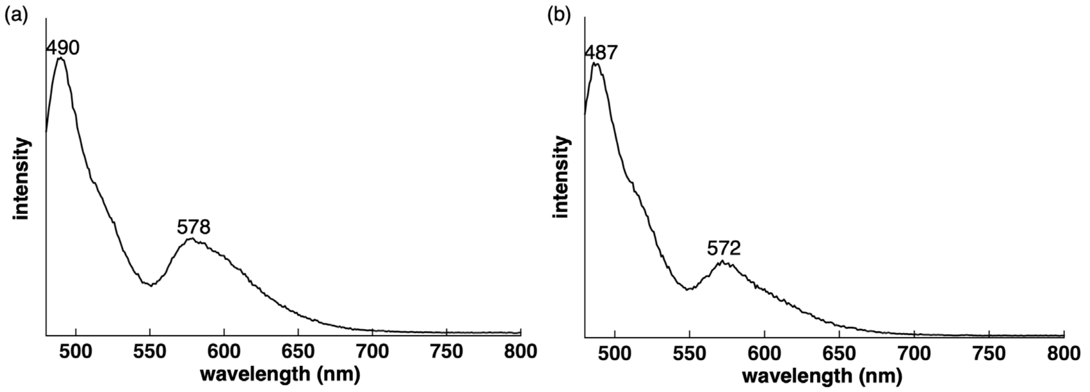

Figure S33 Emission spectra of (a) **2b** (λ_{ex} = 462 nm) and (b) **2c** (λ_{ex} = 460 nm) upon the addition of 3000 equiv of Cl^- as a TBA salt in deoxygenated CH_2Cl_2 (1.9 μM for each) at r.t. Emission peaks at 490 and 487 nm are ascribed to partially decomposed products.

Table S5 Emission quantum yields of **2b**,**c** in non-deoxygenated and deoxygenated CH_2Cl_2 at 20 °C.

solution conditions	**2b**	**2c**	**2b**·Cl^-	**2c**·Cl^-
non-deoxygenated CH_2Cl_2	0.006	0.004	–	–
deoxygenated CH_2Cl_2	0.113	0.138	0.116	0.189

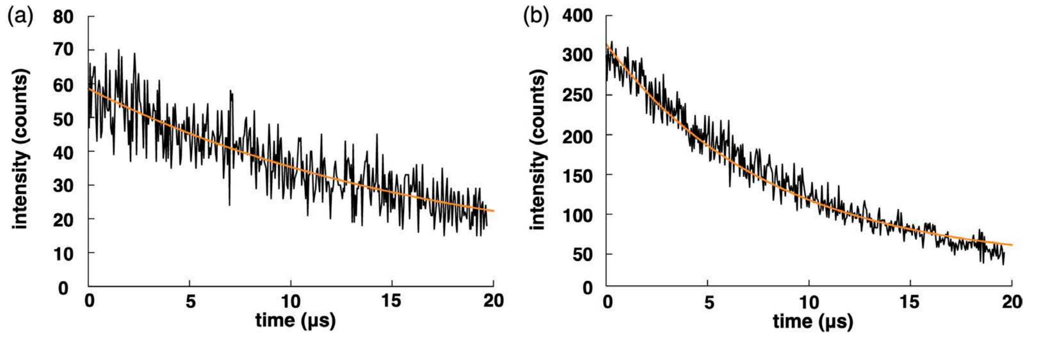

Figure S34 Emission decay profiles of (a) **2b** and (b) **2c** excited at 403 nm in deoxygenated CH_2Cl_2 at 20 °C. The emission decay profiles were fitted with the single exponential decay function, providing the emission lifetimes of 17.3 and 8.0 μs for **2b**,**c**, respectively.
